# Diarylamine-Guided Carboxamide Derivatives: Synthesis, Biological Evaluation, and Potential Mechanism of Action

**DOI:** 10.3389/fchem.2022.953523

**Published:** 2022-07-12

**Authors:** Shaoyong Ke, Wenbo Huang, Zhigang Zhang, Yueying Wang, Yani Zhang, Zhaoyuan Wu, Wei Fang, Zhongyi Wan, Yan Gong, Jingzhong Yang, Kaimei Wang, Liqiao Shi

**Affiliations:** Key Laboratory of Microbial Pesticides, Ministry of Agriculture and Rural Affairs, National Biopesticide Engineering Research Centre, Hubei Biopesticide Engineering Research Centre, Hubei Academy of Agricultural Sciences, Wuhan, China

**Keywords:** diarylamine, diamides, carboxamide, synthesis, bioactivity

## Abstract

Diarylamines are a class of important skeleton widely existing in drugs or natural products. To discover novel diarylamine analogues as potential drugs, two series of diamide and carboxamide derivatives containing diarylamine scaffold were designed, synthesized and evaluated for their potential cytotoxic activities. The bioassay results indicated that some of the obtained compounds (C5, C6, C7, C11) exhibited good cytotoxic effect on cancer cell lines (SGC-7901, A875, HepG2), especially, compound C11 present significantly selective proliferation inhibition activity on cancer and normal cell lines (MARC145). In addition, the possible apoptosis induction for highly potential molecules was investigated, which present compound C11 could be used as novel lead compound for discovery of promising anticancer agents.

## 1 Introduction

According to the progress report published by American Association for Cancer Research in 2019, cancer has become the leading cause of morbidity and mortality around the world, accounting for about 16 percent of deaths worldwide (American Cancer Society, 2018; AACR, 2019). Annual cancer cases and deaths have been increasing since 2000, and in 2018, 24% of estimated new cancer cases and 30% of cancer-related deaths globally occurred in China (Li et al., 2019). It is reported that the most common causes of cancer death were lung cancer, liver cancer and stomach cancer in China (Chen et al., 2016). The traditional treatment for cancer includes surgical resection combined with radiotherapy and chemotherapy; however, there are adverse drug reactions or therapeutic resistance. The devastating impact of cancer is predicated to grow significantly in the coming decades unless new and more effective anticancer drugs are developed. From 2009 to 2018, the number of cancer drug trails in China showed remarkable growth, with an average annual growth rate of 33% (Li et al., 2019). The China cancer drugs market size is projected to reach USD 30 billion by the year 2024. Thus, there is an urgent need for developing new anticancer agents with safer toxicological profiles and novel modes of action.

**FIGURE 1:**
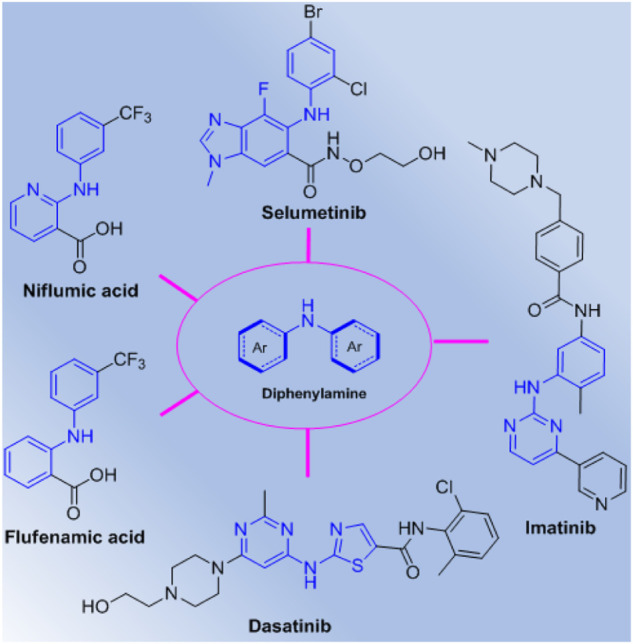
Some typical diarylamine derivatives as drugs.

Diarylamines are a class of important scaffold widely existing in drugs and natural products, which can construct diverse molecular structures with extensive pharmacological activity (Kumar and Mishra, 2018; Abduelkarem et al., 2020). Diarylamines have also been used as typical synthons for construction of novel molecules due to their special multifunctional features, and many diarylamines analogues have been demonstrated to exhibit broad range of biological functions (Guan et al., 2013; Wang et al., 2013; Chai et al., 2014; Soussi et al., 2014; Wild et al., 2016; Wang et al., 2017; Zhang et al., 2017). Especially, some important diarylamine derivatives such as selumetinib, imatinib, dasatinib, niflumic acid, and flufenamic acid have been discovered and developed as commercial drugs (Figure 1), which further identify that this special scaffold is an attractive pharmacophore in the discovery of highly potential molecules, and will lead to some promising drug candidates. Therefore, diarylamines as easily available substrates have proven a convenient choice for construction of structural diversity molecules with promising bioactivity. In addition, carboxamides are also a useful class of molecules with potential pharmacological (Odingo et al., 2017; Tang et al., 2018; Barker et al., 2019; Ling et al., 2019; Sethy et al., 2019; Sun et al., 2019; Yang et al., 2019) and agroactive activities (Sartori et al., 2018; Tsikolia et al., 2018; Yu et al., 2018; Liu et al., 2019; Siber et al., 2019; Tsikolia et al., 2019; Liu et al., 2020), and many carboxamides have been developed as clinical drugs or agrochemicals, which demonstrate that amide units will play an important role in the development of drugs and pesticides.

**FIGURE 2:**
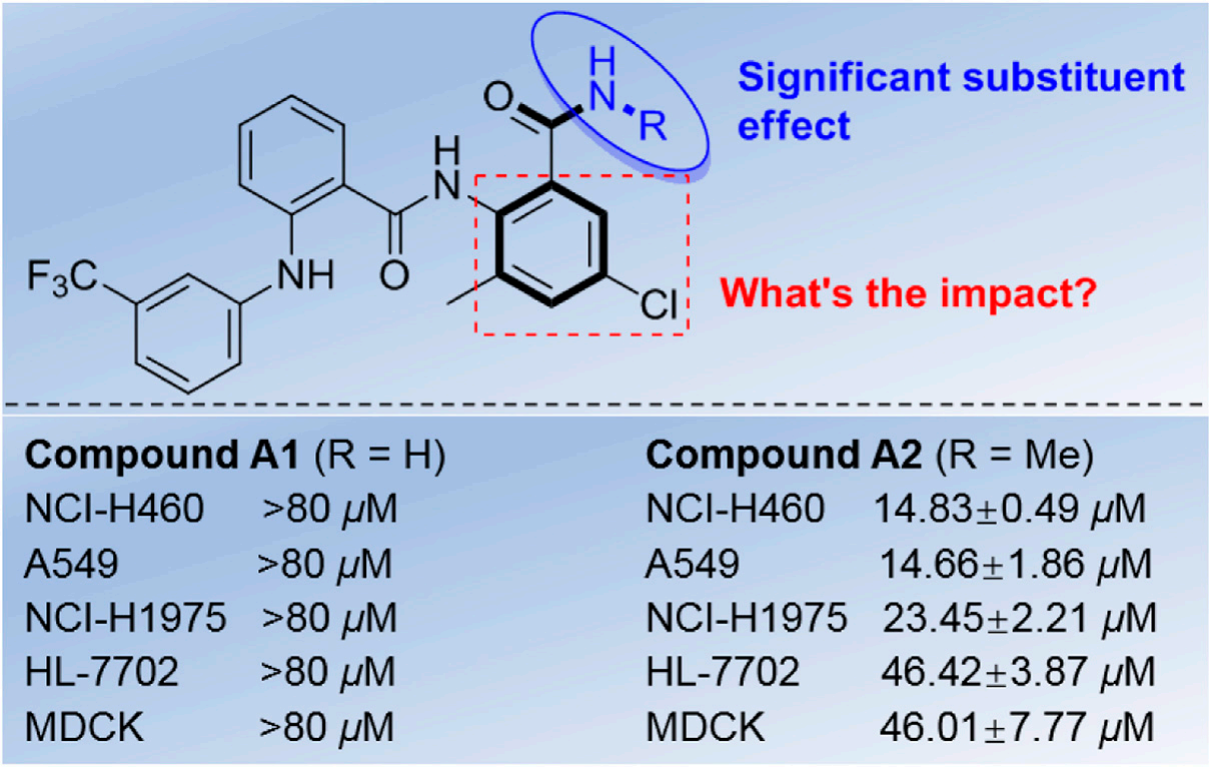
Diarylamine-guided diamide derivatives as lead molecules.

Recently, during the course of our research on discovery of functional molecules, a series of diamides derivatives bearing nicotinamide unit were obtained (Peng et al., 2017), and an interesting phenomenon was found based on the preliminary results. All of the similar diamides without nicotinamide moiety lost the inhibitory activity except the compound bearing diarylamine unit (**A2**, R = Me), and this compound exhibited good cytotoxic activity against the tested lung cancer cell lines (NCI-H460, A549, and NCI-H1975) with IC_50_ values of 14.66–46.42 μM (Figure 2). However, the corresponding *N*-unsubstituted diarylamine-amide derivative **A1** (R = H) almost lost activity (IC_50_ > 80 μM). These interesting findings may provide some useful information for developing diarylamine derivatives as potential anticancer agents, which also urge us to investigate the possible structure and activity relationships (SARs) for these diarylamine derivatives bearing diamide scaffold.

**FIGURE 3:**
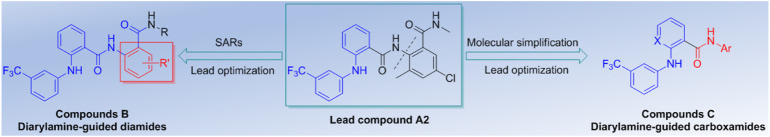
Design strategies for diarylamine-guided amide derivatives.

So, a series of extended diarylamine-guided diamides analogues based on the lead compound **A2** was constructed for exploring prospective SARs with good cytotoxic activity as shown in Figure 3, and a series of diarylamine-guided carboxamide derivatives have also been designed for comparison with that of diarylamine-guided diamides. We report herein the synthesis and characterization of two series of diarylamine derivatives that have flexible substituent patterns, and their *in vitro* cytotoxic activities against SGC-7901 (human gastric cancer cell line), A875 (human melanoma cell line), HepG2 (human hepatocellular liver carcinoma cell line) and MARC145 (A subclone of African green monkey kidney cell line MA-104) cell lines were fully investigated. The results demonstrated that compound **C11** may be a highly potential selective cytotoxic agent between cancer and normal cell lines.

## 2 Results and Discussion

### 2.1 Chemistry

**Scheme 1:**
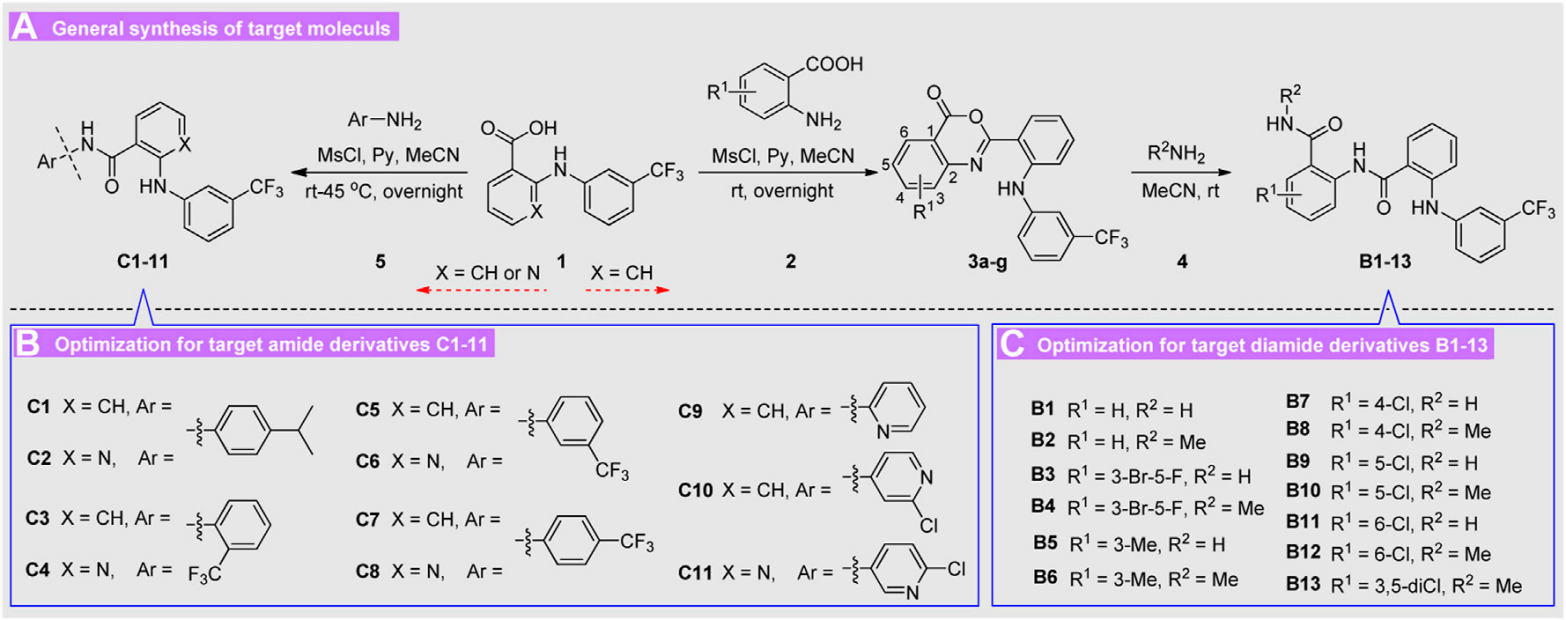
General synthetic route for target molecules **B1-13** and **C1-11**.

According to the structure of lead molecule **A2**, various *ortho*-amino aryl acid **2** and diverse substituted aryl amines **5** were utilized as the building blocks to construct these diarylamine guided carboxamide or diamide derivatives. The target molecules **B1-13** and **C1-11** were conveniently prepared as described in Scheme 1.

First, the easily available flufenamic acid **1** (X = CH) was used as starting material for construction of diarylamine-guided diamides **B1-13**, which were reacted with various *ortho*-amino aryl acid **2** to obtain the corresponding oxazinone heterocyclic intermediates. Then these oxazinones **3** were treated with various amines resulting in the target diamides derivatives **B1-13** *via* nucleophilic substitution reaction. Subsequently, diverse substituted arylamines **5** were directly adopted to construction diarylamine guided carboxamides **C1-11** *via* nucleophilic substitution reaction of acids **1** (X = CH or N). The structures of all obtained molecules were characterized on the basis of satisfied spectral analysis, and the substituents of all the compounds were present in Scheme 1. All of the newly prepared diamides **B1-13** and carboxamide derivatives **C1-11** were confirmed by IR, ^1^H NMR, ^13^C NMR and ESI-MS analyses, and their chemical structures and basic physicochemical properties were summarized in *Experimental*. The ^1^H NMR of these compounds indicated typical proton signal peaks for different groups, and the ESI-MS spectrum indicated all these diarylamine-guided diamide derivatives **B1-13** displayed an obvious [M + Na]^+^ addition ion fragmentation peak, however, the MS for diarylamine-containing carboxamide derivatives **C1-11** exhibited [M + H]^+^ or [M−H]^+^ ion fragmentation peak.

### 2.2 Biological Evaluation

The lead molecules **A2** and all newly prepared diarylamine derivatives **B1-13** and **C1-11** were evaluated for their *in vitro* cytotoxic effects against SGC-7901, A875, HepG2 and MARC145 cell lines by the classical MTT [3-(4,5-dimethylthiazol-2-yl)-2,5-diphenyl tetrazolium bromide] assay (Alley et al., 1988; Ke et al., 2012; Shi et al., 2012; Yu et al., 2015; Ke et al., 2019; Xu et al., 2019; Huang et al., 2021) using 5-FU (5-Fluorouracil) as a control.

First, the lead molecule **A2** exhibit good *in vitro* cytotoxic activity against lung cancer cell lines including NCI-H460, A549, and NCI-H1975 based on our previous investigation (Peng et al., 2017). As far as we know, the substituents attached to aryl ring usually have obvious influence on the activity. So, in order to investigate the possible effect of this unit on the activity of final molecules, a series of diarylamine guided diamide derivatives **B1-13** were extended synthesized, and their cytotoxic activity was tested against SGC-7901, A875, HepG2, and MARC145 cell lines. Unfortunately, all newly prepared diarylamine-guided diamide derivatives **B1-13** present poor effects on these tested cell lines as indicated in the Table 1, however, the lead compound **A2** still exhibit very good cytotoxicity with IC_50_ values of 7.59–12.43 μM. These results demonstrated that the change of an appropriate substituents on the aryl ring could dramatically change the biological activity.

**TABLE 1:** *In vitro* cytotoxic activities of the diarylamine guided diamide derivatives **B1-13**.

Entry	Compd. No.	*In vitro* cytotoxicity IC_50_ *^a^* (*μ*M)			
		SGC-7901^b^	A875^b^	HepG2^b^	MARC145^b^
1	A1	52.81 ± 6.59	56.90 ± 7.54	>89	>89
2	A2	8.50 ± 2.26	7.59 ± 1.82	12.43 ± 0.39	8.13 ± 2.49
3	B1	>100	>100	>100	>100
4	B2	>96	>96	>96	>96
5	B3	22.54 ± 4.04	>80	>80	38.02 ± 0.81
6	B4	>78	>78	>78	>78
7	B5	>97	>97	>97	>97
8	B6	>94	>94	>94	>94
9	B7	>92	>92	>92	>92
10	B8	>89	>89	>89	>89
11	B9	>92	>92	>92	>92
12	B10	39.36 ± 8.96	>89	49.92 ± 9.26	37.98 ± 9.84
13	B11	>92	>92	>92	>92
14	B12	>89	>89	>89	>89
15	B13	>83	>83	>83	>83
16	5-FU^c^	71.06 ± 2.65	82.38 ± 23.65	88.09 ± 17.23	153.24 ± 11.01

a IC_50_—Compound concentration required to inhibit tumor cell proliferation by 50%.

b Abbreviations: SGC-7901—Human gastric cancer cell line; A875—Human melanoma cell line; HepG2—Human hepatocellular liver carcinoma cell line; MARC145—A subclone of African green monkey kidney cell line MA-104.

c Used as a positive control.

**FIGURE 4:**
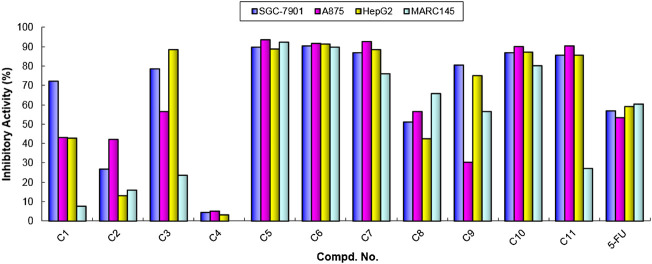
Cytotoxic activities of compounds C1-11 at the concentration of 100 µM. Abbreviations: SGC-7901—Human gastric cancer cell line; A875—Human melanoma cell line; HepG2—Human hepatocellular liver carcinoma cell line; MARC145—A subclone of African green monkey kidney cell line MA-104; 5-FU—5-Fluorouracil, used as a positive control.

With these results in hand, we think that the part of *ortho*-amino aryl acid may be important for the bioactivity, and so the molecular simplification strategy was adopted to investigate the potential active molecules. As shown in Scheme 1, a series of diarylamine guided carboxamides **C1-11** were further constructed using diverse substituted aryl amines, and the preliminary screening for these molecules were treated at the concentration of 100 µM (Table 2). What’s interesting is that some of the compounds (**C5**, **C6**, **C7**, **C10**, and **C11**) exhibit good inhibition activity, and especially the inhibition rate is even higher than that of the control 5-FU (Figure 4). We also can find from Figure 4 that compound **C11** indicated good inhibition on all three cancer cell lines (SGC-7901, A875, and HepG2), however, which has low inhibition on normal cell lines (MARC145).

**TABLE 2:** Cytotoxic activity (% cell growth inhibition) of compounds C1-11 at 100 µM.

Entry	Compd. No.	Cell growth inhibition (%)			
		SGC-7901^a^	A875^a^	HepG2^a^	MARC145^a^
1	C1	72.3 ± 3.1	43.1 ± 2.0	42.7 ± 3.1	7.5 ± 2.3
2	C2	26.9 ± 1.8	42.0 ± 2.5	13.1 ± 2.3	15.8 ± 1.9
3	C3	78.6 ± 2.9	56.4 ± 2.5	88.5 ± 3.2	23.5 ± 2.1
4	C4	4.5 ± 0.4	5.2 ± 1.5	3.1 ± 1.2	0 ± 0
5	C5	89.6 ± 5.4	93.7 ± 3.2	88.9 ± 1.9	92.3 ± 4.1
6	C6	90.5 ± 3.8	91.5 ± 2.9	91.3 ± 3.7	89.8 ± 3.4
7	C7	87.0 ± 2.4	92.6 ± 4.9	88.5 ± 3.3	76.1 ± 3.5
8	C8	51.2 ± 3.5	56.5 ± 3.1	42.4 ± 2.6	65.8 ± 2.6
9	C9	80.5 ± 2.5	30.3 ± 4.1	75.0 ± 2.7	56.5 ± 3.3
10	C10	86.8 ± 3.2	90.1 ± 5.4	87.2 ± 4.1	80.3 ± 4.6
11	C11	85.6 ± 4.3	90.5 ± 3.7	85.7 ± 2.4	27.1 ± 3.6
12	5-FU^b^	56.8 ± 2.2	53.2 ± 4.5	59.0 ± 3.5	60.3 ± 3.9

a Abbreviations: SGC-7901—Human gastric cancer cell line; A875—Human melanoma cell line; HepG2—Human hepatocellular liver carcinoma cell line; MARC145—A subclone of African green monkey kidney cell line MA-104.

b Used as a positive control.

Base on the aforementioned results, some of the diarylamine-guided carboxamide derivatives have been demonstrated to present good inhibitory activities against all tested cancer cell lines, so in order to further investigate the potential activities, the IC_50_ values were tested based on the above cell-based method. The *in vitro* activities described as IC_50_ values for these compounds were present in Table 3.

**TABLE 3:** *In vitro* cytotoxic activities of the diarylamine guided amide derivatives C1-11.

Entry	Compd. No.	*In vitro* cytotoxicity IC_50_ *^a^* (*μ*M)			
		SGC-7901^b^	A875^b^	HepG2^b^	MARC145^b^
1	C1	47.04 ± 9.74	>100	>100	>100
2	C2	>100	>100	>100	>100
3	C3	29.71 ± 4.03	>94	24.15 ± 3.21	>94
4	C4	>94	>94	>94	>94
5	C5	6.39 ± 1.53	8.54 ± 2.17	10.14 ± 0.80	9.57 ± 2.15
6	C6	5.53 ± 0.82	12.77 ± 1.99	9.99 ± 1.58	9.72 ± 2.75
7	C7	8.91 ± 2.15	13.04 ± 3.39	10.42 ± 2.17	11.86 ± 1.37
8	C8	>94	55.61 ± 6.73	>94	57.70 ± 8.87
9	C9	42.03 ± 7.95	>112	64.24 ± 3.89	64.66 ± 3.02
10	C10	8.31 ± 2.94	26.34 ± 7.39	21.91 ± 1.64	22.68 ± 5.93
11	C11	9.13 ± 3.52	12.34 ± 4.16	10.15 ± 2.04	>102
12	5-FU^c^	87.68 ± 11.01	101.14 ± 7.14	91.76 ± 16.11	99.00 ± 16.42

a IC_50_—Compound concentration required to inhibit tumor cell proliferation by 50%.

b Abbreviations: SGC-7901—Human gastric cancer cell line; A875—Human melanoma cell line; HepG2—Human hepatocellular liver carcinoma cell line; MARC145—A subclone of African green monkey kidney cell line MA-104.

c Used as a positive control.

**FIGURE 5:**
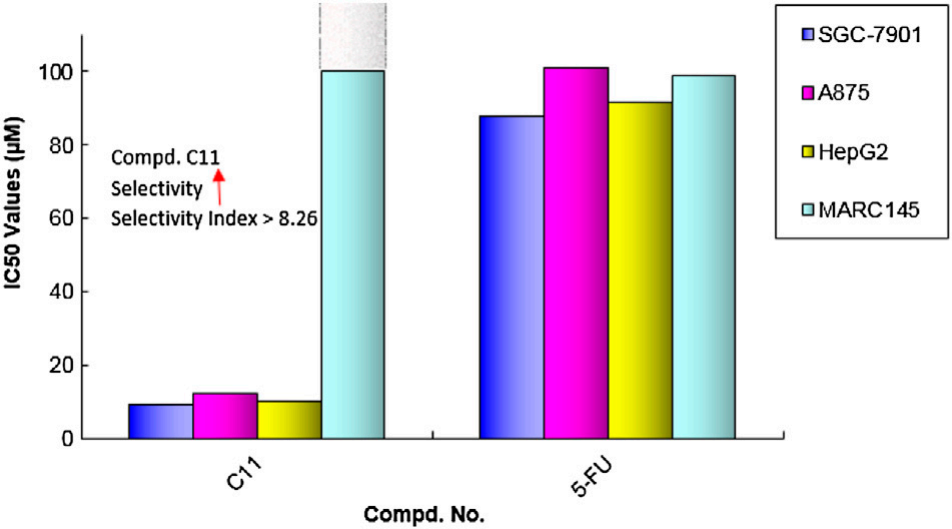
IC_50_ analysis for the highly potential compound **C11** compared with 5-FU.

As shown in Table 3, the results further confirmed that some of these diarylamine-guided carboxamide derivatives (**C5**, **C6**, **C7**, **C10**, and **C11**) indicated higher inhibition activities compared to the control 5-FU. Generally, the compounds bearing *p*-isopropylaniline and *o*-trifluormethylaniline present poor inhibition activities against all tested cell lines (Entries 1–4). The compounds **C6** containing *m*-trifluormethylaniline exhibited significant cytotoxic activities with the IC_50_ values of 5.53–12.77 μM (Entry 6), respectively. However, for the compounds containing *p*-trifluormethylaniline, the compound bearing flufenamic acid (X = CH) present the highest potential activities (Entry 7) than that of the compound containing niflumic acid (X = N). Especially, compound C11 bearing 6-chloropyridin-3-amine had a stronger inhibition on cancer cell lines (SGC-7901 IC_50_ = 9.13 μM; A875 IC_50_ = 12.34 μM; HepG2 IC_50_ = 10.15 μM) than the normal cells line MARC145 (IC_50_ > 102 μM), and it displayed a significantly selective proliferation inhibition activity on cancer and normal cell lines, and the selectivity index up to 8.26 (Figure 5). These interesting finds may provide some useful information for developing potential cytotoxicity agents.

**FIGURE 6:**
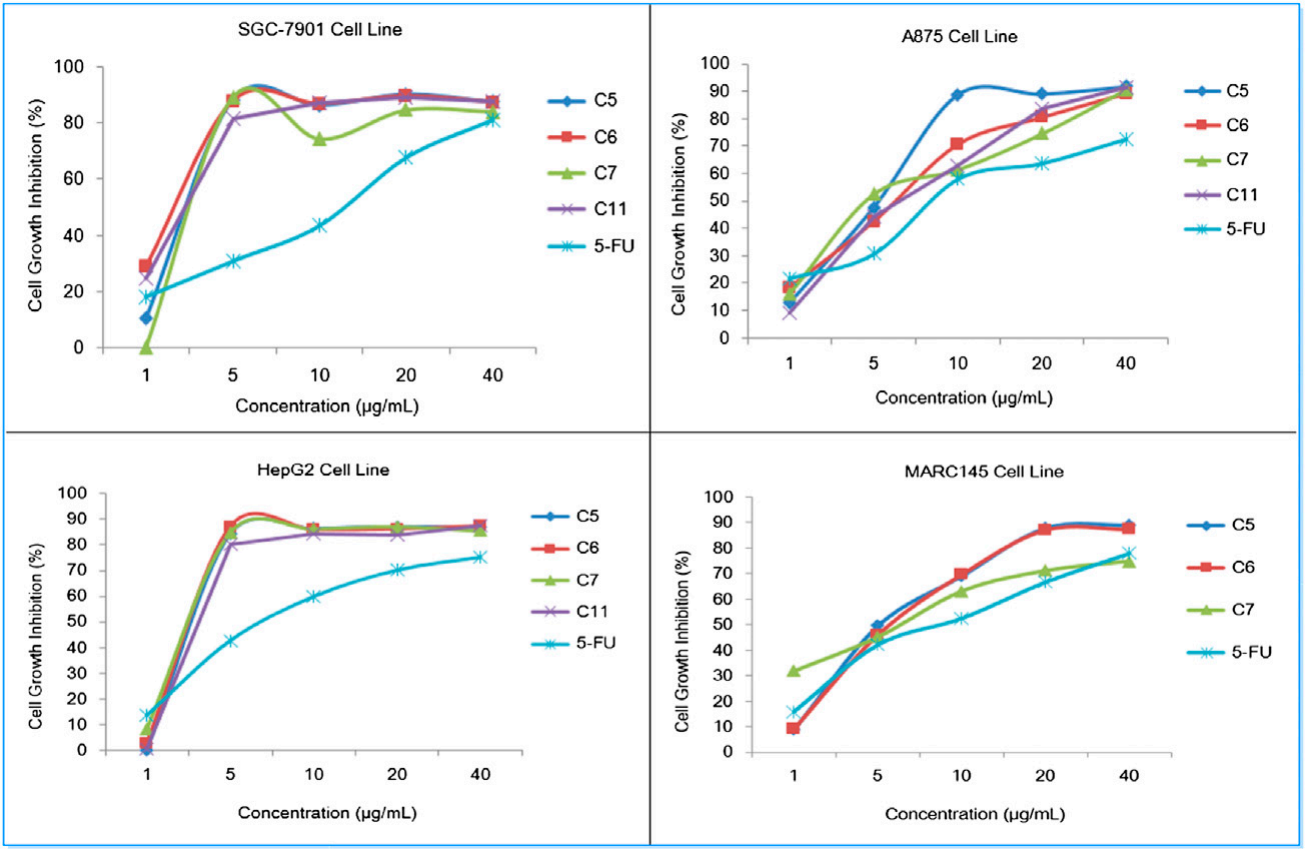
Dose-response analysis of cell growth inhibition activity for compounds **C5**, **C6**, **C7**, and **5-FU** (positive control) against SGC-7901 cells (upper left), A875 cells (upper right), HepG2 cells (lower left) and MARC145 cells (lower right).

In addition, the dose-response analysis of cell growth inhibition for highly potential molecules **C5**, **C6**, **C7**, **C11,** and 5-FU have been displayed in Figure 6, which identified that these compounds exhibited obvious cytotoxic effects on SGC-7901, A875, HepG2, and MARC145 cell lines with a significant concentration dependence.

### 2.3 Mechanism of Action

#### 2.3.1 Analysis for Cell Apoptosis

Apoptosis induction is one of the important modes of action for antitumor drugs. So, the potential mechanism of highly effective compounds (**C6** and **C11**) on A875 cells were investigated by flow cytometry. The apoptotic effect of compounds **C6** and **C11** was evaluated and analyzed following 24 h of treatment with 0.5×IC_50_, IC_50_ or 2×IC_50_ concentrations using Annexin V-FITC/PI dual staining assay.

**FIGURE 7:**
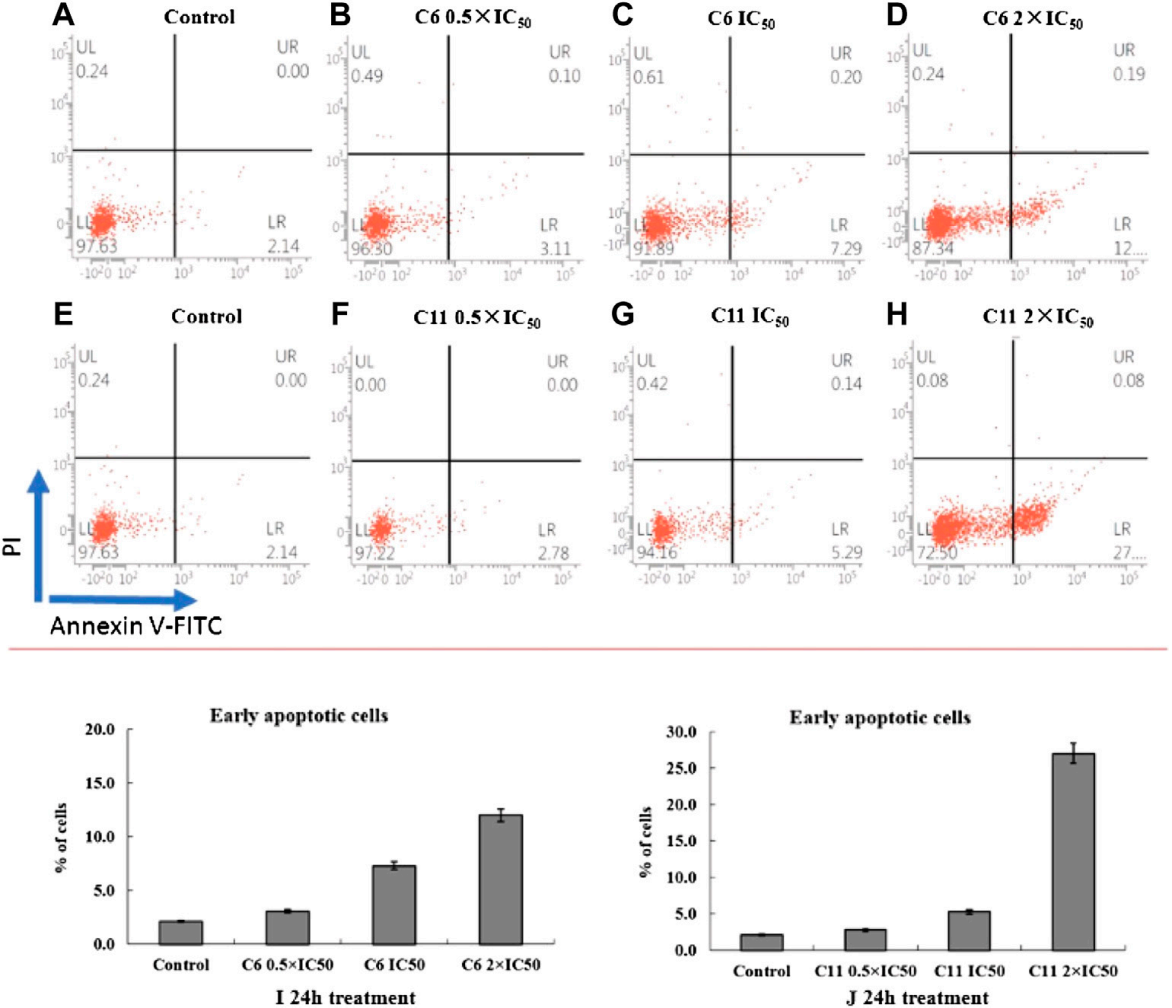
Annexin V-FITC flow cytometry. Annexin V-FITC/PI staining was monitored on A875 cells following 24 h treatment with compounds **C6** or **C11** at concentrations corresponding to their 0.5 × IC_50s_, IC_50s_ or 2 × IC_50s_ **(B–D,F–H)**. Representative dot plots of three independent experiments are given, presenting intact cells at lower-left quadrant, FITC(−)/PI(−); early apoptotic cells at lower-right quadrant, FITC(+)/PI(−); late apoptotic or necrotic cells at upper-right quadrant, FITC(+)/PI(+); necrotic cells at upper-left quadrant, FITC(−)/PI(+). **(I,J)** Apoptotic effect of compounds **C6** and **C11** was evaluated after 24 h treatment; bar graphs represent mean ± SD in at least three independent experiments.

All data obtained in this work are described in Figure 7, and we can find that compounds **C6** and **C11** induced apoptotic changes after 24 h treatment, and both compounds initiated apoptosis, in terms of FITC(+)PI(−) staining, to a larger extent than the control. 12% and 27% apoptotic rates are observed with treatment of compounds **C6** and **C11** at 2×IC_50_ concentrations respectively, whereas 2.14% of apoptosis was observed in control (0.1% DMSO), which indicated that these two potential molecules can induce apoptosis in A875 cells at least partly. However, the clearer mechanism of cell death induction by these compounds still remain to be further investigated.

#### 2.3.2 Determination of Lactate Dehydrogenase

**FIGURE 8:**
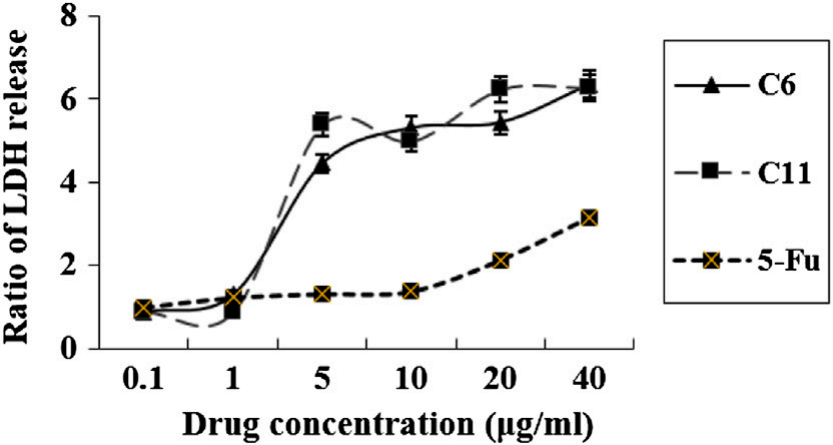
Cellular necrosis was measured as LDH release after treatment for 24 h with the drugs.

From the preliminary analysis of apoptosis, the results encourage us to further investigate the type of cell death induced by the potential compounds (**C6** and **C11**). As we know, the release of LDH is a typical characteristic feature of necrotic cell death, and so which was detected. From the results indicated in Figure 8, we can find that the release of LDH was significantly increased at 24 h after treatment with compounds **C6**, **C11**, and 5-FU (Figure 8), which were consistent with those of flow cytometry.

#### 2.3.3 Kinase Inhibition Assay

Based on the aforementioned results for the potential mechanism of action, the kinase inhibitory profile of compounds **C6** and **C11** were screened at two dose concentration of 10 and 1 *µ*M over a panel of 15 kinases, and the kinase inhibitory activities are listed in supporting information (Supplementary Table S1). It was found that compound **C6** exerted certain inhibitions on the tested MAPK1 and CDK2/A2 protein kinases at 1 μM, and compound **C11** also indicated some inhibitions on the tested CDK1/A2, CDK2/A2, MAPK1 protein kinases at 1 μM, however, the inhibitory activity is not very good. These results will provide some reference for further mechanism research.

## 3 Conclusion

In summary, two series of diarylamine-guided diamide and carboxamide derivatives based on lead molecule have been designed and synthesized, and their potential cytotoxic activities were fully investigated by cell-based assay. The results demonstrated that some of the obtained diarylamine-guided carboxamide derivatives (**C5**, **C6**, **C7**, **C11**) had good cytotoxic effect on cancer cell lines (SGC-7901, A875, HepG2) compared with 5-FU *in vitro*, especially, compound **C11** present significantly selective proliferation inhibition activity between cancer and normal cell lines, and the selectivity index was greater than 8.26, and these interesting results might be helpful to develop diarylamine-guided carboxamides as potential anticancer agents.

## 4 Experimental

### 4.1 Materials and Apparatus

Melting points (m.p.) were measured using a digital model X-5 apparatus (Shanghai Instrument Physical Optics Instrument Co., LTD., Shanghai, China) and were uncorrected. Infrared (IR) Spectra were recorded using Shimadzu FTIR 8400S spectrophotometer. ^1^H NMR and ^13^C NMR spectra were recorded on a Bruker spectrometer at 600 MHz (Bruker, Bremen, Germany) with CDCl_3_, DMSO-*d*_6_ or CD_3_OD as the solvent. Liquid chromatography-tandem mass spectrometry (LC−MS/MS) analysis were performed on a Waters ACQUITY UPLC^®^ H-CLASS PDA (Waters^®^, Milford, MA, United States) instrument. Analytical thin-layer chromatography was carried out on precoated plates, and spots were visualized with ultraviolet light. All chemicals or reagents used for syntheses were commercially available.

### 4.2 General Synthesis of Diamides Derivatives Containing Diarylamine Unit B1-13

All the diarylamine-guided diamides derivatives **B1-13** were prepared according to the similar procedures described in our previous reports (Shi et al., 2012; Peng et al., 2017), and were purified by recrystallization with methanol. The structures of all target compounds were confirmed by their spectral analysis, and all data for target molecules **B1-13** are as following:

N-(2-Carbamoylphenyl)-2-((3- (trifluoromethyl)phenyl)amino)benzamide **B1**. Yield: 63%; m. p. 136–137°C; FTIR υ_max_ (cm^−1^): 3,400, 3,260, 1,643, 1,575, 1,528, 1,334, 1,106; ^1^H NMR (600 MHz, CDCl_3_): *δ* 12.14 (s, 1H), 9.80 (s, 1H), 8.74 (d, *J* = 8.6 Hz, 1H), 7.82 (d, *J* = 7.3 Hz, 1H), 7.61–7.56 (m, 2H), 7.46 (s, 1H), 7.42–7.34 (m, 4H), 7.22 (d, *J* = 5.0 Hz, 1H), 7.15 (t, *J* = 7.2 Hz, 1H), 6.98–6.92 (m, 1H), 6.20 (s, 1H), 5.64 (s, 1H); ^13^C NMR (150 MHz, CDCl_3_): *δ* 171.32, 168.15, 145.22, 142.29, 140.34, 133.53, 132.79, 129.78, 128.31, 127.39, 123.18, 123.03, 121.78, 119.44, 119.08, 118.93, 118.58, 118.55, 116.74, 115.87, 99.99; MS (ESI) *m/z* 422.34 (M + Na)^+^, calcd. for C_21_H_16_F_3_N_3_O_2_ m/z = 399.12.

N-Methyl-2-(2-((3-(trifluoromethyl)phenyl) amino)benzamido)benzamide **B2**. Yield: 67%; m.p. 127–128°C; FTIR υ_max_ (cm^−1^): 3,295, 1,637, 1,596, 1,505, 1,406, 1,280, 1,159; ^1^H NMR (600 MHz, CDCl_3_): *δ* 12.03 (s, 1H), 9.82 (s, 1H), 8.65 (d, *J* = 8.4 Hz, 1H), 7.84 (d, *J* = 7.9 Hz, 1H), 7.57–7.47 (m, 2H), 7.46 (s, 1H), 7.38 (p, *J* = 8.1 Hz, 4H), 7.22 (t, *J* = 6.7 Hz, 1H), 7.13 (t, *J* = 7.6 Hz, 1H), 6.97 (t, *J* = 7.2 Hz, 1H), 6.29 (s, 1H), 3.03 (d, *J* = 4.7 Hz, 3H); ^13^C NMR (150 MHz, CDCl_3_): *δ* 169.74, 168.07, 145.15, 142.35, 139.48, 132.72, 132.61, 131.83, 131.62, 129.78, 128.36, 126.49, 124.96, 123.15, 123.05, 121.86, 121.01, 119.58, 119.16, 118.50, 116.64, 115.86, 100.00, 26.94; MS (ESI) *m/z* 436.38 (M + Na)^+^, calcd. for C_22_H_18_F_3_N_3_O_2_ m/z = 413.14.

3-Bromo-5-fluoro-2- (2-((3-(trifluoromethyl)phenyl)amino)benzamido)benzamide **B3**. Yield: 81%; m.p. 198–199°C; FTIR υ_max_ (cm^−1^): 3,371, 3,263, 1,650, 1,599, 1,504, 1,454, 1,305, 1,115; ^1^H NMR (600 MHz, DMSO-*d*_6_): *δ* 10.21 (s, 1H), 9.37 (s, 1H), 7.85–7.01 (m, 12H); ^13^C NMR (150 MHz, DMSO-*d*_6_): *δ* 168.07, 167.53, 143.30, 143.09, 138.54, 132.76, 131.14, 130.95, 130.04, 125.17, 122.49, 121.59, 121.42, 121.24, 120.18, 117.78, 116.49, 115.38, 115.23, 114.90; MS (ESI) *m/z* 518.21 (M + Na)^+^, calcd. for C_21_H_14_BrF_4_N_3_O_2_ m/z = 495.02.

3-Bromo-5-fluoro-N-methyl-2-(2-((3- (trifluoromethyl)phenyl)amino)benzamido)benzamide **B4**. Yield: 84%; m.p. 189–190°C; FTIR υ_max_ (cm^−1^): 3,281, 1,628, 1,580, 1,456, 1,325, 1,159, 1,122; ^1^H NMR (600 MHz, DMSO-*d*_6_): *δ* 10.26 (s, 1H), 9.14 (s, 1H), 8.39 (s, 1H), 7.84 (d, *J* = 4.5 Hz, 1H), 7.78 (d, *J* = 7.1 Hz, 1H), 7.52–7.38 (m, 6H), 7.23 (d, *J* = 6.4 Hz, 1H), 7.03 (t, *J* = 6.4 Hz, 1H), 2.61 (d, *J* = 2.9 Hz, 3H); ^13^C NMR (150 MHz, DMSO-*d*_6_): *δ* 168.03, 166.26, 153.12, 143.49, 142.54, 135.27, 132.49, 130.92, 129.83, 123.72, 122.35, 121.98, 121.53, 121.36, 120.42, 117.61, 116.85, 115.17, 114.76, 26.54; MS (ESI) *m/z* 532.16 (M + Na)^+^, calcd. for C_22_H_16_BrF_4_N_3_O_2_ m/z = 509.04.

N-(2-Carbamoyl-6-methylphenyl)-2-((3-(trifluoromethyl)phenyl)amino)benzamide **B5**. Yield: 77%; m.p. 191–192°C; FTIR υ_max_ (cm^−1^): 3,377, 3,276, 1,646, 1,596, 1,507, 1,470, 1,289, 1,157; ^1^H NMR (600 MHz, DMSO-*d*_6_): *δ* 10.17 (s, 1H), 9.36 (s, 1H), 7.82–7.80 (m, 2H), 7.48–7.38 (m, 8H), 7.26 (t, *J* = 7.6 Hz, 1H), 7.22 (d, *J* = 7.5 Hz, 1H), 7.02 (t, *J* = 7.8 Hz, 1H), 2.20 (s, 3H); ^13^C NMR (150 MHz, DMSO-*d*_6_): *δ* 170.45, 167.46, 143.71, 142.63, 136.42, 134.48, 133.54, 132.61, 132.44, 130.88, 130.65, 130.44, 129.91, 126.59, 126.35, 122.86, 122.12, 120.63, 117.45, 117.11, 114.49, 18.65; MS (ESI) *m/z* 436.38 (M + Na)^+^, calcd. for C_22_H_18_F_3_N_3_O_2_ m/z = 413.14.

N,3-Dimethyl-2-(2-((3-(trifluoromethyl)phenyl)amino)benzamido)benzamide **B6**. Yield: 82%; m.p. 195–196°C; FTIR υ_max_ (cm^−1^): 3,317, 1,651, 1,611, 1,529, 1,322, 1,158, 1,114; ^1^H NMR (600 MHz, DMSO-*d*_6_): *δ* 10.15 (s, 1H), 9.19 (s, 1H), 8.30 (d, *J* = 4.5 Hz, 1H), 7.75 (dd, *J* = 7.7, 1.1 Hz, 1H), 7.54–7.32 (m, 7H), 7.26 (t, *J* = 7.6 Hz, 1H), 7.21 (d, *J* = 7.4 Hz, 1H), 7.03 (t, *J* = 7.0 Hz, 1H), 2.65 (d, *J* = 4.6 Hz, 3H), 2.22 (s, 3H); ^13^C NMR (150 MHz, DMSO-*d*_6_): *δ* 168.95, 167.47, 143.82, 142.20, 136.38, 134.32, 133.91, 132.47, 132.18, 130.85, 129.73, 126.67, 126.11, 123.69, 121.75, 120.75, 117.23, 26.56, 18.60; MS (ESI) *m/z* 450.33 (M + Na)^+^, calcd. for C_23_H_20_F_3_N_3_O_2_ m/z = 427.15.

N-(2-Carbamoyl-5- chlorophenyl)-2-((3-(trifluoromethyl)phenyl)amino)benzamide **B7**. Yield: 64%; m.p. 176–177°C; FTIR υ_max_ (cm^−1^): 3,345, 3,153, 1,668, 1,640, 1,515, 1,389, 1,325, 1,159, 1,117; ^1^H NMR (600 MHz, DMSO-*d*_6_): *δ* 12.82 (s, 1H), 9.30 (s, 1H), 8.67 (d, *J* = 2.2 Hz, 1H), 8.43 (s, 1H), 7.88 (d, *J* = 8.6 Hz, 2H), 7.74 (dd, *J* = 7.9, 1.4 Hz, 1H), 7.55–7.33 (m, 5H), 7.26 (dd, *J* = 8.5, 2.2 Hz, 1H), 7.21 (d, *J* = 7.6 Hz, 1H), 7.09–7.05 (m, 1H); ^13^C NMR (150 MHz, DMSO-*d*_6_): *δ* 170.54, 167.25, 143.67, 143.14, 141.40, 137.19, 133.30, 130.81, 130.78, 129.13, 125.50, 123.70, 122.95, 122.55, 122.33, 121.17, 120.10, 118.64, 118.50, 117.75, 114.96; MS (ESI) *m/z* 456.27 (M + Na)^+^, calcd. for C_21_H_15_ClF_3_N_3_O_2_ m/z = 433.08.

4-Chloro-N-methyl-2-(2-((3-(trifluoromethyl)phenyl)amino)benzamido)benzamide **B8**. Yield: 71%; m.p. 152–153°C; FTIR υ_max_ (cm^−1^): 3,316, 1,633, 1,572, 1,515, 1,330, 1,221, 1,166, 1,117; ^1^H NMR (600 MHz, DMSO-*d*_6_): *δ* 12.50 (s, 1H), 9.21 (s, 1H), 8.85 (d, *J* = 4.2 Hz, 1H), 8.63 (d, *J* = 2.2 Hz, 1H), 7.77 (d, *J* = 8.5 Hz, 1H), 7.74 (dd, *J* = 7.8, 1.4 Hz, 1H), 7.52–7.48 (m, 1H), 7.45 (t, *J* = 8.2 Hz, 1H), 7.40–7.34 (m, 3H), 7.26 (dd, *J* = 8.5, 2.2 Hz, 1H), 7.19 (d, *J* = 7.6 Hz, 1H), 7.15–7.07 (m, 1H), 2.74 (d, *J* = 4.5 Hz, 3H); ^13^C NMR (150 MHz, DMSO-*d*_6_): *δ* 168.36, 167.06, 143.92, 142.69, 140.71, 136.76, 133.25, 130.73, 130.59, 130.39, 130.10, 129.32, 125.52, 123.27, 123.07, 121.96, 121.54, 120.25, 119.64, 118.99, 114.62, 26.70; MS (ESI) *m/z* 470.32 (M + Na)^+^, calcd. for C_22_H_17_ClF_3_N_3_O_2_ m/z = 447.10.

N-(2-Carbamoyl-4-chlorophenyl) -2-((3-(trifluoromethyl)phenyl)amino)benzamide **B9**. Yield: 68%; m.p. 183–184°C; FTIR υ_max_ (cm^−1^): 3,405, 3,197, 1,643, 1,611, 1,518, 1,385, 1,295, 1,112; ^1^H NMR (600 MHz, DMSO-*d*_6_): *δ* 12.53 (s, 1H), 9.30 (s, 1H), 8.55 (d, *J* = 9.0 Hz, 1H), 8.45 (s, 1H), 7.92 (d, *J* = 2.5 Hz, 1H), 7.90 (s, 1H), 7.73 (dd, *J* = 7.9, 1.4 Hz, 1H), 7.61 (dd, *J* = 9.0, 2.5 Hz, 1H), 7.50–7.45 (m, 2H), 7.41–7.37 (m, 3H), 7.20 (d, *J* = 7.6 Hz, 1H), 7.11–7.04 (m, 1H); ^13^C NMR (150 MHz, DMSO-*d*_6_): *δ* 170.00, 167.02, 143.72, 143.02, 138.85, 133.18, 132.38, 130.82, 130.64, 129.14, 128.75, 127.10, 123.70, 122.78, 122.65, 122.21, 122.14, 121.22, 118.52, 117.69, 114.77; MS (ESI) *m/z* 456.27 (M + Na)^+^, calcd. for C_21_H_15_ClF_3_N_3_O_2_ m/z = 433.08.

5-Chloro-N-methyl-2-(2-((3-(trifluoromethyl)phenyl)amino)benzamido)benzamide **B10**. Yield: 74%; m.p. 190–191°C; FTIR υ_max_ (cm^−1^): 3,300, 1,657, 1,629, 1,515, 1,328, 1,157, 1,110; ^1^H NMR (600 MHz, DMSO-*d*_6_): *δ* 12.21 (s, 1H), 9.21 (s, 1H), 8.87 (d, *J* = 4.4 Hz, 1H), 8.51 (d, *J* = 9.0 Hz, 1H), 7.80 (d, *J* = 2.5 Hz, 1H), 7.74 (dd, *J* = 7.8, 1.4 Hz, 1H), 7.59 (dd, *J* = 9.0, 2.5 Hz, 1H), 7.53–7.47 (m, 1H), 7.45 (t, *J* = 8.2 Hz, 1H), 7.40–7.34 (m, 3H), 7.18 (d, *J* = 7.7 Hz, 1H), 7.13–7.08 (m, 1H), 2.73 (d, *J* = 4.5 Hz, 3H); ^13^C NMR (150 MHz, DMSO-*d*_6_): *δ* 167.85, 166.83, 143.96, 142.58, 138.16, 133.12, 131.95, 130.76, 130.59, 129.34, 128.16, 127.20, 125.52, 123.51, 123.16, 122.80, 121.83, 121.57, 118.98, 117.43, 114.48, 26.71; MS (ESI) *m/z* 470.32 (M + Na)^+^, calcd. for C_22_H_17_ClF_3_N_3_O_2_ m/z = 447.10.

N-(2-Carbamoyl-3-chlorophenyl)-2-((3-(trifluoromethyl)phenyl)amino)benzamide **B11**. Yield: 62%; m.p. 194–195°C; FTIR υ_max_ (cm^−1^): 3,325, 3,171, 1,678, 1,581, 1,510, 1,326, 1,162, 1,111; ^1^H NMR (600 MHz, DMSO-*d*_6_): *δ* 10.05 (s, 1H), 9.15 (s, 1H), 7.98 (s, 1H), 7.86 (s, 1H), 7.74 (dd, *J* = 7.8, 1.2 Hz, 1H), 7.70 (d, *J* = 8.0 Hz, 1H), 7.52–7.44 (m, 2H), 7.43–7.32 (m, 5H), 7.21 (d, *J* = 7.7 Hz, 1H), 7.10–7.04 (m, 1H); ^13^C NMR (150 MHz, DMSO-*d*_6_): *δ* 167.26, 166.87, 144.02, 142.67, 136.49, 133.06, 132.01, 130.84, 130.62, 130.39, 129.82, 126.67, 125.52, 124.24, 123.72, 123.04, 122.01, 121.36, 118.75, 117.59, 114.55; MS (ESI) *m/z* 456.27 (M + Na)^+^, calcd. for C_21_H_15_ClF_3_N_3_O_2_ m/z = 433.08.

2-Chloro-N-methyl-6-(2-((3-(trifluoromethyl)phenyl)amino)benzamido)benzamide **B12**. Yield: 73%; m.p. 178–179°C; FTIR υ_max_ (cm^−1^): 3,273, 1,667, 1,641, 1,505, 1,331, 1,171, 1,108; ^1^H NMR (600 MHz, DMSO-*d*_6_): *δ* 10.14 (s, 1H), 8.95 (s, 1H), 8.44 (d, *J* = 4.6 Hz, 1H), 7.79 (d, *J* = 8.2 Hz, 1H), 7.73 (d, *J* = 7.8 Hz, 1H), 7.50–7.45 (m, 2H), 7.42 (t, *J* = 8.1 Hz, 1H), 7.36–7.33 (m, 4H), 7.20 (d, *J* = 7.7 Hz, 1H), 7.14–7.09 (m, 1H), 2.61 (d, *J* = 4.6 Hz, 3H); ^13^C NMR (150 MHz, DMSO-*d*_6_): *δ* 167.03, 165.25, 144.40, 142.27, 136.92, 132.94, 131.52, 130.79, 130.68, 130.59, 130.08, 126.46, 125.54, 124.25, 123.82, 123.74, 121.93, 121.75, 119.47, 117.39, 114.41, 26.26; MS (ESI) *m/z* 470.32 (M + Na)^+^, calcd. for C_22_H_17_ClF_3_N_3_O_2_ m/z = 447.10.

3,5-Dichloro-N- methyl-2-(2-((3-(trifluoromethyl)phenyl)amino)benzamido)benzamide **B13**. Yield: 76%; m.p. 180–181°C; FTIR υ_max_ (cm^−1^): 3,287, 1,632, 1,582, 1,524, 1,328, 1,162, 1,115; ^1^H NMR (600 MHz, CDCl_3_): *δ* 9.44 (s, 1H), 9.21 (s, 1H), 7.80 (d, *J* = 7.8 Hz, 1H), 7.57 (d, *J* = 2.2 Hz, 1H), 7.44 (s, 1H), 7.40 (dd, *J* = 5.7, 2.2 Hz, 4H), 7.35 (d, *J* = 8.3 Hz, 1H), 7.23 (d, *J* = 7.5 Hz, 1H), 6.95–6.91 (m, 1H), 6.29 (s, 1H), 2.90 (d, *J* = 4.0 Hz, 3H); ^13^C NMR (150 MHz, CDCl_3_): *δ* 168.25, 167.11, 145.16, 141.99, 134.03, 133.46, 133.28, 132.35, 132.00, 131.93, 131.69, 129.88, 129.07, 126.04, 123.19, 119.47, 118.79, 117.66, 116.75, 115.71, 26.97; MS (ESI) *m/z* 504.25 (M + Na)^+^, calcd. for C_22_H_16_Cl_2_F_3_N_3_O_2_ m/z = 481.06.

### 4.3 General Synthetic Procedures for Diarylamine-Guided Carboxamide Derivatives C1-11

To a solution of diarylamine carboxylic acid **1** (1 mmol) in 8 ml anhydrous acetonitrile was added pyridine (3 mmol), and then the reaction mixture was cooled to 0 C. Whereafter, methanesulfonyl chloride (1.5 mmol) was added dropwise to the reaction mixture over 15–20 min. After addition, the reaction mixture was then allowed to warm to room temperature and stirred for additional hours, the corresponding multi-substituted amines **5** (1.05 mmol) was added, and the reaction mixture was heated to 40–45°C and detected by thin-layer chromatography. After completion of the reaction, the mixture was quenched by the addition of water and was stirred for 20 min. The suspended solid was collected by filtration and washed with water to afford the crude products, which can be purified by silica gel column chromatography (petroleum ether/ethyl acetate) or recrystallization (methanol) to give the target molecules. Their physico-chemical properties and the spectra data are as follows:

N-(4-Isopropylphenyl)-2-((3-(trifluoromethyl)phenyl) amino)benzamide **C1**. Yield: 72%; m.p. 74–75°C; FTIR υ_max_ (cm^−1^): 3,274, 1,633, 1,595, 1,514, 1,323, 1,113; ^1^H NMR (600 MHz, CDCl_3_): *δ* 9.30 (s, 1H), 7.73 (s, 1H), 7.53 (d, *J* = 7.2 Hz, 1H), 7.41–7.25 (m, 7H), 7.18–7.10 (m, 2H), 6.99 (m, 1H), 6.85 (t, *J* = 7.2 Hz, 1H), 2.86–2.82 (m, 1H), 1.18 (d, *J* = 6.6 Hz, 6H); ^13^C NMR (150 MHz, CDCl_3_): *δ* 167.63, 145.89, 144.52, 142.17, 134.97, 132.72, 129.86, 127.61, 127.22, 127.11, 122.93, 121.01, 119.70, 119.32, 118.57, 116.27, 116.15, 33.70, 24.03; MS (ESI) *m/z* 399.55 (M + H)^+^, calcd. for C_23_H_21_F_3_N_2_O m/z = 398.16.

N-(4-Isopropylphenyl)-2-((3-(trifluoromethyl) phenyl)amino)nicotinamide **C2**. Yield: 76%; m.p. 161–162°C; FTIR υ_max_ (cm^−1^): 3,318, 1,634, 1,588, 1,507, 1,443, 1,331, 1,114; ^1^H NMR (600 MHz, DMSO-*d*_6_): *δ* 10.98 (s, 1H), 9.36 (bs, 1H), 8.43–7.34 (m, 7H), 7.18 (d, *J* = 7.2 Hz, 2H), 6.82 (s, 1H), 2.87–2.82 (m, 1H), 1.19 (d, *J* = 6.6 Hz, 6H); ^13^C NMR (150 MHz, CD_3_OD): *δ* 168.58, 156.04, 151.88, 146.91, 142.49, 138.54, 137.18, 132.19, 131.98, 130.51, 127.74, 124.07, 122.84, 119.20, 119.18, 117.07, 115.44, 114.14, 35.02, 24.52; MS (ESI) *m/z* 400.63 (M + H)^+^, calcd. for C_22_H_20_F_3_N_3_O m/z = 399.16.

N-(2-(Trifluoromethyl)phenyl)-2-((3-(trifluoromethyl) phenyl)amino)benzamide **C3**. Yield: 78%; m.p. 82–83°C; FTIR υ_max_ (cm^−1^): 3,286, 1,636, 1,580, 1,518, 1,314, 1,100; ^1^H NMR (600 MHz, CDCl_3_): *δ* 9.33 (s, 1H), 8.20 (d, *J* = 8.4 Hz, 1H), 8.16 (s, 1H), 7.62–7.52 (m, 3H), 7.39 (s, 1H), 7.35–7.29 (m, 4H), 7.23 (t, *J* = 7.2 Hz, 1H), 7.17 (s, 1H), 6.89–6.87 (m, 1H); ^13^C NMR (150 MHz, CDCl_3_): *δ* 167.51, 145.15, 141.95, 135.04, 133.33, 132.94, 129.91, 127.48, 126.34, 126.30, 125.01, 124.85, 123.34, 119.57, 118.95, 118.53, 116.82, 116.79, 116.22; MS (ESI) *m/z* 425.47 (M + H)^+^, calcd. for C_21_H_14_F_6_N_2_O m/z = 424.10.

N-(2-(Trifluoromethyl)phenyl)-2-((3-(trifluoromethyl) phenyl)amino)nicotinamide **C4**. Yield: 62%; m.p. 139–140°C; FTIR υ_max_ (cm^−1^): 3,292, 1,638, 1,599, 1,517, 1,442, 1,330, 1,109; ^1^H NMR (400 MHz, DMSO-*d*_6_): *δ* 10.79 (s, 1H), 10.54 (s, 1H), 8.47 (dd, *J* = 7.2 Hz, 1H), 8.34 (dd, *J* = 7.2 Hz, 1H), 8.28 (s, 1H), 7.86–7.77 (m, 3H), 7.61 (q, *J* = 12 Hz, 2H), 7.52 (t, *J* = 12 Hz, 1H), 7.31 (d, *J* = 12 Hz, 1H), 7.06 (q, *J* = 12 Hz, 1H); ^13^C NMR (100 MHz, DMSO-*d*_6_) *δ* 168.14, 154.73, 151.69, 141.26, 138.26, 135.49, 133.76, 132.01, 130.26, 130.14, 129.83, 128.47, 127.09, 127.04, 126.09, 125.41, 123.49, 122.69, 118.32, 115.69, 115.65, 115.03, 111.66; MS (ESI) *m/z* 426.55 (M + H)^+^, calcd. for C_20_H_13_F_6_N_3_O m/z = 425.10.

N-(3-(Trifluoromethyl)phenyl)-2-((3-(trifluoromethyl) phenyl)amino)benzamide **C5**. Yield: 70%; m.p. 75–76°C; FTIR υ_max_ (cm^−1^): 3,292, 1,638, 1,519, 1,443, 1,325, 1,154, 1,118; ^1^H NMR (600 MHz, CDCl_3_): *δ* 9.19 (s, 1H), 7.91 (s, 1H), 7.84 (s, 1H), 7.69 (d, *J* = 7.8 Hz, 1H), 7.54 (d, *J* = 7.8 Hz, 1H), 7.43 (t, *J* = 7.8 Hz, 1H), 7.37–7.26 (m, 6H), 7.18–7.16 (m, 1H), 6.87–6.85 (m, 1H); ^13^C NMR (150 MHz, CDCl_3_): *δ* 167.66, 144.83, 141.95, 138.07, 133.22, 131.90, 131.70, 129.94, 129.71, 127.61, 124.89, 124.71, 123.59, 123.31, 121.41, 119.41, 118.93, 117.32, 116.62, 116.36; MS (ESI) *m/z* 425.56 (M + H)^+^, calcd. for C_21_H_14_F_6_N_2_O m/z = 424.10.

N-(3-(Trifluoromethyl)phenyl)-2-((3-(trifluoromethyl) phenyl)amino)nicotinamide **C6**. Yield: 73%; m.p. 130–131°C; FTIR υ_max_ (cm^−1^): 3,293, 1,638, 1,599, 1,517, 1,462, 1,330, 1,156, 1,109; ^1^H NMR (600 MHz, CDCl_3_): *δ* 10.35 (s, 1H), 8.36 (t, *J* = 4.8 Hz, 1H), 8.02 (s, 1H), 7.90 (s, 1H), 7.83–7.71 (m, 4H), 7.48–7.34 (m, 3H), 7.21 (d, *J* = 7.8 Hz, 1H), 6.78 (s, 1H); ^13^C NMR (150 MHz, CDCl_3_): *δ* 166.48, 155.14, 153.22, 152.20, 140.22, 137.68, 135.68, 131.61, 129.83, 129.57, 129.23, 123.93, 123.46, 121.86, 120.70, 119.08, 117.63, 117.09, 113.86, 110.94; MS (ESI) *m/z* 426.55 (M + H)^+^, calcd. for C_20_H_13_F_6_N_3_O m/z = 425.10.

N-(4-(Trifluoromethyl)phenyl)-2-((3-(trifluoromethyl) phenyl)amino)benzamide **C7**. Yield: 76%; m.p. 102–103°C; FTIR υ_max_ (cm^−1^): 3,332, 1,644, 1,594, 1,505, 1,325, 1,157, 1,108; ^1^H NMR (600 MHz, CDCl_3_): *δ* 9.18 (s, 1H), 7.94 (s, 1H), 7.65–7.54 (m, 5H), 7.37–7.32 (m, 4H), 7.27 (d, *J* = 8.4 Hz, 1H), 7.18 (d, *J* = 7.8 Hz, 1H), 6.88–6.85 (m, 1H); ^13^C NMR (150 MHz, CDCl_3_): *δ* 167.67, 144.86, 141.93, 140.63, 133.29, 131.92, 131.71, 129.96, 127.64, 126.44, 126.42, 123.29, 120.11, 119.43, 119.00, 118.98, 118.93, 116.63, 116.60, 116.38; MS (ESI) *m/z* 425.65 (M + H)^+^, calcd. for C_21_H_14_F_6_N_2_O m/z = 424.10.

N-(4-(Trifluoromethyl)phenyl)-2-((3-(trifluoromethyl) phenyl)amino)nicotinamide **C8**. Yield: 73%; m.p. 171–172°C; FTIR υ_max_ (cm^−1^): 3,319, 1,645, 1,585, 1,508, 1,442, 1,309, 1,119; ^1^H NMR (600 MHz, DMSO-*d*_6_): ^1^H NMR (600 MHz, CDCl_3_): *δ* 10.81 (s, 1H), 10.30 (s, 1H), 8.43 (dd, *J* = 4.2 Hz, 1H), 8.29–8.25 (m, 2H), 7.98 (d, *J* = 8.4 Hz, 2H), 7.87 (d, *J* = 8.4 Hz, 1H), 7.76 (d, *J* = 8.4 Hz, 2H), 7.52 (t, *J* = 8.4 Hz, 1H), 7.31 (d, *J* = 7.2 Hz, 1H), 7.04 (dd, *J* = 4.8 Hz, 1H); ^13^C NMR (150 MHz, DMSO-*d*_6_): *δ* 153.68, 151.36, 145.14, 141.78, 137.92, 130.20, 126.92, 124.13, 121.28, 118.98, 115.87, 115.00, 114.14; MS (ESI) *m/z* 426.55 (M + H)^+^, calcd. for C_20_H_13_F_6_N_3_O m/z = 425.10.

N-(Pyridin-2-yl)-2-((3-(trifluoromethyl)phenyl)amino)benzamide **C9**. Yield: 55%; m.p. 65–66°C; FTIR υ_max_ (cm^−1^): 2,958, 1,663, 1,575, 1,493, 1,435, 1,340, 1,298, 1,109; ^1^H NMR (600 MHz, CDCl_3_): *δ* 9.40 (s, 1H), 9.25 (br, 1H), 8.30 (d, *J* = 8.4Hz, 1H), 8.19 (d, *J* = 4.2Hz, 1H), 7.76–7.68 (m, 3H), 7.47–7.31 (m, 3H), 7.18 (t, *J* = 6.6 Hz, 1H), 7.04 (t, *J* = 6.6 Hz, 1H), 6.85–6.83 (m, 1H); ^13^C NMR (150 MHz, CDCl_3_): *δ* 167.92, 151.22, 146.76, 145.20, 141.96, 139.37, 133.39, 131.91, 131.70, 129.90, 128.35, 123.39, 119.97, 119.32, 118.96, 118.23, 116.93, 116.01, 114.72; MS (ESI) *m/z* 358.60 (M + H)^+^, calcd. for C_19_H_14_F_3_N_3_O m/z = 357.11.

N-(2-Chloropyridin-4-yl)-2-((3-(trifluoromethyl)phenyl)amino)benzamide **C10**. Yield: 68%; m.p. 97–98°C; FTIR υ_max_ (cm^−1^): 2,959, 1,663, 1,575, 1,525, 1,493, 1,435, 1,340, 1,298, 1,109; ^1^H NMR (600 MHz, CDCl_3_): *δ* 9.19 (s, 1H), 8.31 (d, *J* = 5.4Hz, 1H), 8.17 (s, 1H), 7.76 (d, *J* = 1.8 Hz, 1H), 7.60 (d, J = 7.8Hz, 1H), 7.45–7.35 (m, 6H), 7.28 (d, *J* = 7.2 Hz, 1H), 6.93 (t, *J* = 7.8 Hz, 1H); ^13^C NMR (150 MHz, CDCl_3_): *δ* 167.76, 152.64, 150.31, 146.93, 145.29, 141.70, 133.84, 130.04, 127.71, 123.69, 119.44, 118.00, 117.00, 116.53, 113.86, 112.79; MS (ESI) *m/z* 392.26 (M + H)^+^, calcd. for C_19_H_13_ClF_3_N_3_O m/z = 391.07.

N-(6-Chloropyridin-3-yl)-2-((3-(trifluoromethyl)phenyl)amino)nicotinamide **C11**. Yield: 74%; m.p. 166–167°C; FTIR υ_max_ (cm^−1^): 3,316, 1,643, 1,605, 1,581, 1,505, 1,443, 1,282, 1,110; ^1^H NMR (600 MHz, DMSO-*d*_6_): *δ* 10.81 (s, 1H), 10.36 (s, 1H), 8.76 (d, *J* = 2.7 Hz, 1H), 8.44 (dd, *J* = 4.8, 1.8 Hz, 1H), 8.29–8.21 (m, 3H), 7.87 (dd, *J* = 8.2, 1.4 Hz, 1H), 7.57–7.51 (m, 2H), 7.31 (d, *J* = 7.7 Hz, 1H), 7.04 (dd, *J* = 7.7, 4.8 Hz, 1H); ^13^C NMR (150 MHz, DMSO-*d*_6_): *δ* 167.22, 154.25, 151.49, 144.99, 142.61, 141.34, 138.63, 135.46, 132.13, 130.00, 125.67, 124.59, 123.86, 123.70, 118.38, 115.91, 114.97, 113.07; MS (ESI) *m/z* 393.34 (M + H)^+^, calcd. for C_18_H_12_ClF_3_N_4_O m/z = 392.07.

### 4.4 *In vitro* Cytotoxicity Assay

The cytotoxicity of target compounds was determined with MTT assay (Alley et al., 1988; Ke et al., 2012; Shi et al., 2012; Yu et al., 2015; Ke et al., 2019; Xu et al., 2019; Huang et al., 2021). SGC-7901, A875, HepG2 and MARC145 cell lines were obtained from Hubei Biopesticide Engineering Research Centre. All data of the experiment were analyzed with SPSS software, and all assay were performed in triplicate on three independent experiments, and measurement data were expressed as the mean ± S.D.

### 4.5 Flow Cytometric Analysis

Quantitative analysis of apoptotic and necrotic cell death induced by potential molecules was investigated using Annexin V-FITC/PI dual staining assay (Yu et al., 2015; Huang et al., 2021).

### 4.6 Measurement of Lactate Dehydrogenase

A875 cells grown in 96-well plates were treated with serial dilutions of each tested compound for 24 h. The culture media were collected, and the concentrations of LDH were determined based on our previous research (Huang et al., 2021).

### 4.7 Kinase Inhibitory Assay

The kinase inhibitory profile of compounds **C6** and **C11** were screened at two dose concentration of 10 and 1 *µ*M over a panel of 15 kinases, and each assay was repeated twice. All the inhibitory assays were carried out through kinase profiling services provided by HY Biotech (Chinese), in which ADP-GLO kinase assays were used.

## Data Availability

The original contributions presented in the study are included in the article/Supplementary Material, further inquiries can be directed to the corresponding authors.
