# Supplementary material for: Diarylamine-Guided Carboxamide Derivatives: Synthesis, Biological Evaluation, and Potential Mechanism of Action
Source: Front Chem. 2022 Jul 12;10:953523. doi: 10.3389/fchem.2022.953523 (PMC9315260; doi:10.3389/fchem.2022.953523)
Supplement: Supplementary file 1 [file DataSheet1.docx]

**Diarylamine-guided carboxamide derivatives: Synthesis, biological evaluation and potential mechanism of action**

**Shaoyong Ke***, **Wenbo Huang, Zhigang Zhang, Yueying Wang, Yani Zhang, Zhaoyuan Wu, Wei Fang, Zhongyi Wan, Yan Gong, Jingzhong Yang, Kaimei Wang***, **Liqiao Shi***

*Key Laboratory of Microbial Pesticides, Ministry of Agriculture and Rural Affairs, National Biopesticide Engineering Research Centre, Hubei Biopesticide Engineering Research Centre, Hubei Academy of Agricultural Sciences, Wuhan 430064, People’s Republic of China*

**Address correspondence: Hubei Biopesticide Engineering Research Centre, Hubei Academy of Agricultural Sciences, Wuhan 430064, People′s Republic of China; Tel: +86-27-59101919; E-mail: shaoyong.ke@nberc.com (S. Ke), kaimei.wang@nberc.com (K. Wang), liqiao.shi@nberc.com (L. Shi)*

**Supporting Information**

**Experimental section**

***Instrumentation and chemicals***

Melting points (m.p.) were measured using a digital model X-5 apparatus (Shanghai Instrument Physical Optics Instrument Co., LTD, Shanghai, China) and were uncorrected. Infrared (IR) Spectra were recorded using Shimadzu FTIR 8400S spectrophotometer. ^1^H NMR and ^13^C NMR spectra were recorded on a Bruker spectrometer at 600 MHz (Bruker, Bremen, Germany) with CDCl_3_, DMSO-*d*_6_ or CD_3_OD as the solvent. Liquid chromatography-tandem mass spectrometry (LC−MS/MS) analysis were performed on a Waters ACQUITY UPLC^®^ H-CLASS PDA (Waters^®^, Milford, MA, USA) instrument. Analytical thin-layer chromatography was carried out on precoated plates, and spots were visualized with ultraviolet light. All chemicals or reagents used for syntheses were commercially available.

***General synthesis of diamides derivatives containing diarylamine unit B1-13***

All the diarylamine-guided diamides derivatives **B1-13** were prepared according to the similar procedures described in our previous reports, and were purified by recrystallization with methanol. The structures of all target compounds were confirmed by their spectral analysis (see in Experimental), and the MS analyses for the key intermediates **3a-g** are as following:

2-(2-((3-(Trifluoromethyl)phenyl)amino)phenyl)-4*H*-benzo[*d*][1,3]oxazin-4-one **3a**. MS (ESI) *m/z* 383.42 (M+H)^+^, calcd. for C_21_H_13_F_3_N_2_O_2_ m/z = 382.09.

8-Bromo-6-fluoro-2-(2-((3-(trifluoromethyl)phenyl)amino)phenyl)-4*H*-benzo[*d*][1,3]oxazin-4-one **3b**. MS (ESI) *m/z* 479.39 (M+H)^+^, calcd. for C_21_H_11_BrF_4_N_2_O_2_ m/z = 477.99.

8-Methyl-2-(2-((3-(trifluoromethyl)phenyl)amino)phenyl)-4*H*-benzo[*d*][1,3]oxazin-4-one **3c**. MS (ESI) *m/z* 397.55 (M+H)^+^, calcd. for C_22_H_15_F_3_N_2_O_2_ m/z = 396.11.

7-Chloro-2-(2-((3-(trifluoromethyl)phenyl)amino)phenyl)-4*H*-benzo[*d*][1,3]oxazin-4-one **3d**. MS (ESI) *m/z* 417.36 (M+H)^+^, calcd. for C_21_H_12_ClF_3_N_2_O_2_ m/z = 416.05.

6-Chloro-2-(2-((3-(trifluoromethyl)phenyl)amino)phenyl)-4*H*-benzo[*d*][1,3]oxazin-4-one **3e**. MS (ESI) *m/z* 417.45 (M+H)^+^, calcd. for C_21_H_12_ClF_3_N_2_O_2_ m/z = 416.05.

5-Chloro-2-(2-((3-(trifluoromethyl)phenyl)amino)phenyl)-4*H*-benzo[*d*][1,3]oxazin-4-one **3f**. MS (ESI) *m/z* 417.36 (M+H)^+^, calcd. for C_21_H_12_ClF_3_N_2_O_2_ m/z = 416.05.

6,8-Dichloro-2-(2-((3-(trifluoromethyl)phenyl)amino)phenyl)-4*H*-benzo[*d*][1,3]oxazin-4-one **3g**. MS (ESI) *m/z* 451.39 (M+H)^+^, calcd. for C_21_H_11_Cl_2_F_3_N_2_O_2_ m/z = 450.01.

***General synthetic procedures for diarylamine-guided carboxamide derivatives C1-11***

To a solution of diarylamine carboxylic acid **1** (1 mmol) in 8 mL anhydrous acetonitrile was added pyridine (3 mmol), and then the reaction mixture was cooled to 0 ^o^C. Whereafter, methanesulfonyl chloride (1.5 mmol) was added dropwise to the reaction mixture over 15-20 minutes. After addition, the reaction mixture was then allowed to warm to room temperature and stirred for additional hours, the corresponding multi-substituted amines **5** (1.05 mmol) was added, and the reaction mixture was heated to 40-45 ^o^C and detected by thin-layer chromatography. After completion of the reaction, the mixture was quenched by the addition of water and was stirred for 20 min. The suspended solid was collected by filtration and washed with water to afford the crude products, which can be purified by silica gel column chromatography (petroleum ether/ethyl acetate) or recrystallization (methanol) to give the target molecules. Their physico-chemical properties and the spectra data are included in the experimental.

***Cell lines***

SGC-7901 (Human gastric cancer cell line), A875 (Human melanoma cell line), HepG2 (Human hepatocellular liver carcinoma cell line) and MARC145 (A subclone of African green monkey kidney cell line MA-104) cell lines were obtained from Hubei Biopesticide Engineering Research Centre.

***In vitro cytotoxicity assays***

The *in vitro* cytotoxicity of the synthesized compounds against different cell lines (SGC-7901, A875, HepG2 and MARC145) was measured with MTT assay. All the data of the experiment were analyzed with SPSS software, and the 50% inhibitory concentrations (IC_50_) of each compound for the different cell lines were determined. A control was run for each test, and all assays were performed in triplicate on three independent experiments, and measurement data were expressed as the mean ± S.D.

***Flow cytometric analysis***

Quantitative analysis of apoptotic and necrotic cell death induced by the test compounds was performed by Annexin V-FITC apoptosis detection kit according to the manufacturer’s instructions (Abcam). Briefly, 2×10^5^ A875 cells were seeded in 6-well plates and grown overnight. After removal of the growth medium, cells were treated with compounds C6 or C11 for 24 h, at concentrations corresponding to their 0.5×IC_50s_, IC_50s_ or 2×IC_50s_. Cells treated with 0.1% DMSO were served as solvent control. Following treatment cells were harvested, washed twice with ice-cold PBS and resuspended in binding buffer. Then the cells were stained by adding 5 µl of Annexin V-FITC and 5 µl of propidium iodide, sit for 5 min at room temperature in the dark and analyzed by flow cytometry (Beckman coulter FC500).

***Measurement of LDH in culture media***

A875 cells grown in 96-well plates were treated with serial dilutions of each tested compound for 24 h. The culture media were collected, and the concentrations of LDH were determined with an LDH assay kit according to the manufacturer’s instructions (Abcam).

***Kinase inhibitory assay***

The kinase inhibitory profile of compounds **C6** and **C11** were screened at two dose concentration of 10 *µ*M and 1 *µ*M over a panel of 15 kinases, and each assay was repeated twice. All the inhibitory assays were carried out through kinase profiling services provided by HY Biotech (Chinese), in which ADP-GLO kinase assays were used.

**Table S1** Kinase inhibitory activity

| **Entry** | **Kinase** | **Inhibition activity of selective compounds** (%) | | | | |
| --- | --- | --- | --- | --- | --- | --- |
|  |  | **C6** (10 µM) | **C6** (1 µM) | **C11** (10 µM) | **C11** (1 µM) |  |
| 1 | AKT1 | -6.77 | 10.45 | -5.21 | 13.28 | |
| 2 | AKT3 | -2.65 | 16.07 | -0.49 | 20.14 | |
| 3 | CHK1 | -0.18 | 10.25 | 3.66 | 19.54 | |
| 4 | DAPK1 | -7.18 | 8.48 | 6.49 | -2.25 | |
| 5 | CDK1/A2 | -3.82 | 1.55 | -7.23 | 38.17 | |
| 6 | MAPK1 | 17.60 | 28.76 | 7.96 | 24.44 | |
| 7 | AURORA | 7.74 | 4.35 | 12.58 | 9.65 | |
| 8 | CDK2/A2 | 15.01 | 23.42 | 16.72 | 26.76 | |
| 9 | ERK1 | -6.10 | 6.76 | 3.07 | 18.22 | |
| 10 | PAK3 | 1.34 | 10.12 | 8.14 | 11.83 | |
| 11 | PLK1 | -0.95 | 5.21 | 2.02 | 7.32 | |
| 12 | PDK1 | 10.06 | 10.35 | 10.92 | 20.32 | |
| 13 | RIPK3 | 16.78 | -1.37 | 10.43 | 2.34 | |
| 14 | PIM1 | 15.84 | 12.82 | 10.30 | 23.09 | |
| 15 | CAMK2 alpha | 37.81 | 5.12 | -1.53 | 5.65 | |

***Spectroscopy for target compounds***

The structures of synthesized compounds **B1-13**, and **C1-11** were confirmed by their ^1^H NMR, ^13^C NMR and ESI-MS spectra analyses, and all the ^1^H NMR, ^13^C NMR and ESI-MS spectra analyses were in good agreement with the proposed structures. The typical ^1^H NMR, ^13^C NMR spectra for all target compounds have been presented in the following, which can further confirm the result.

**Compd. B1**


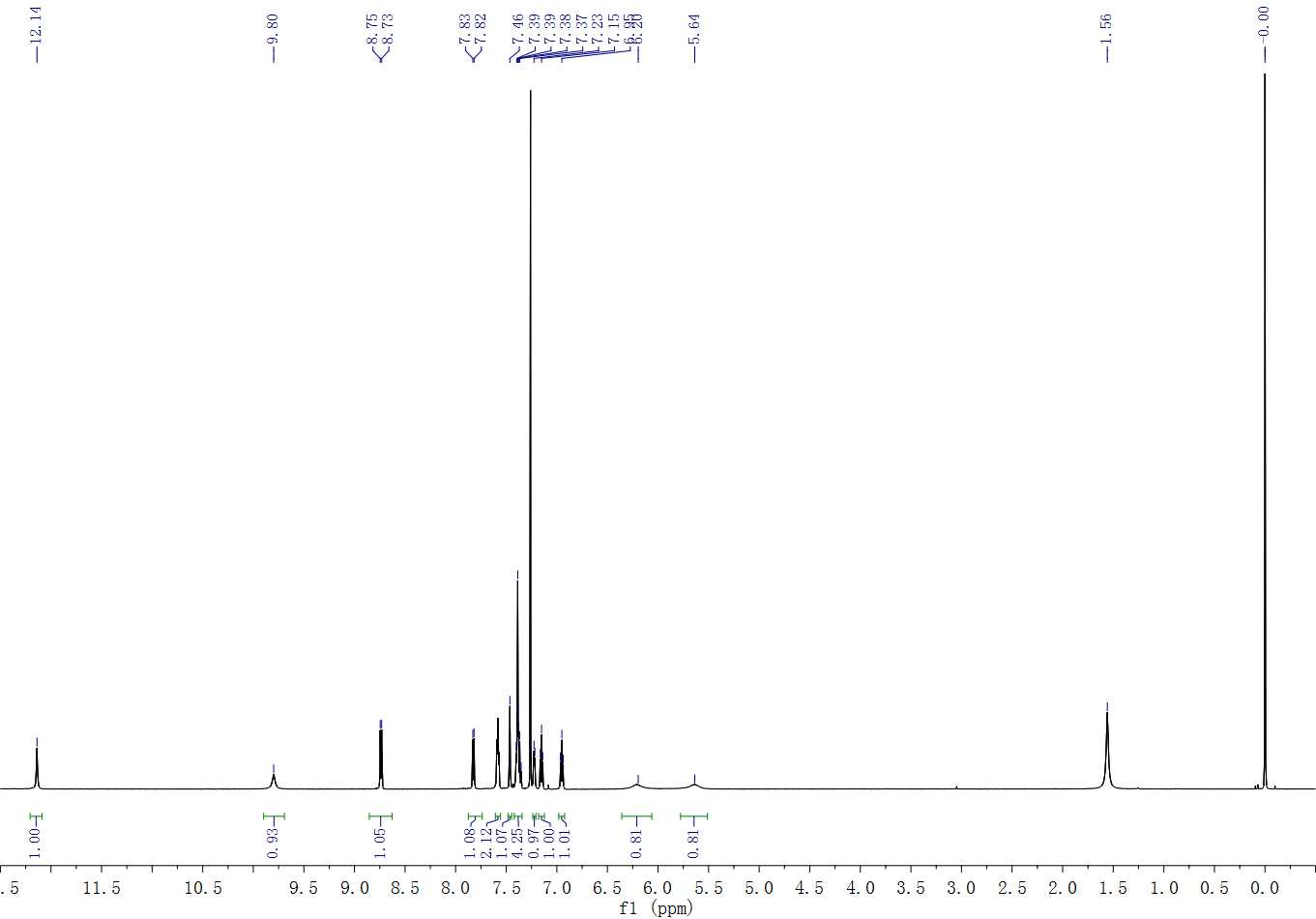


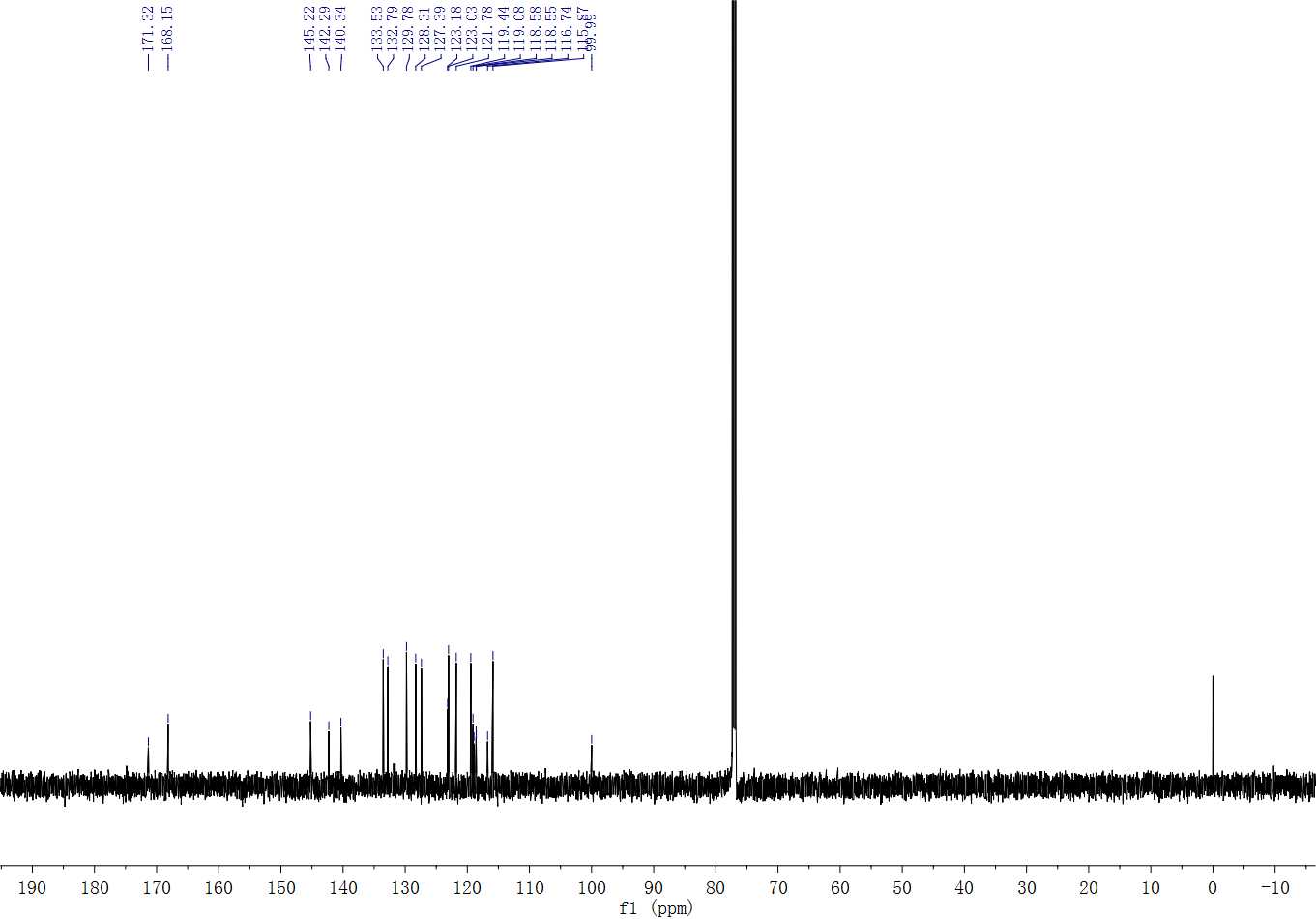


**Compd. B2**


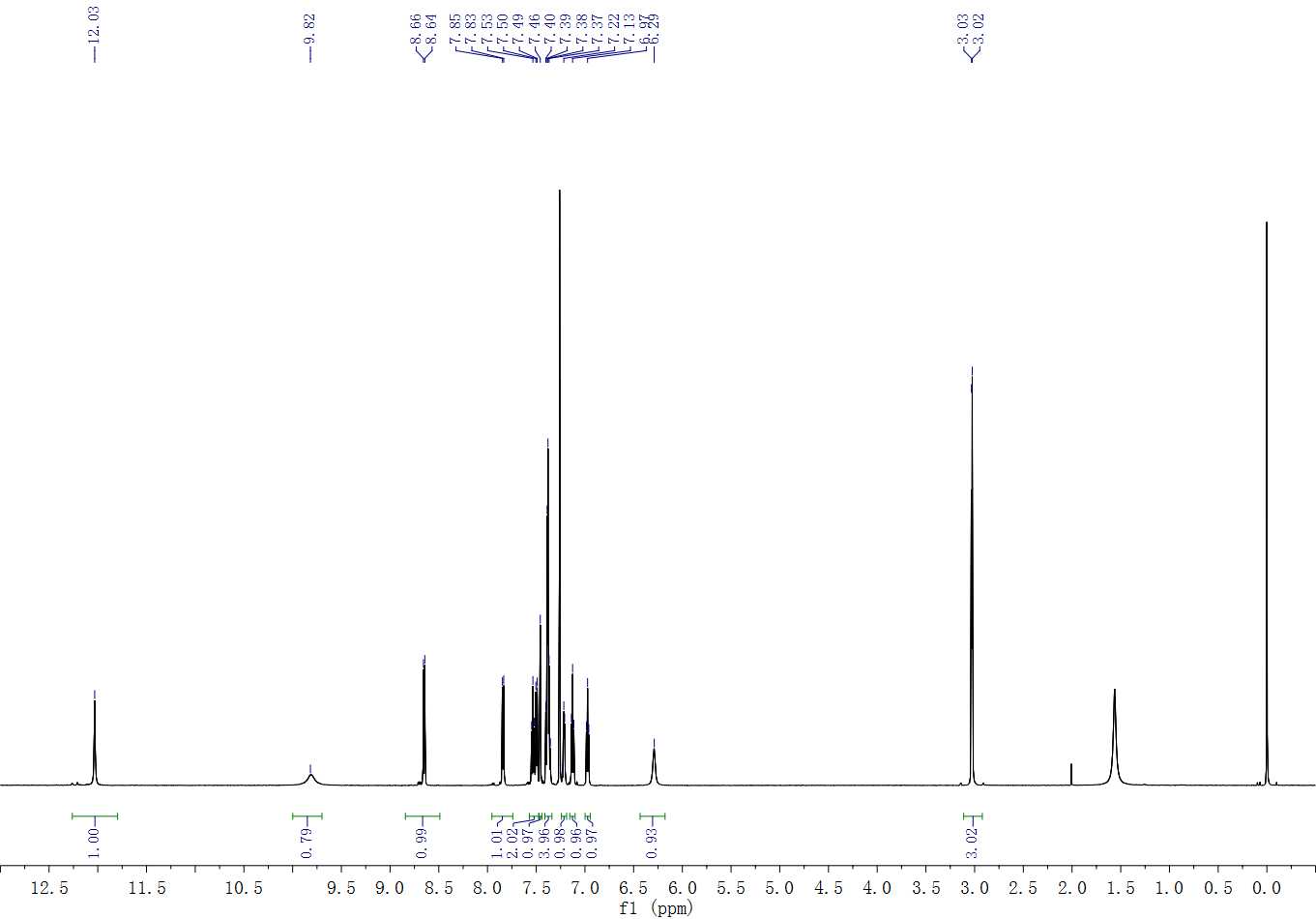

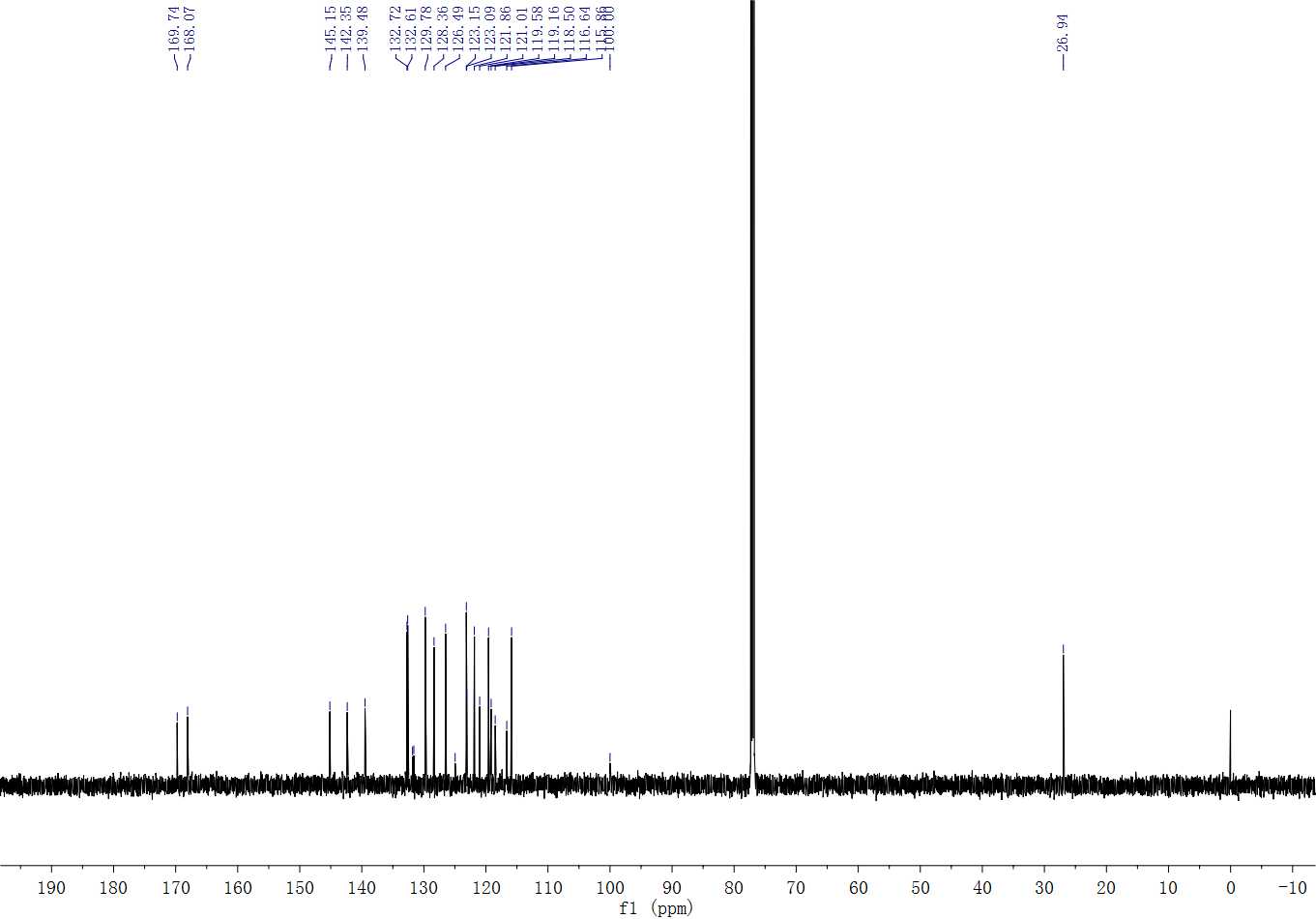


**Compd. B3**

**
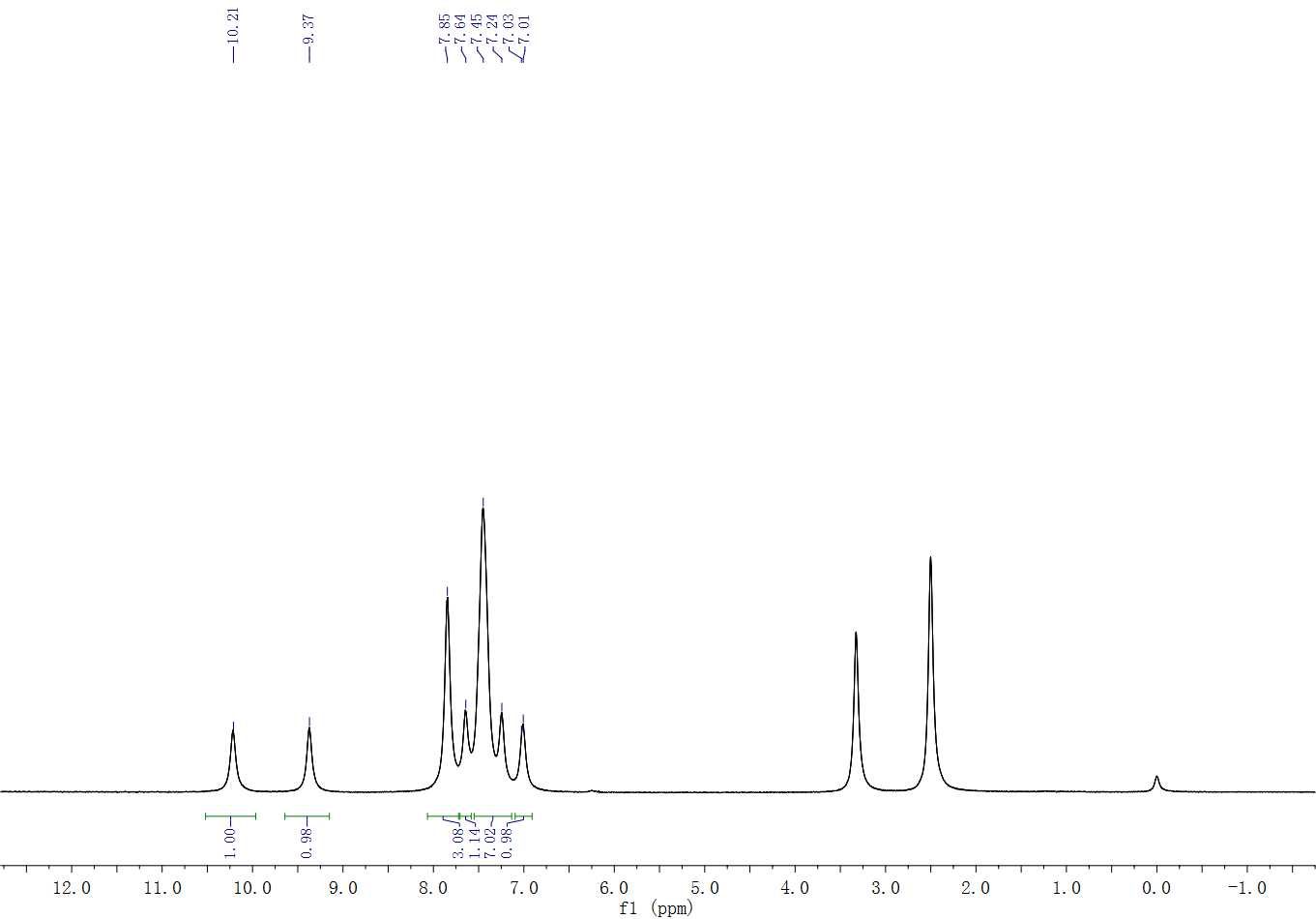

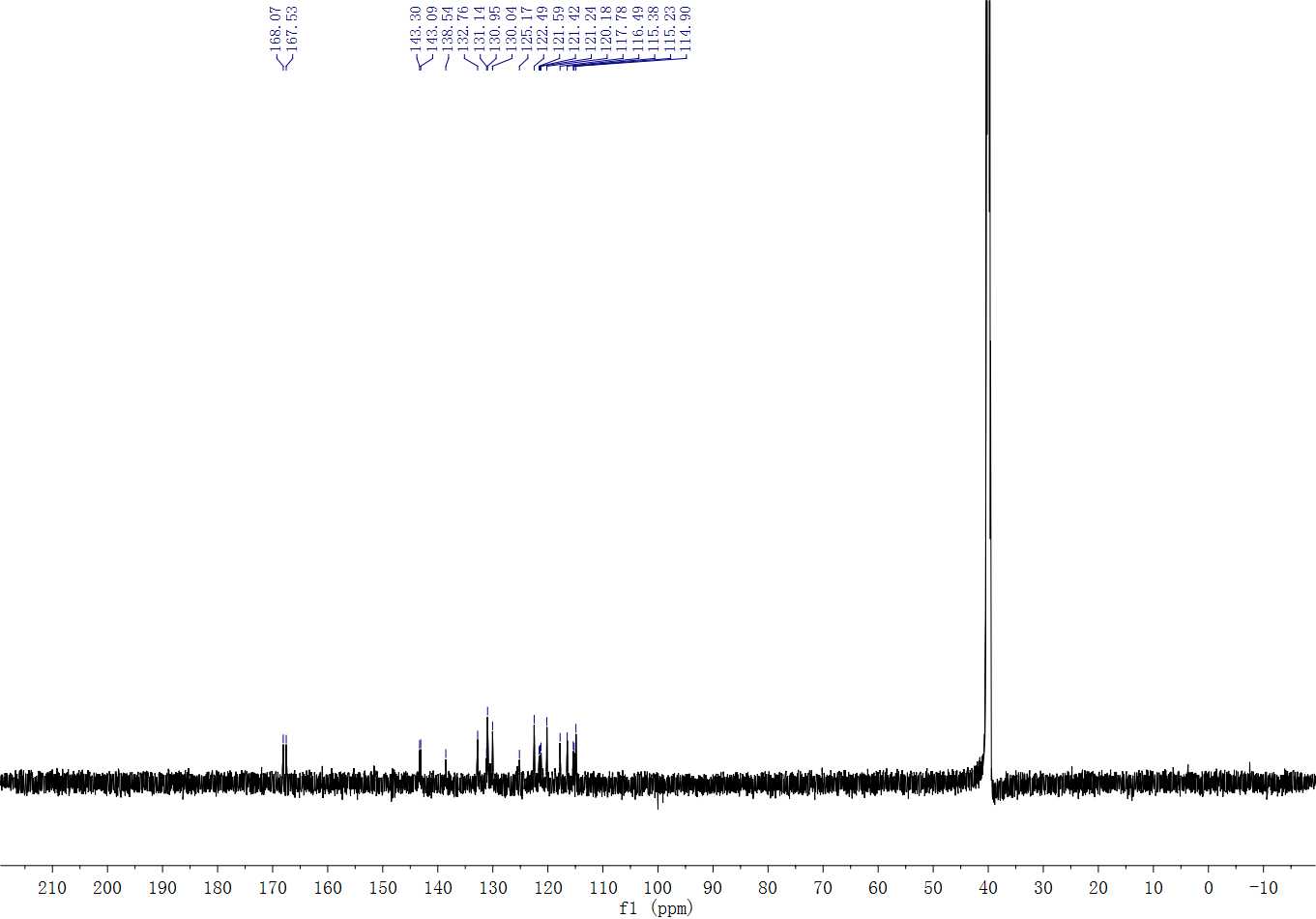
**

**Compd. B4**

**
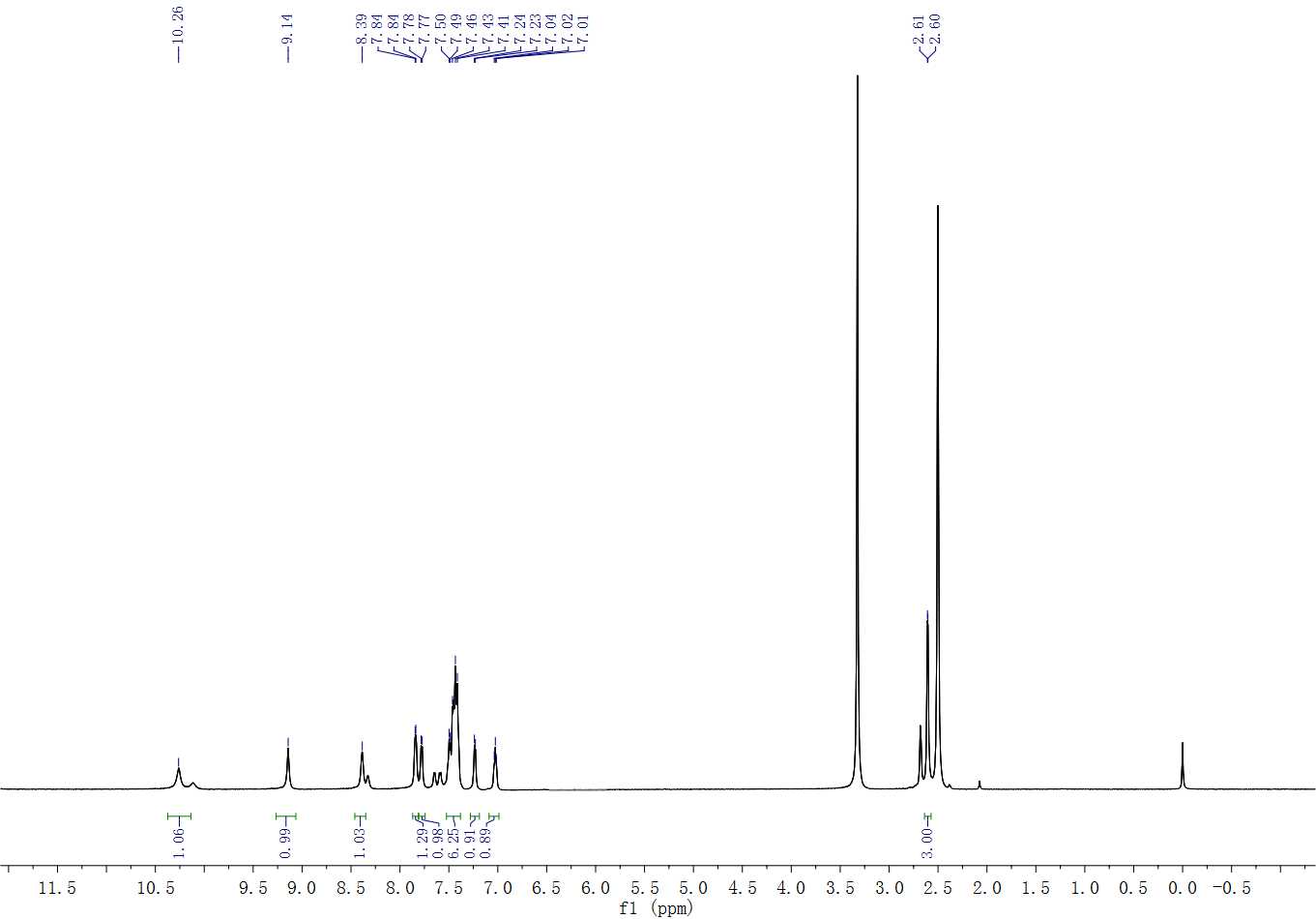

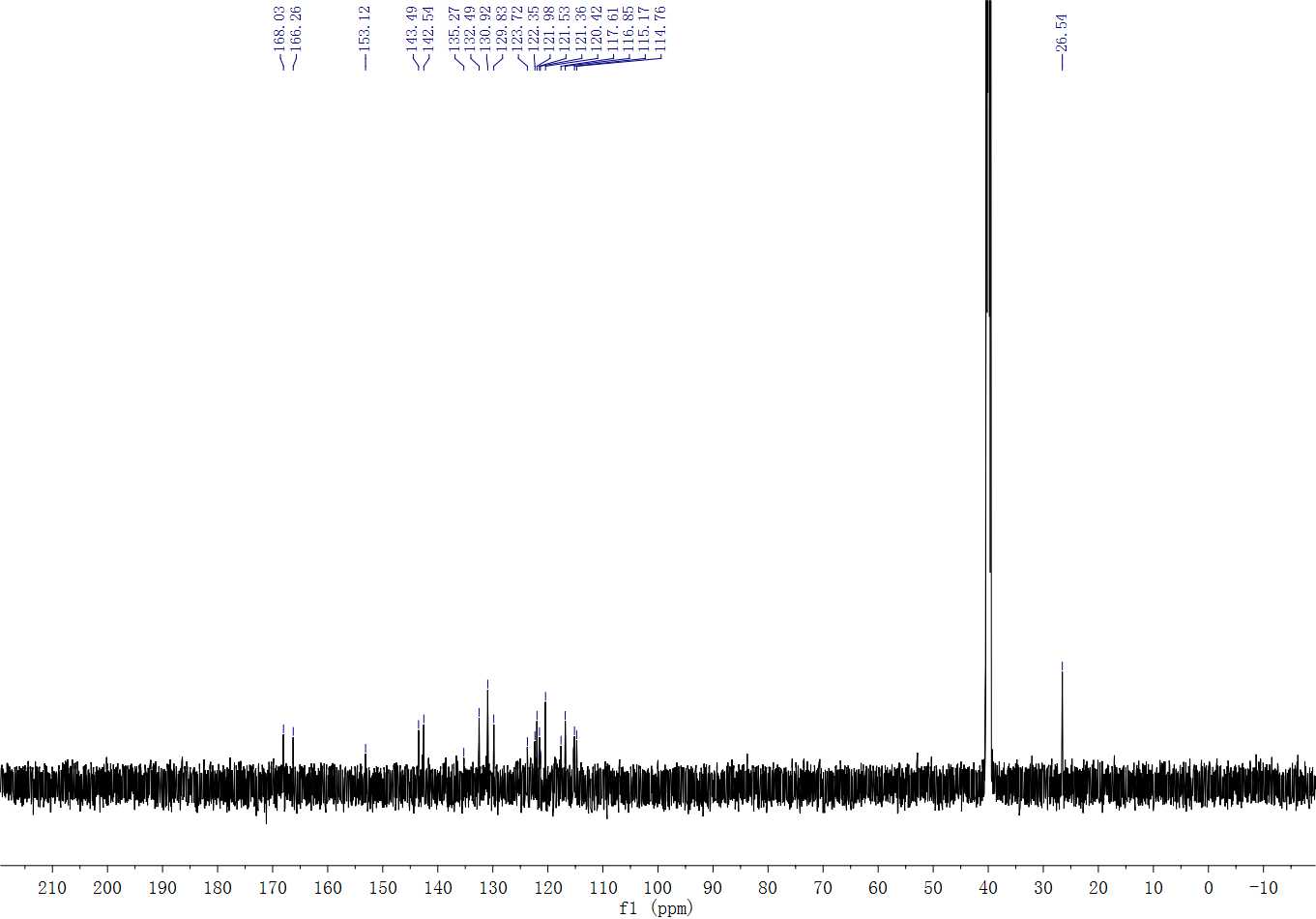
**

**Compd. B5**

**
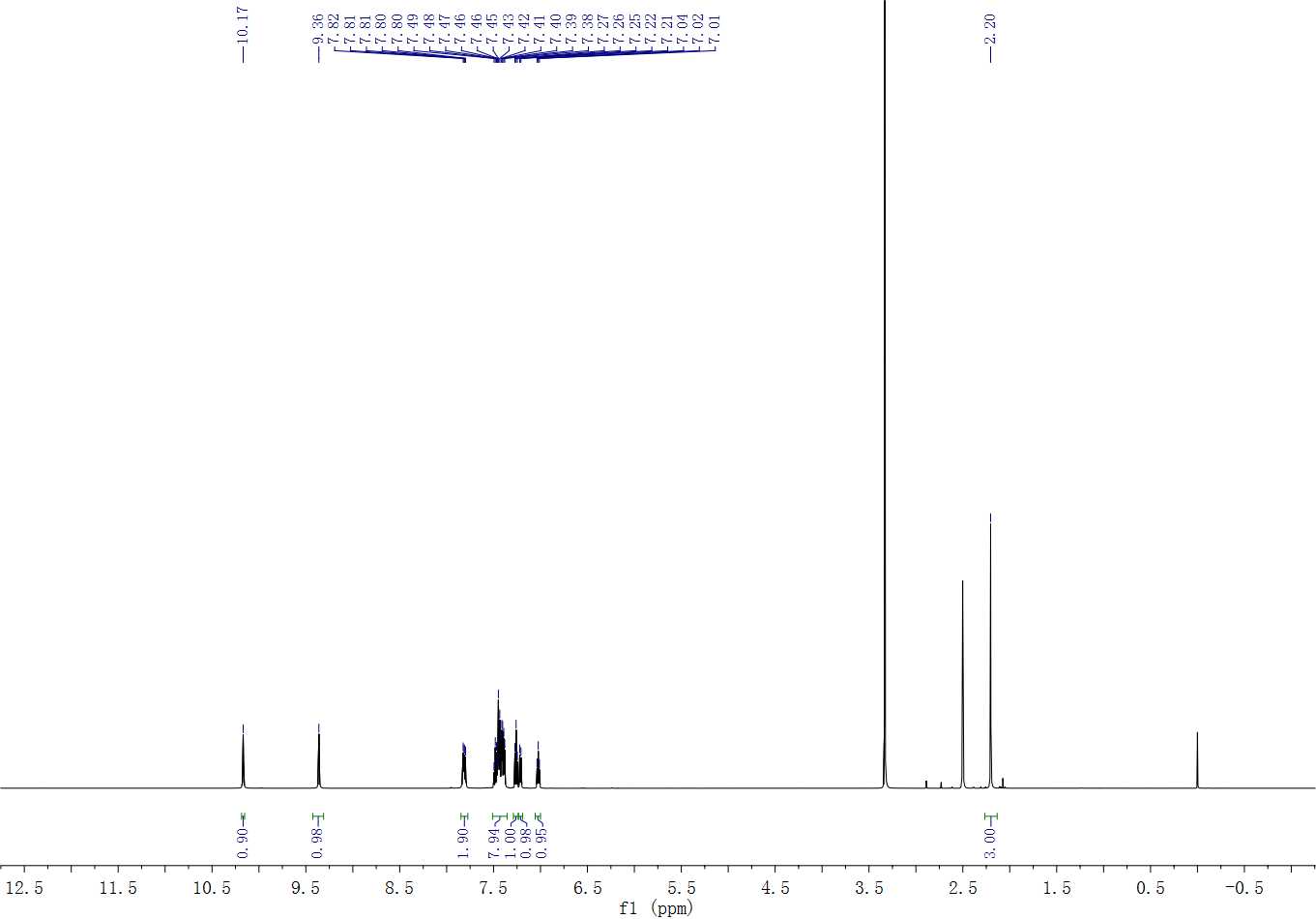

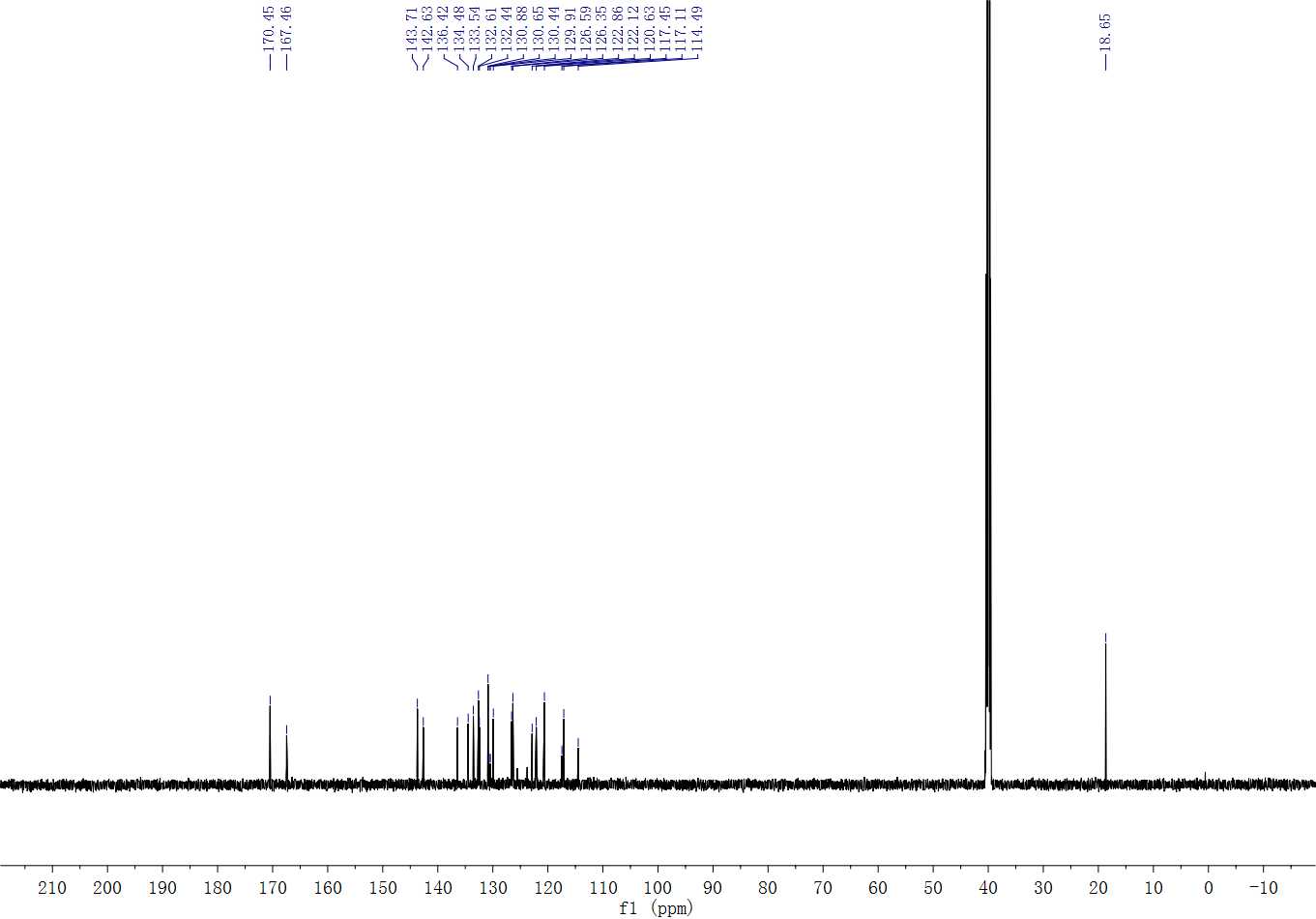
**

**Compd. B6**

**
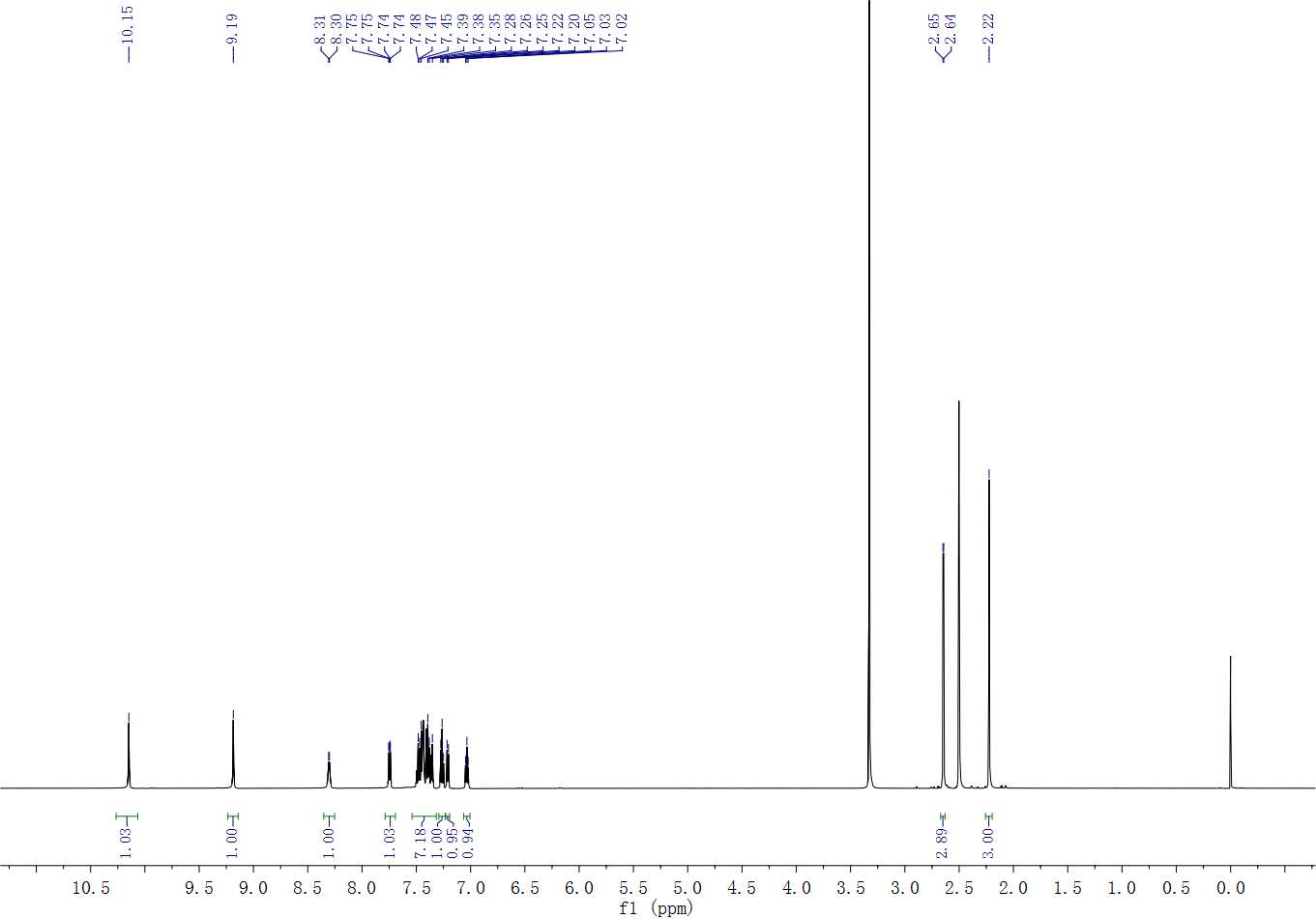

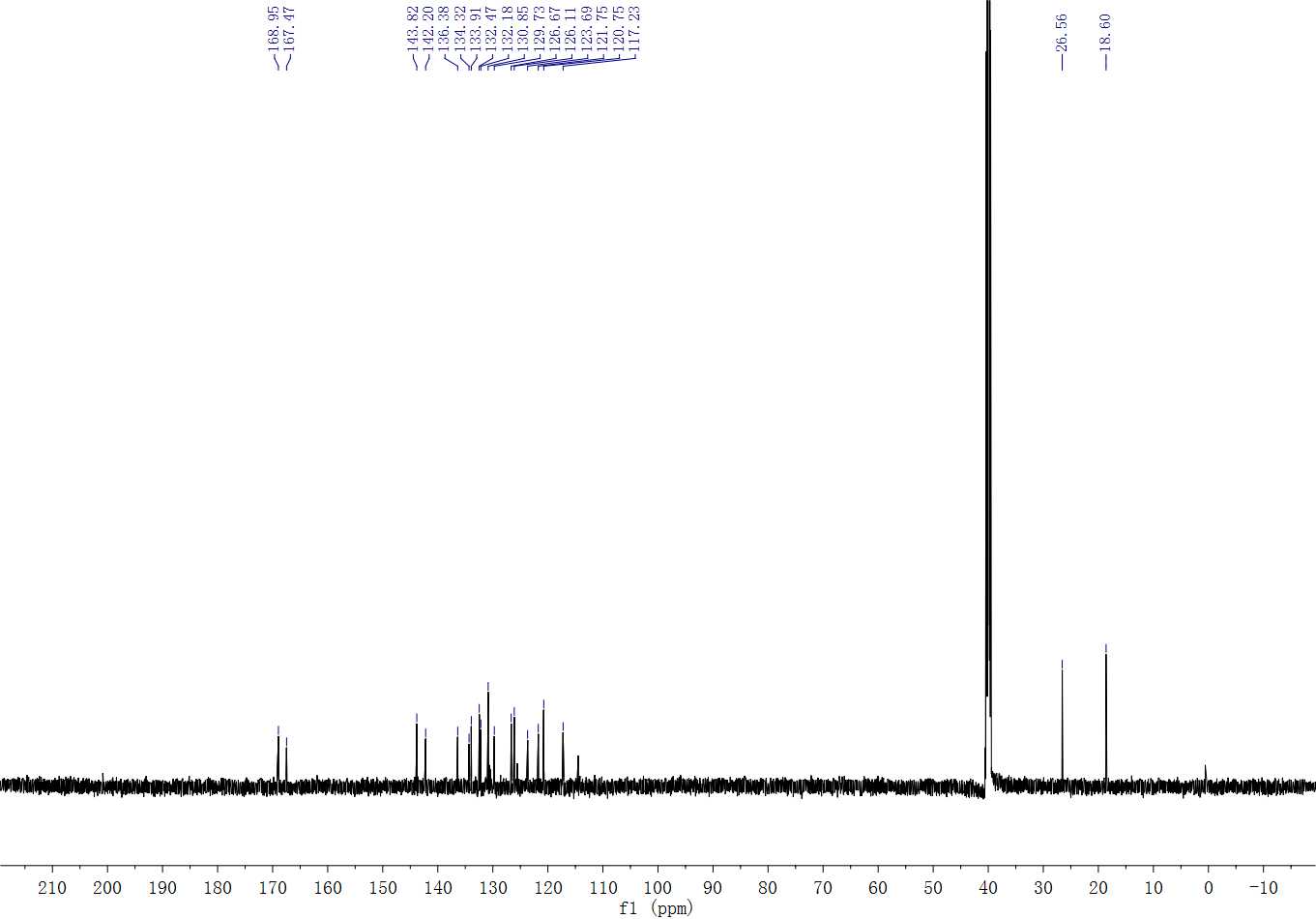
**

**Compd. B7**

**
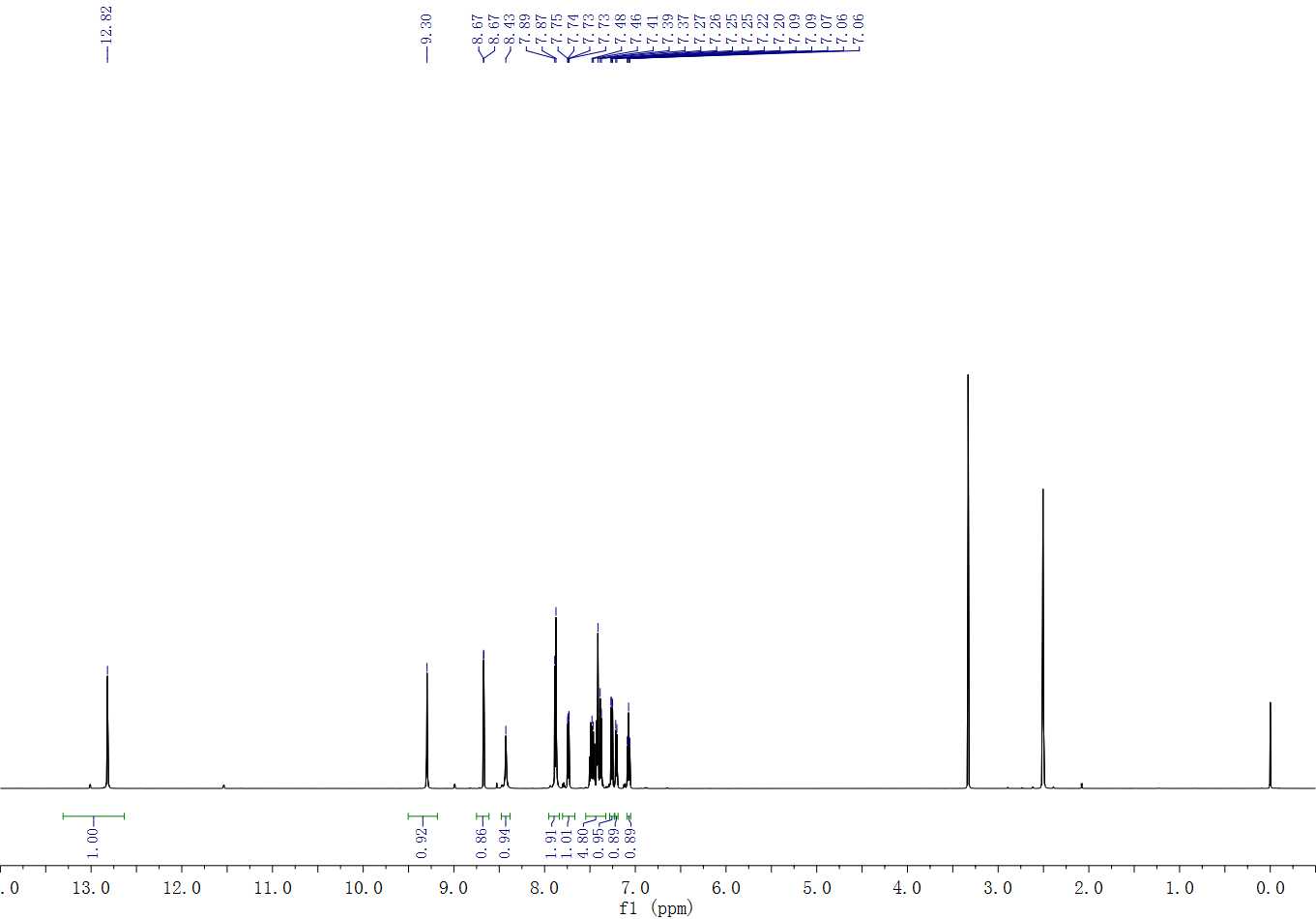

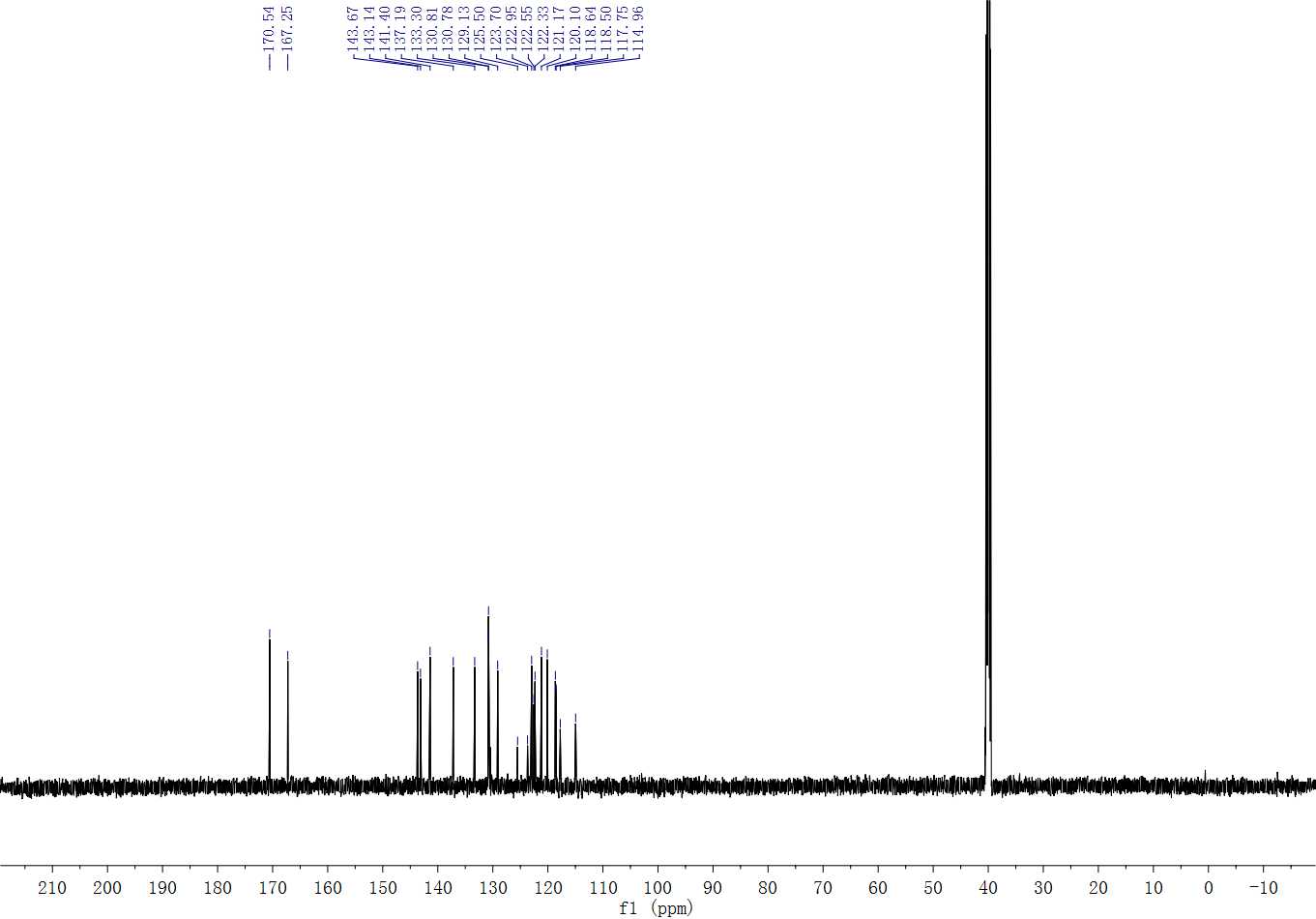
**

**Compd. B8**

**
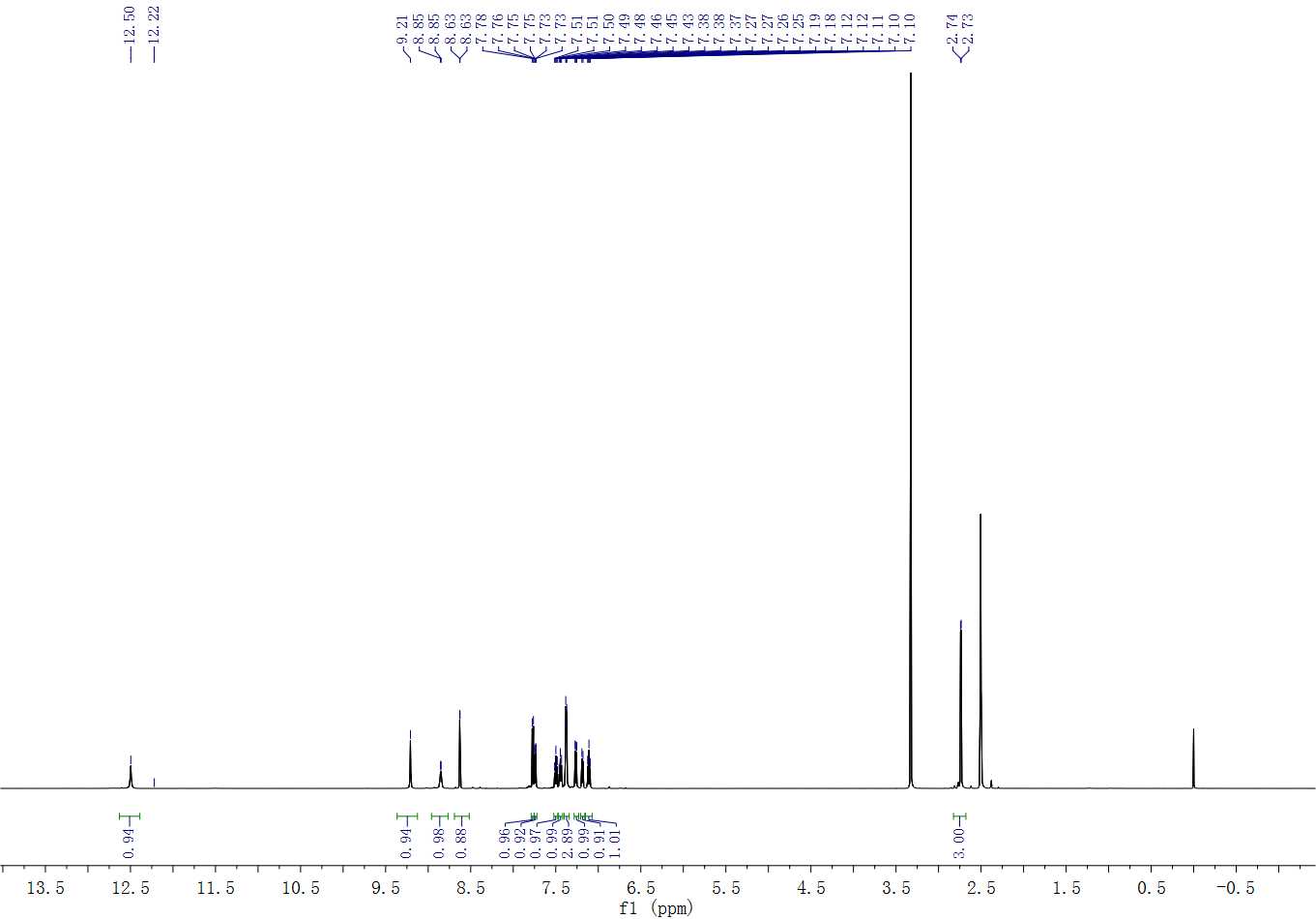

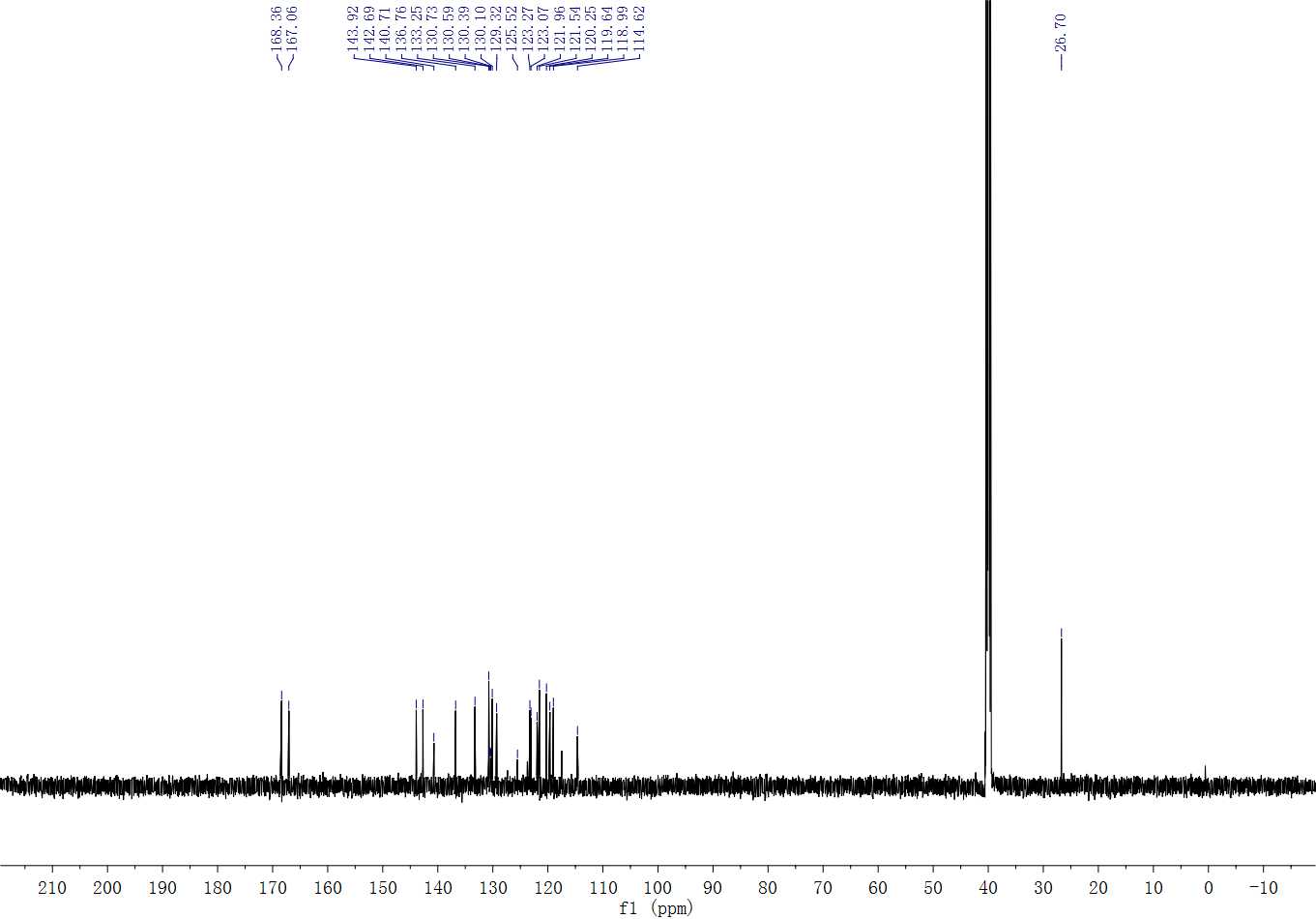
**

**Compd. B9**

**
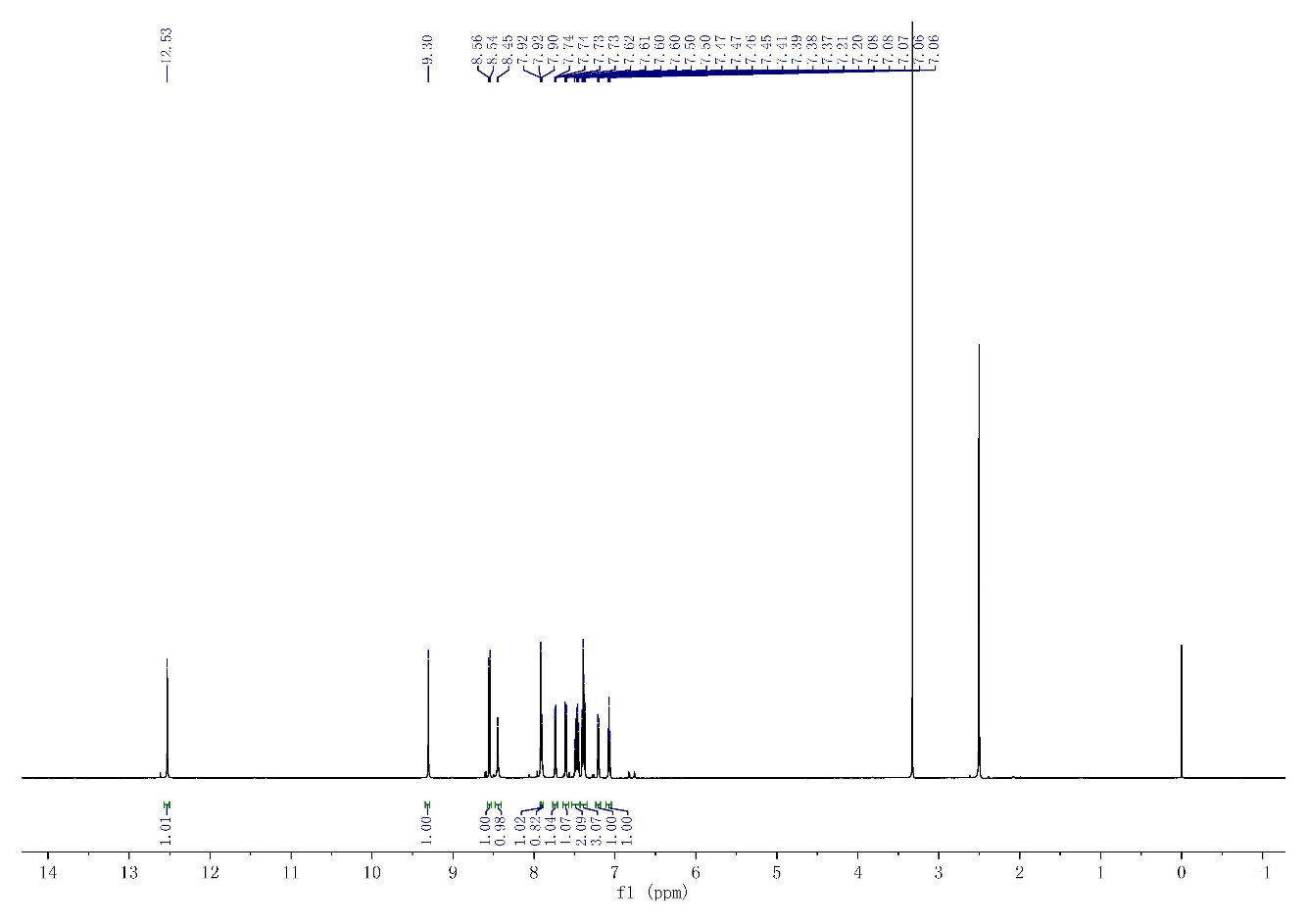

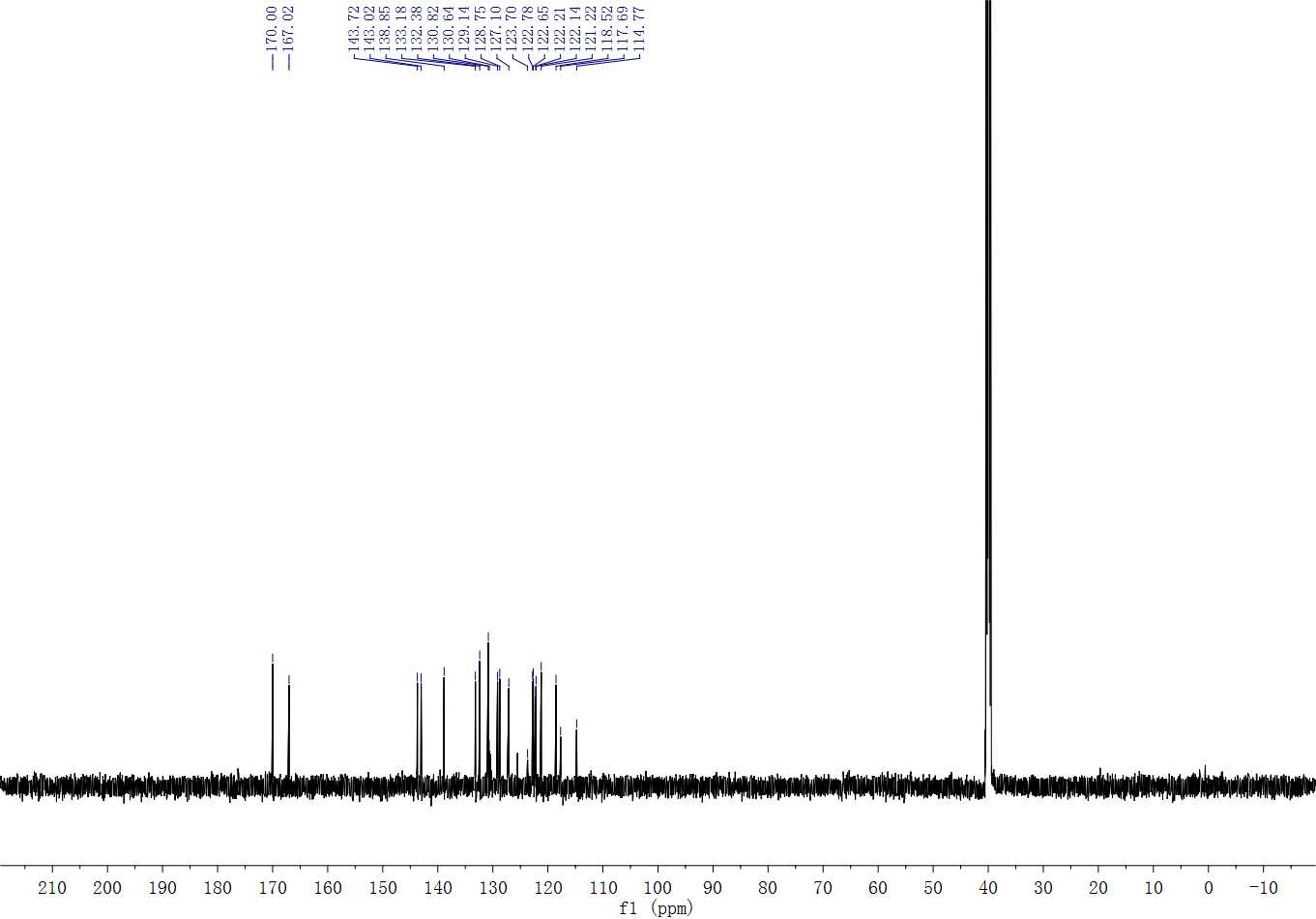
**

**Compd. B10**

**
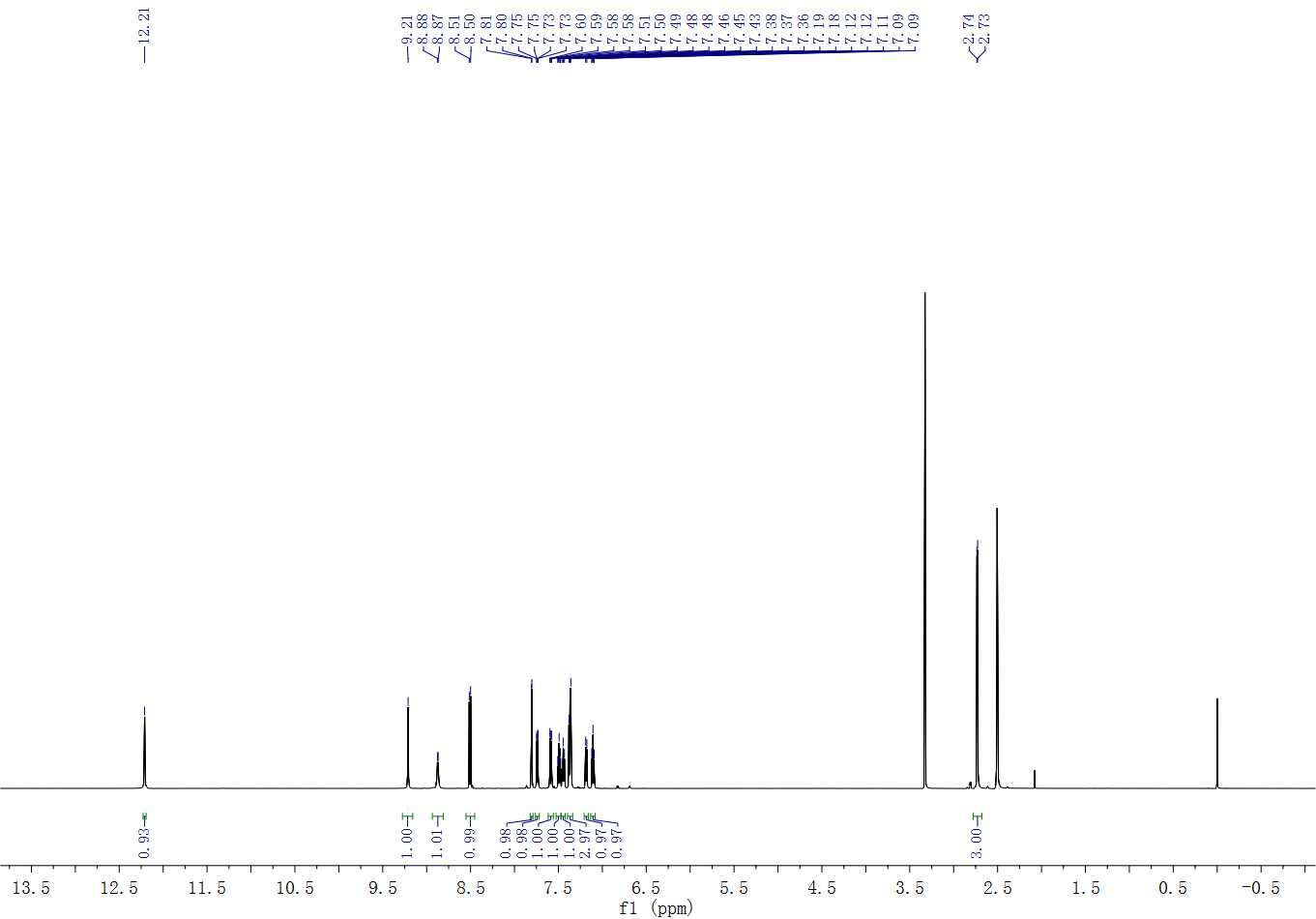

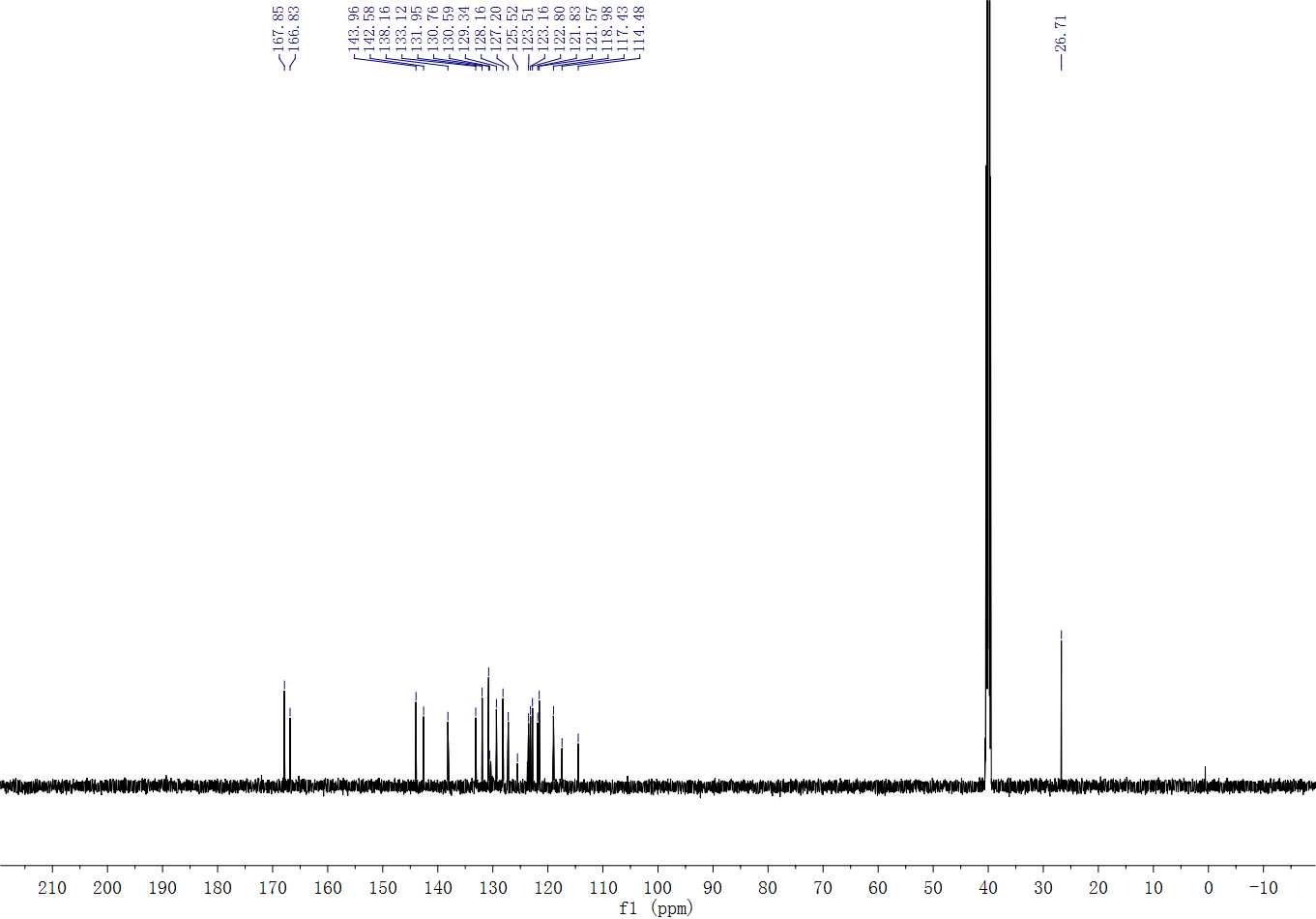
**

**Compd. B11**

**
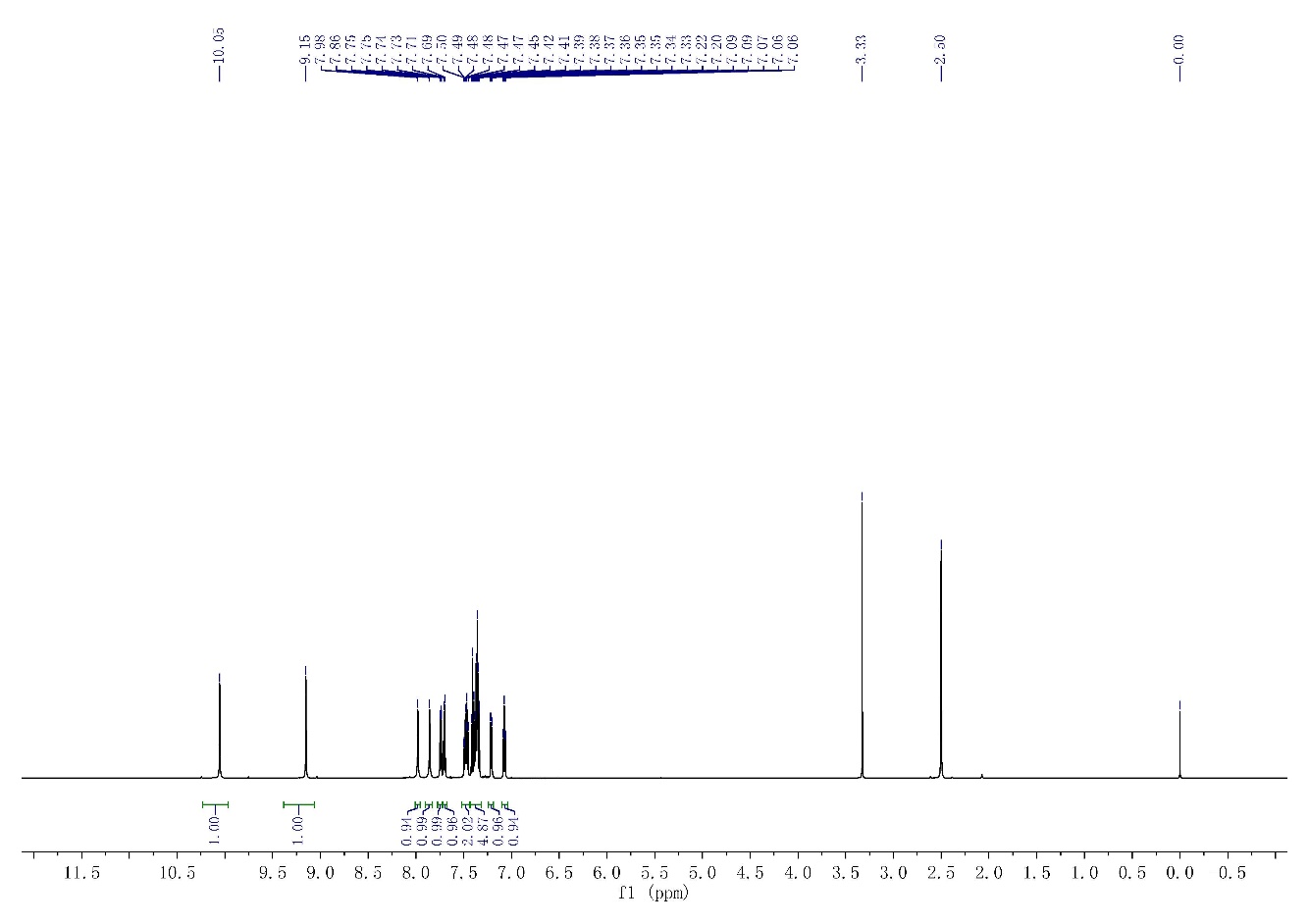

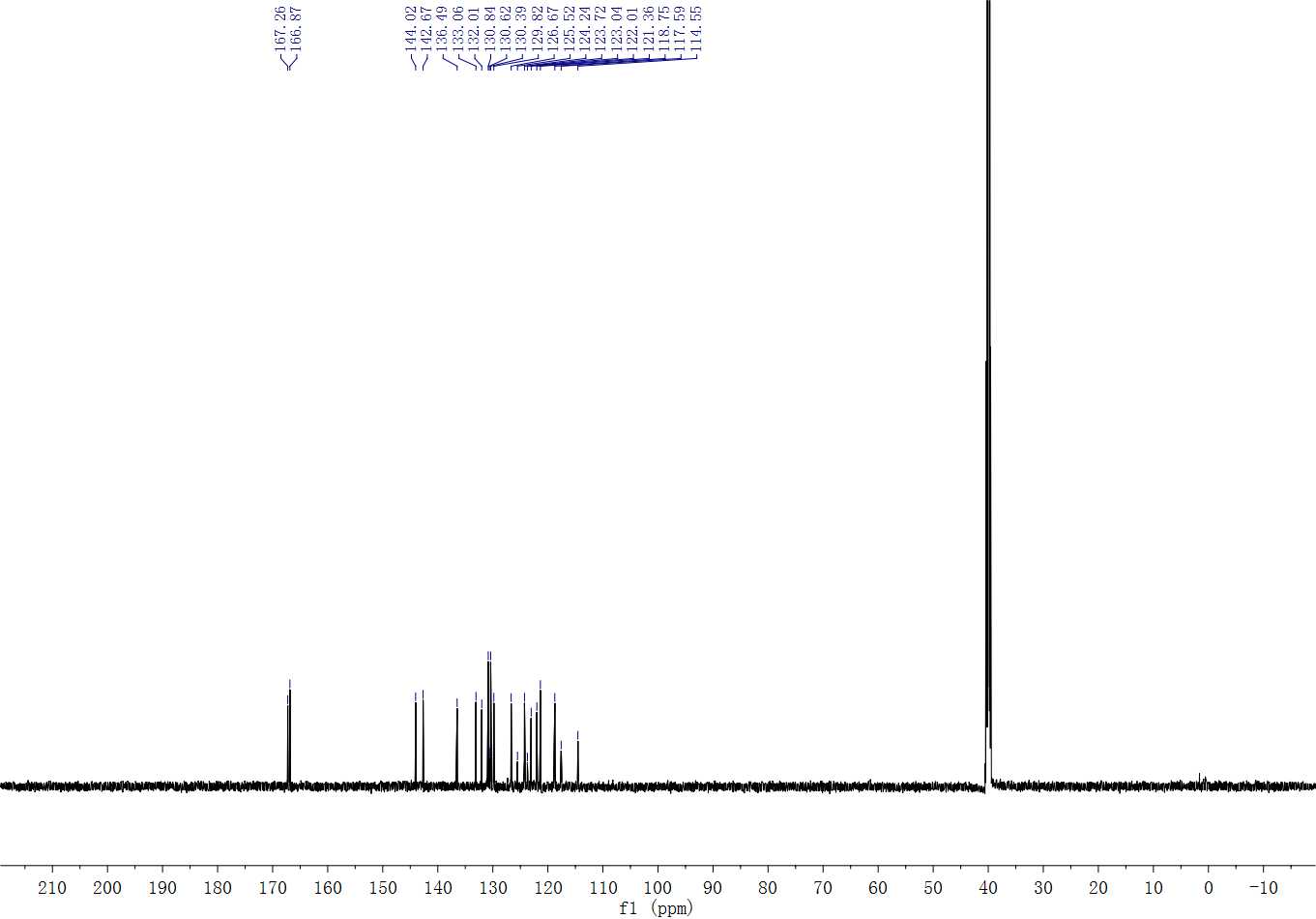
**

**Compd. B12**

**
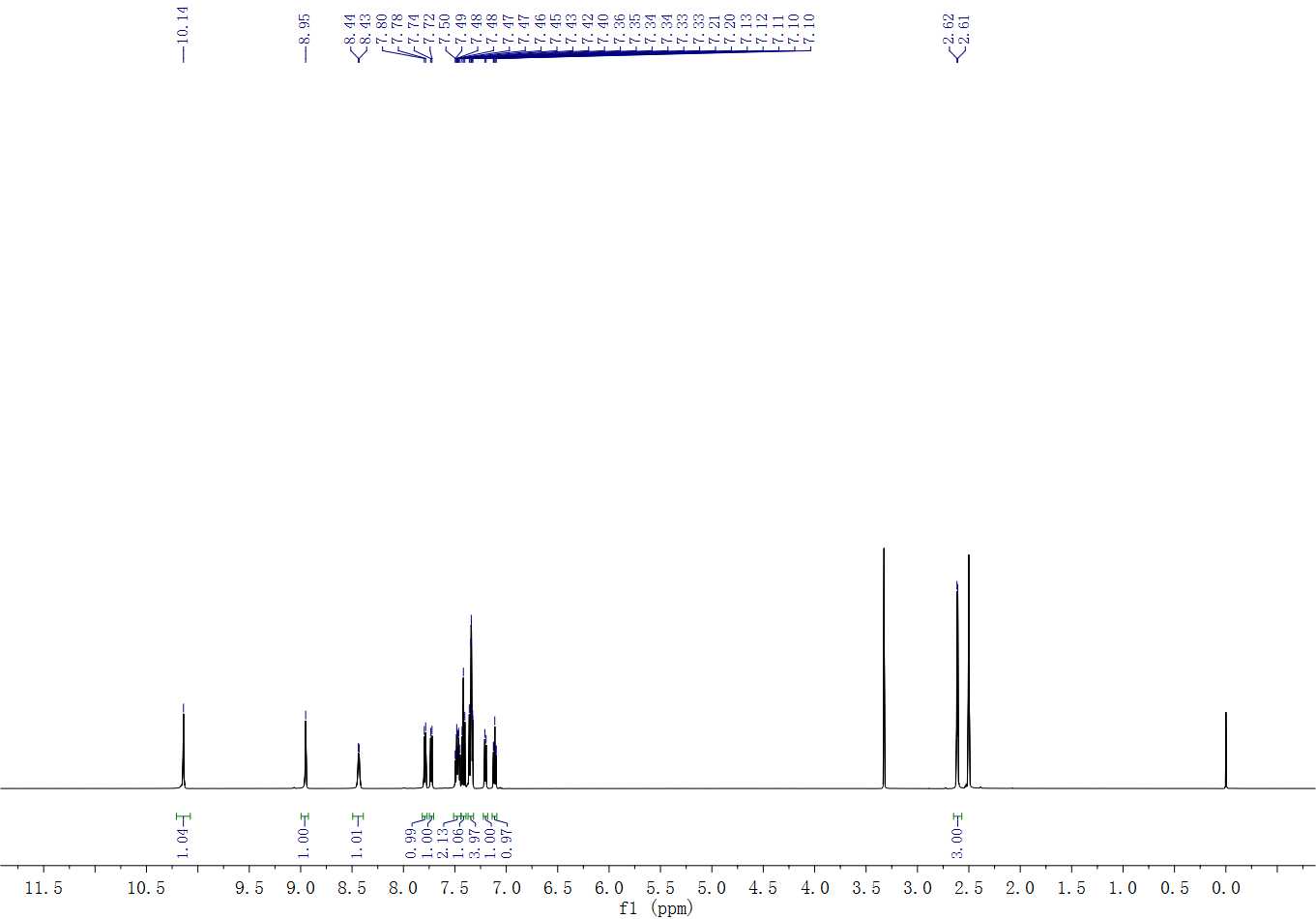

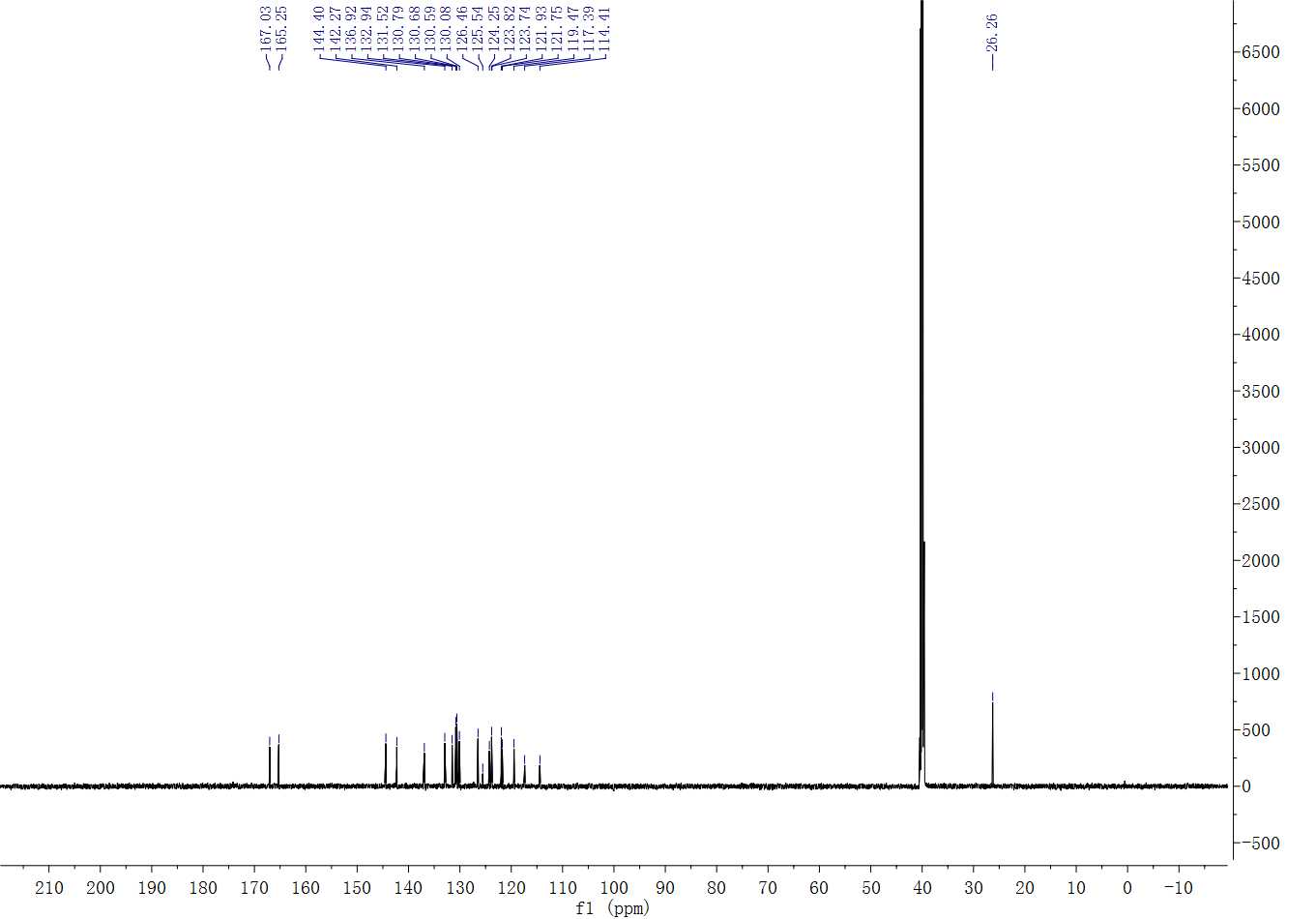
**

**Compd. B13**

**
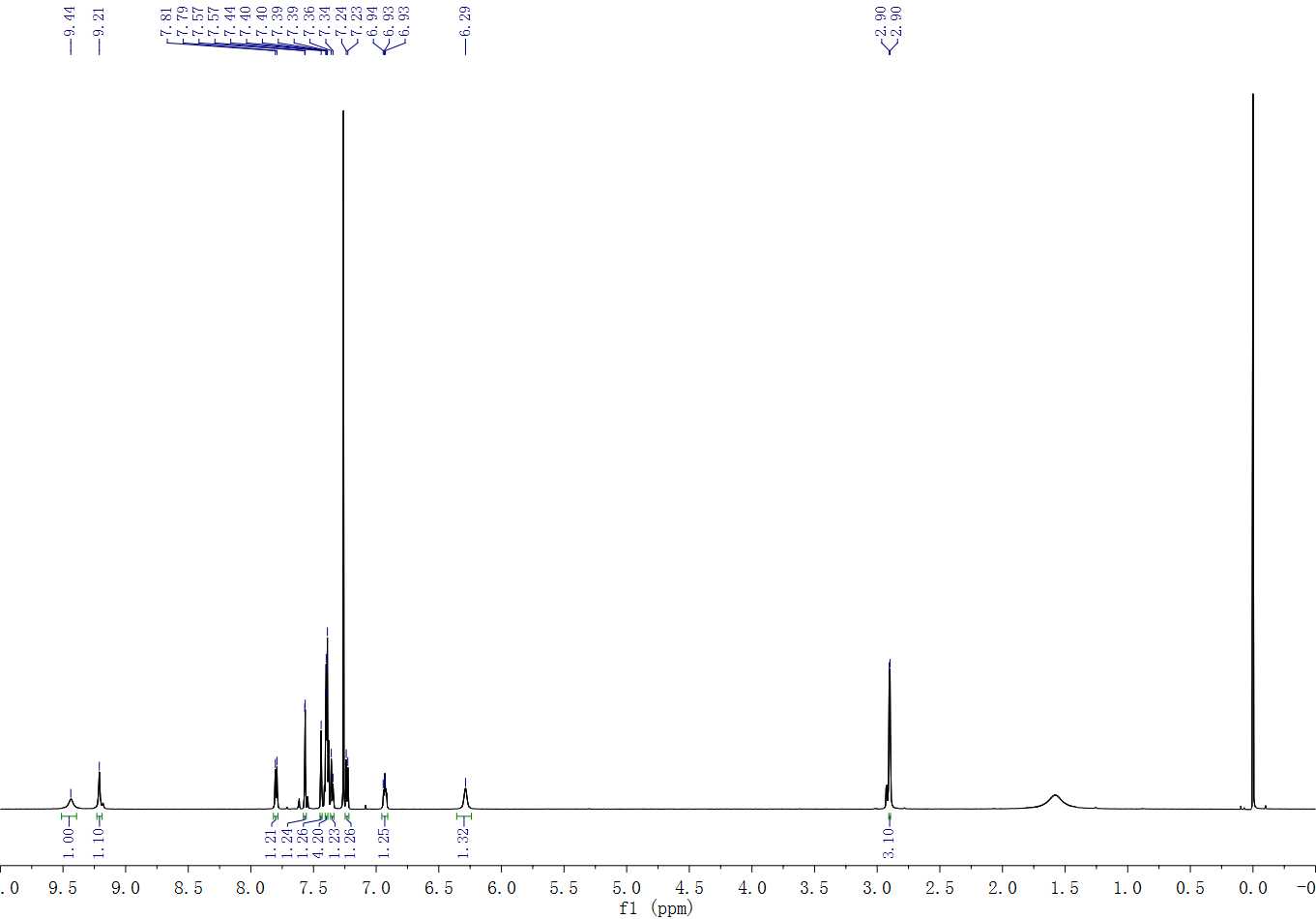

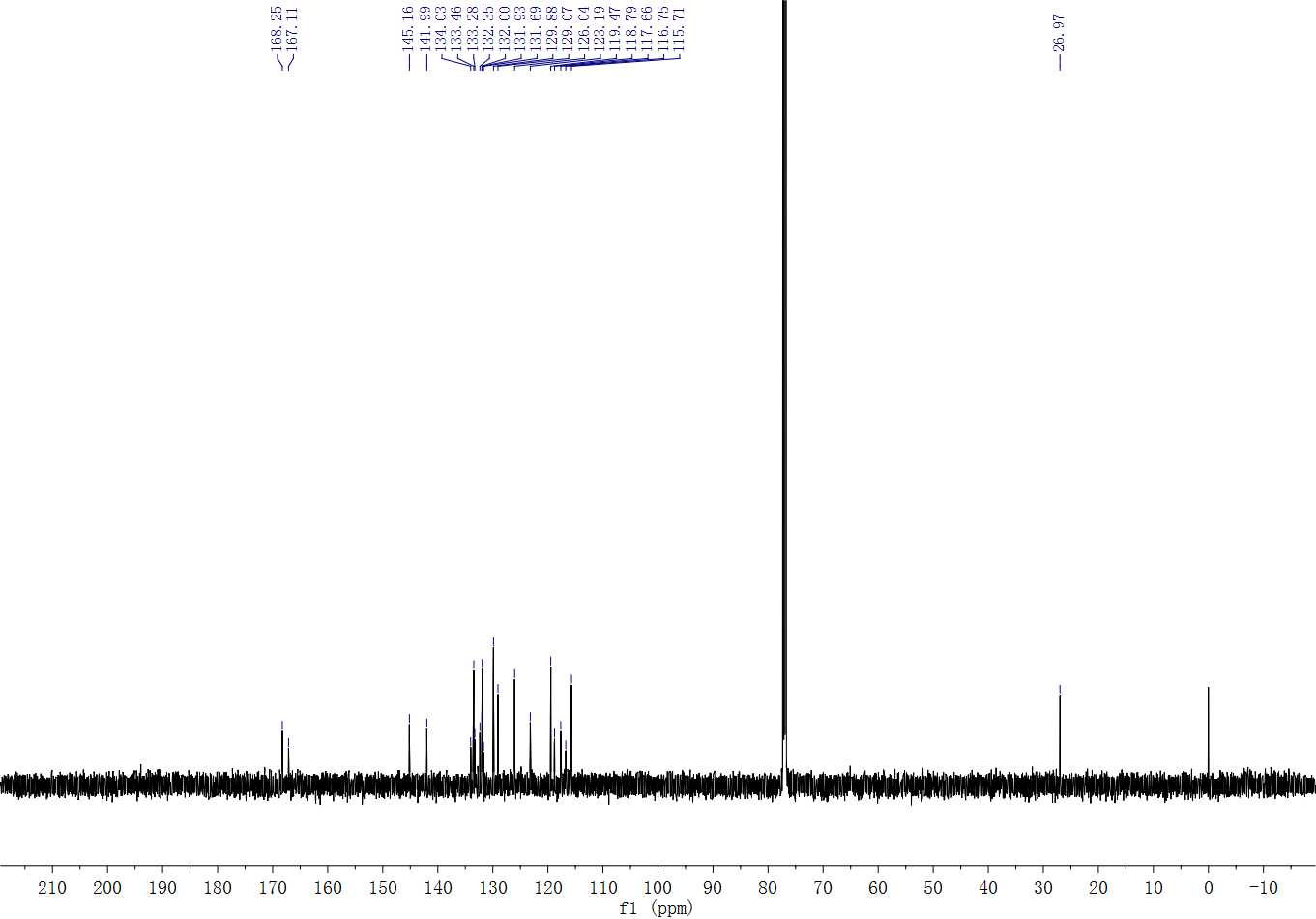
**

**Compd. C1**

**
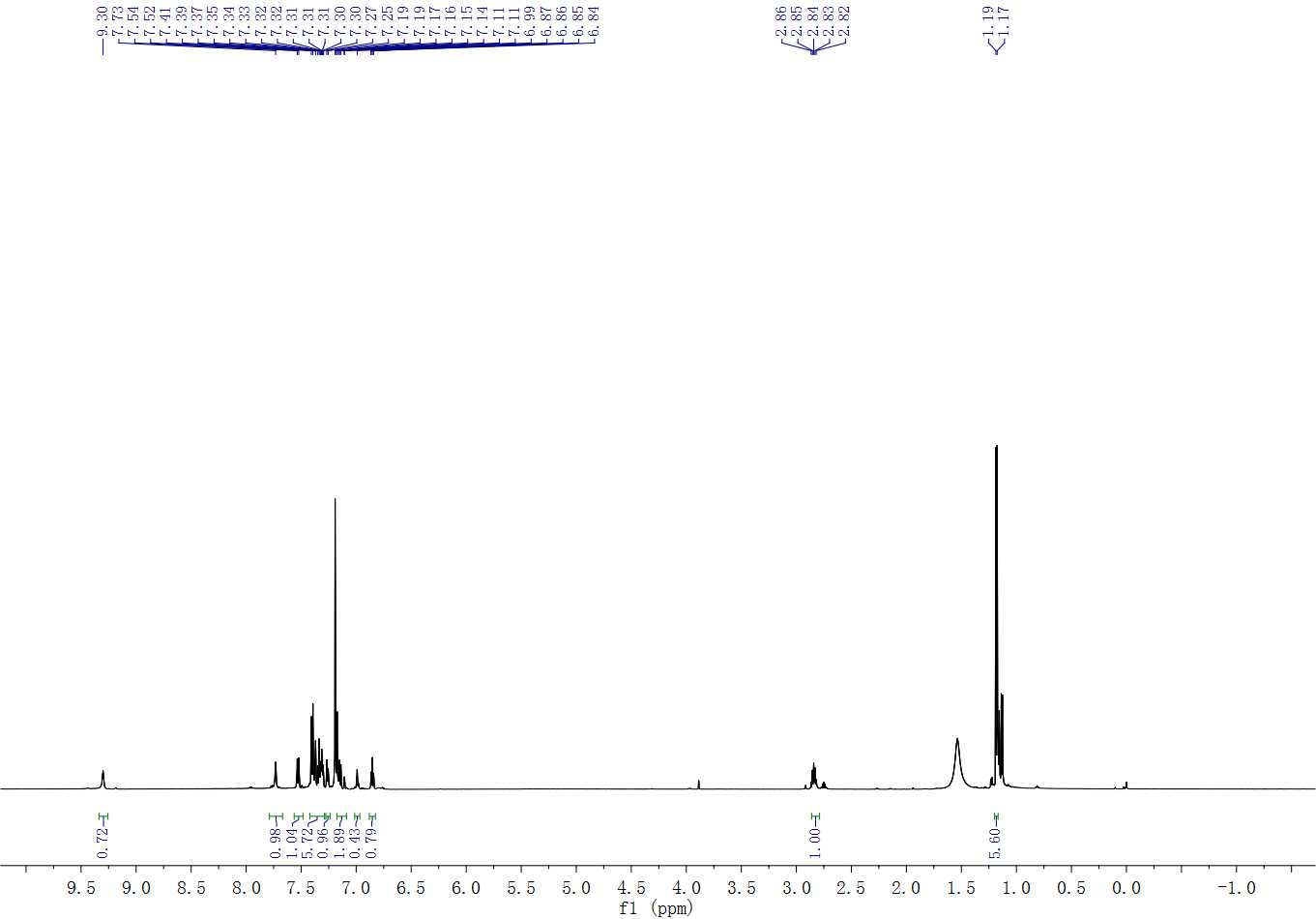

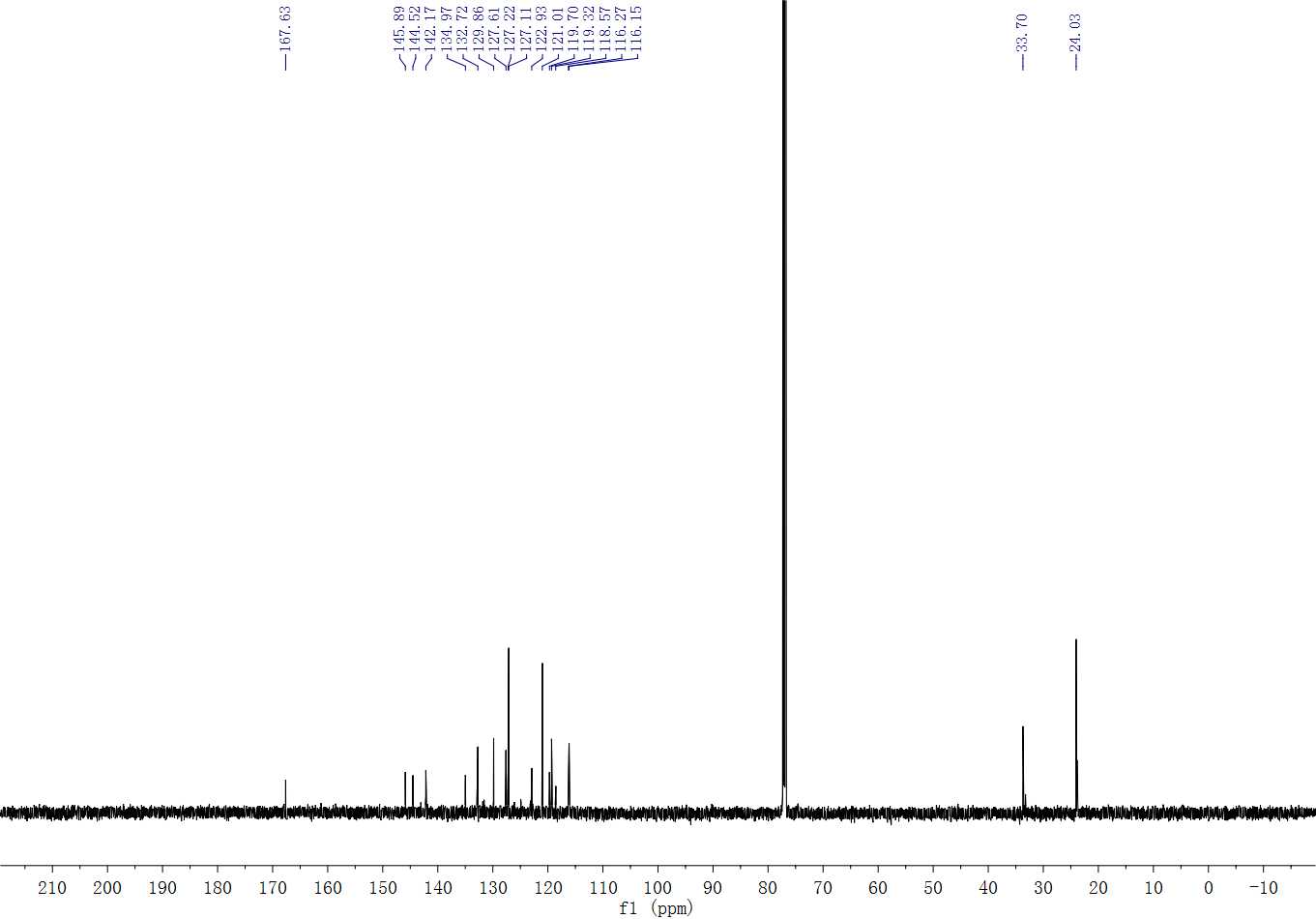
**

**Compd. C2**

**
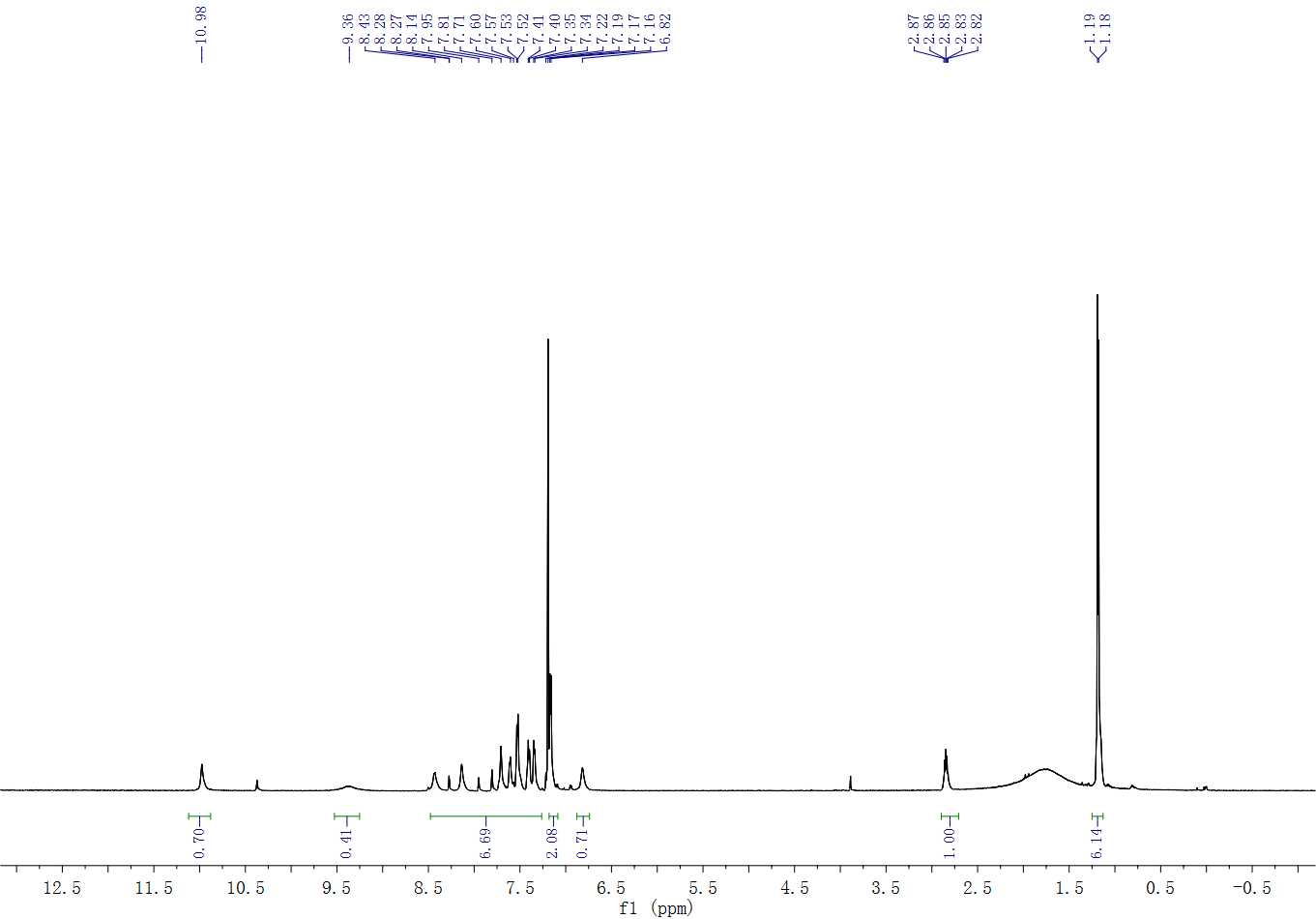
**

**
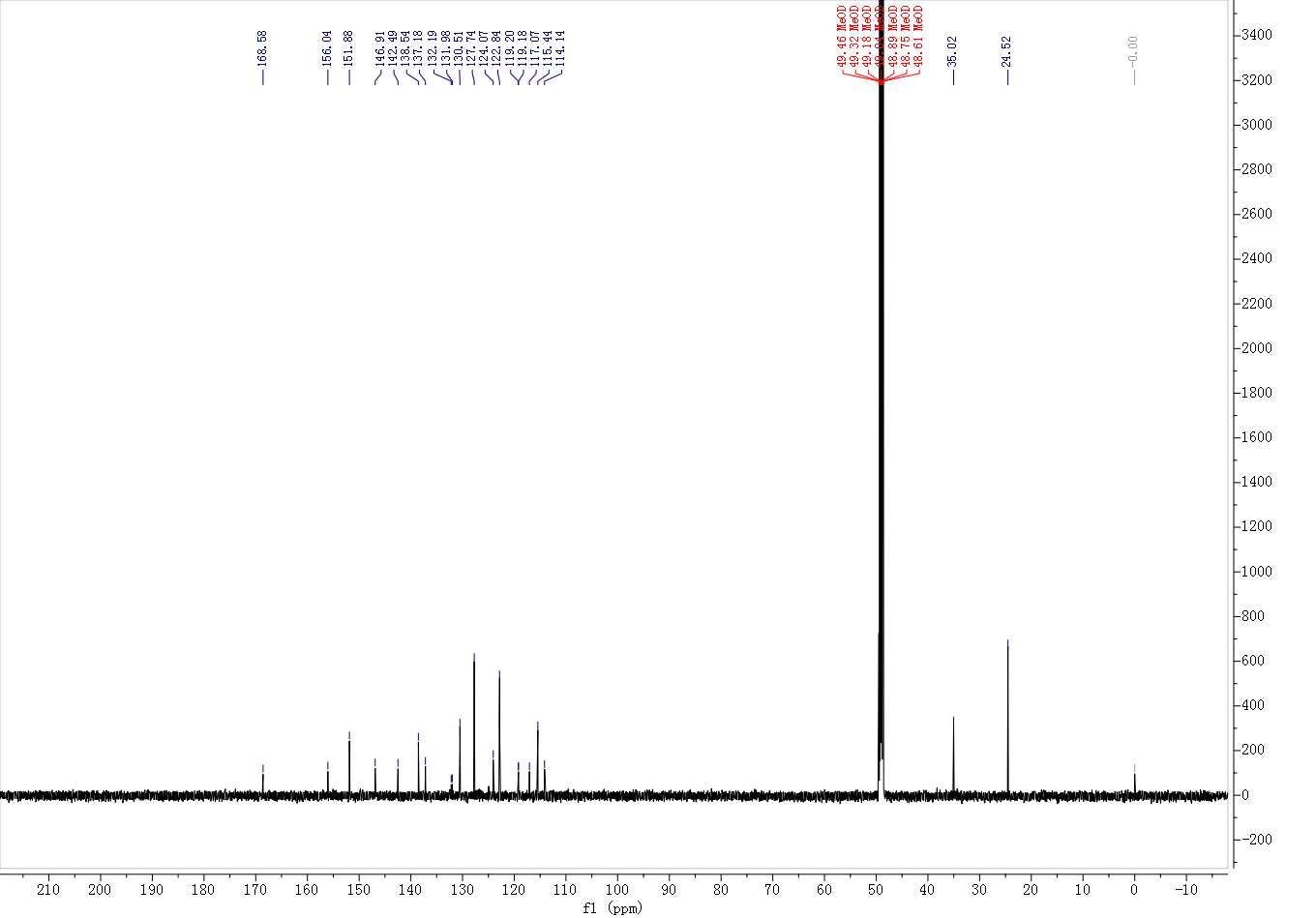
**

**Compd. C3**

**
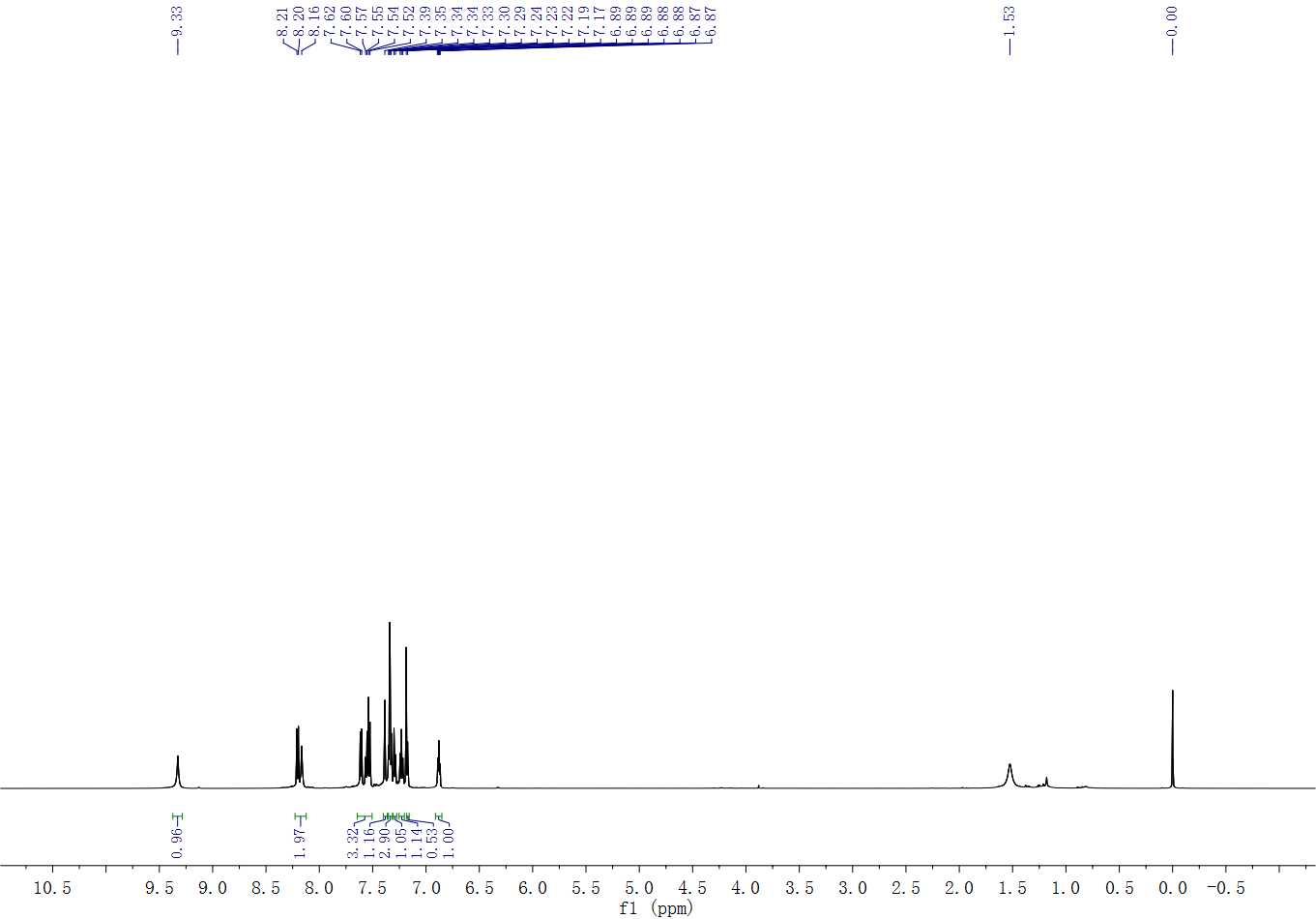

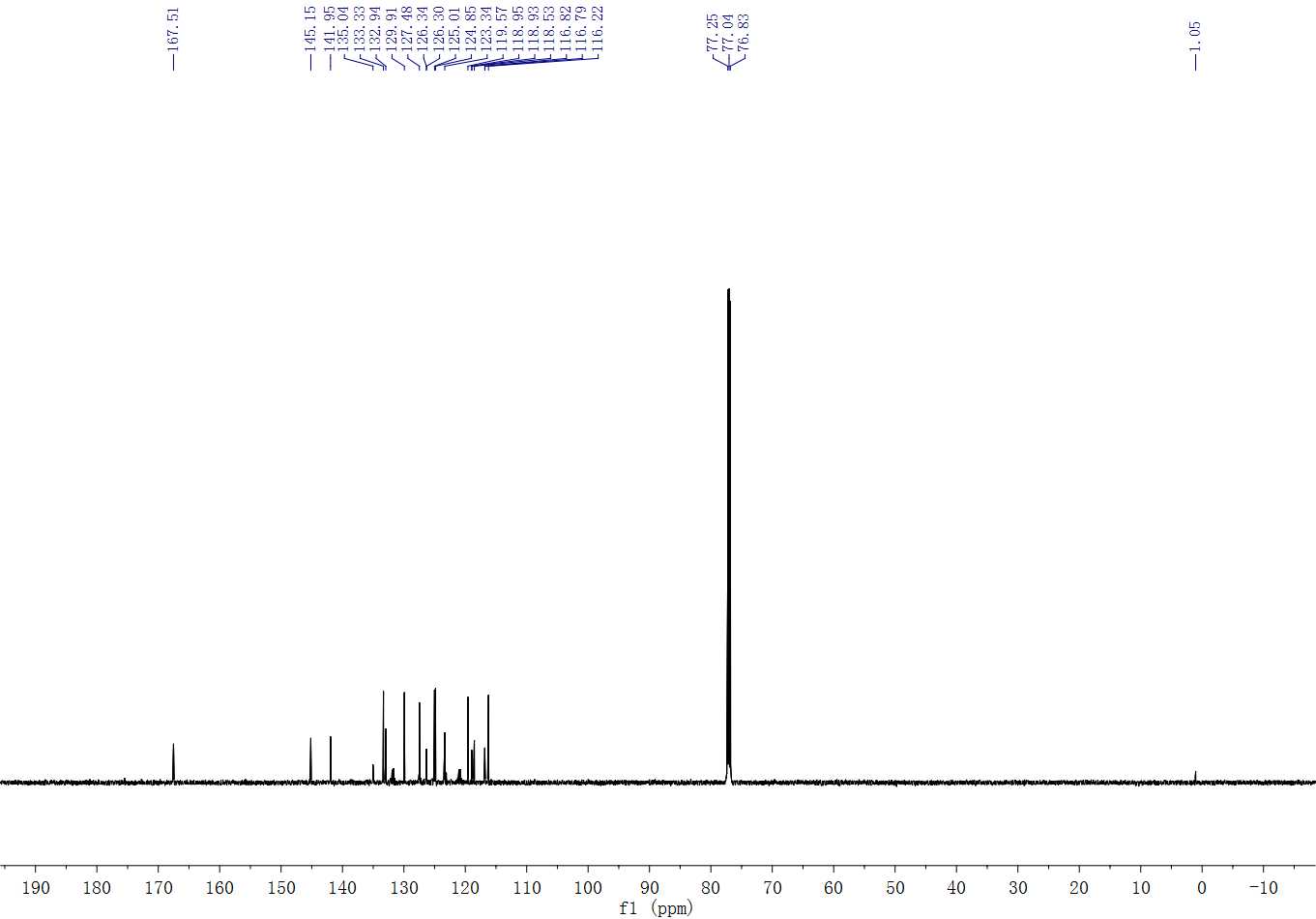
**

**Compd. C4**

**
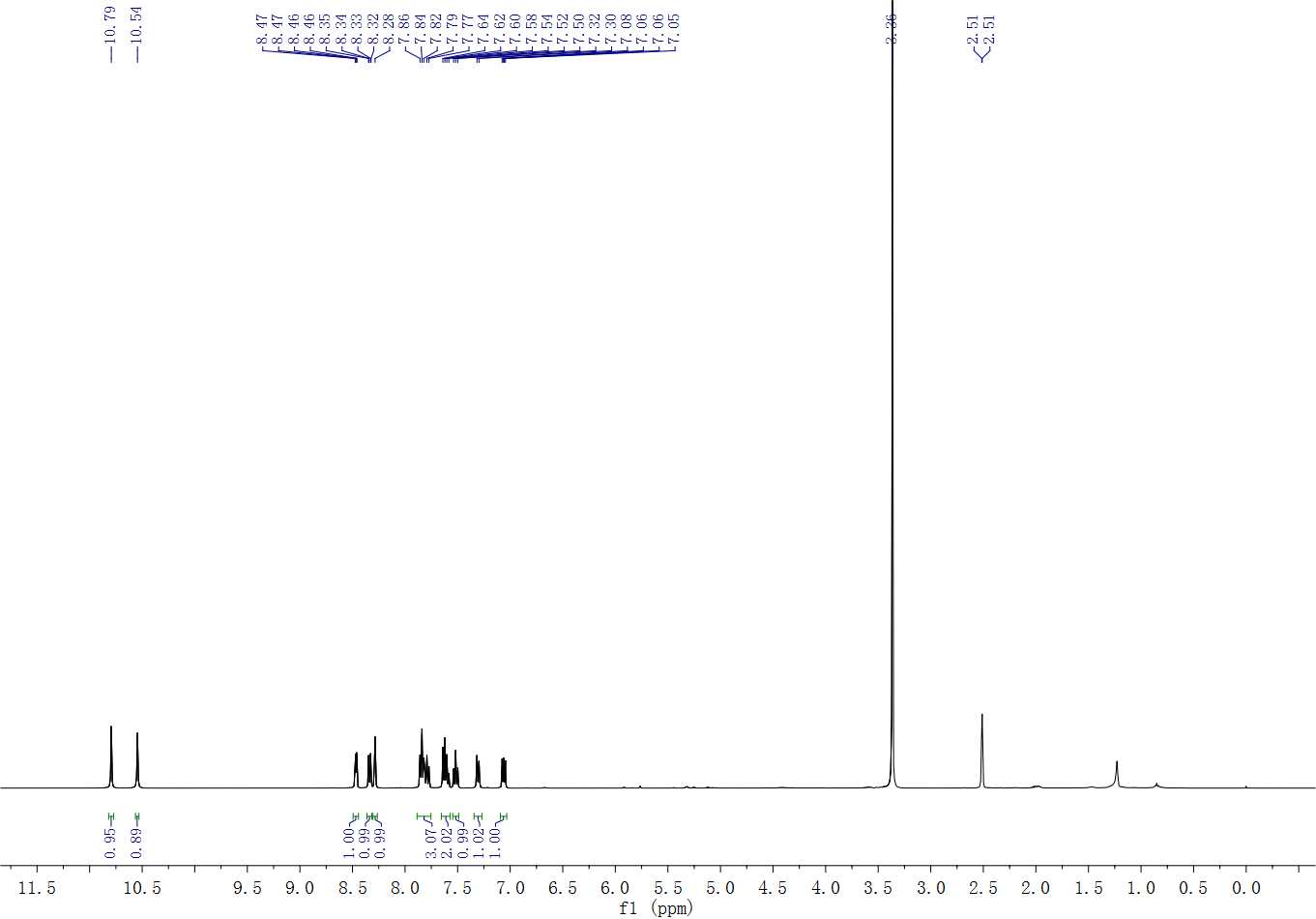

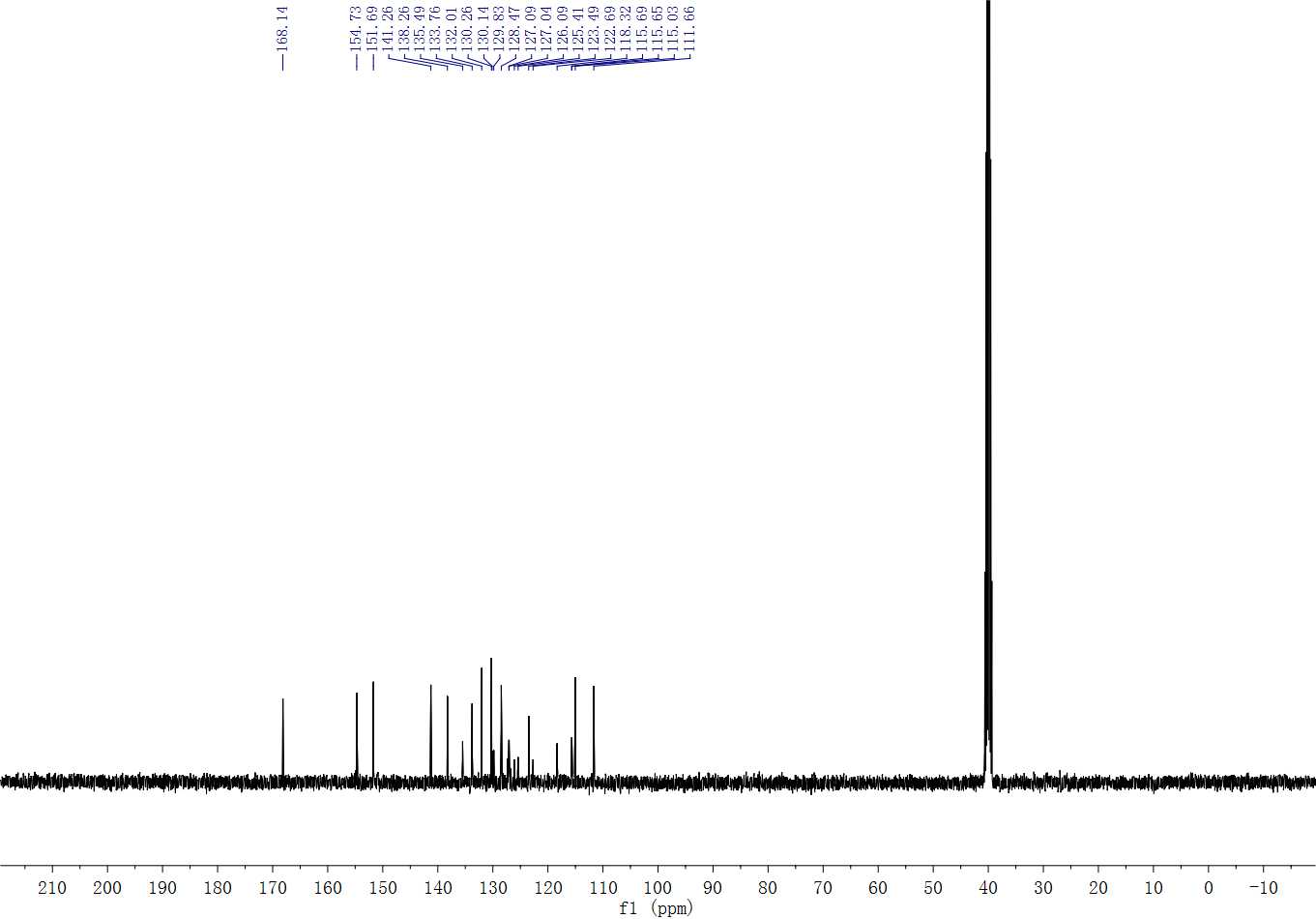
**

**Compd. C5**

**
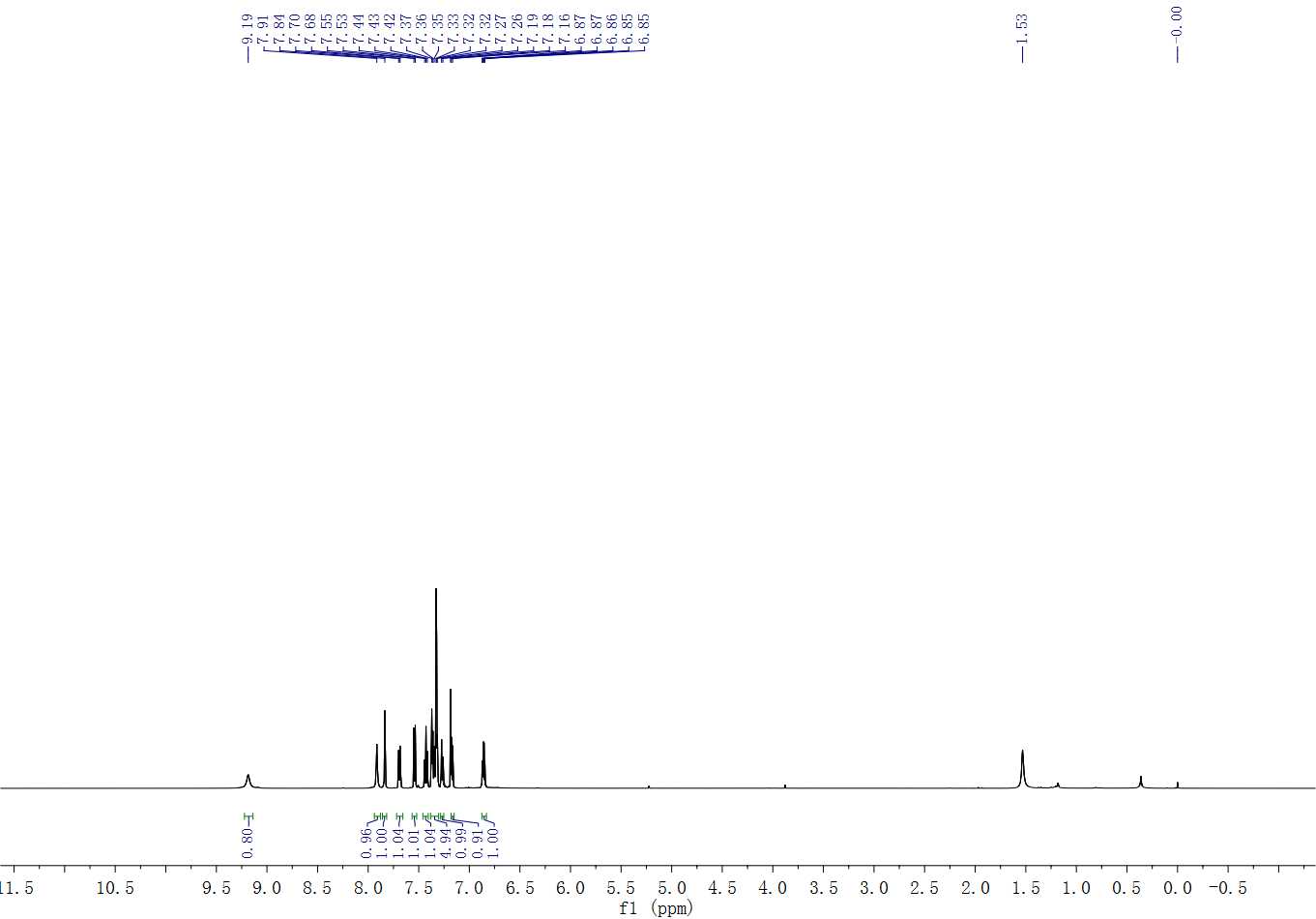

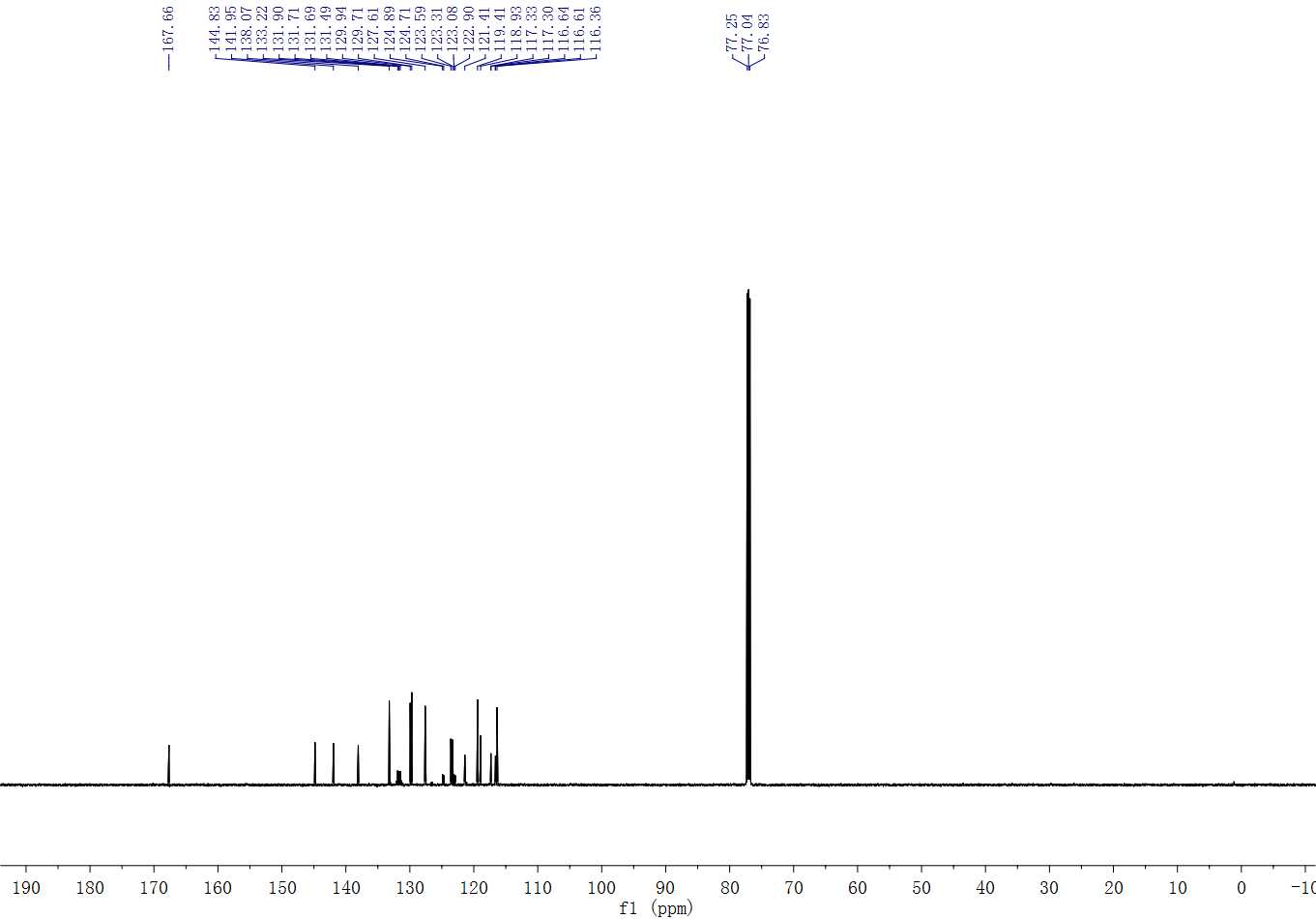
**

**Compd. C6**

**
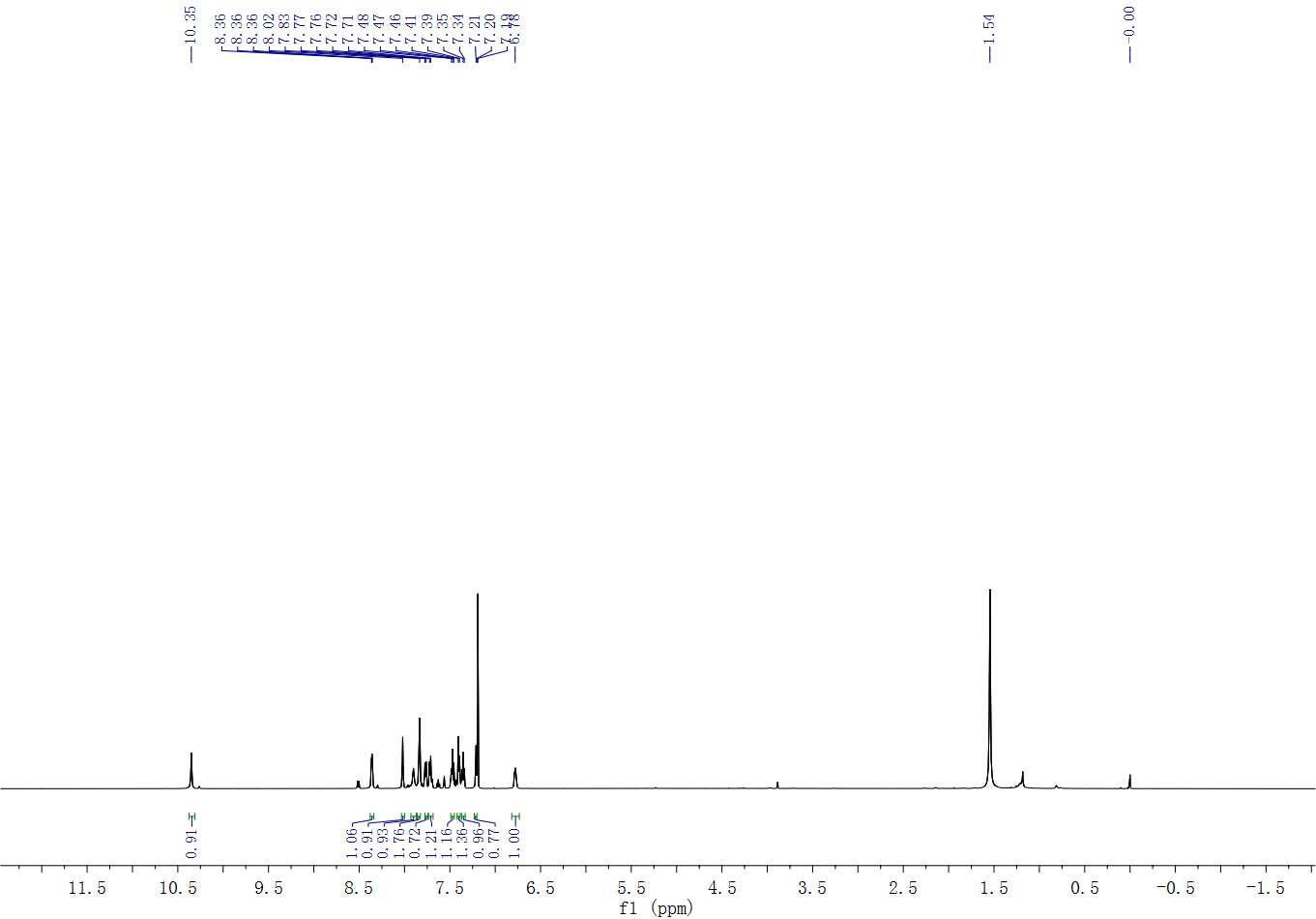

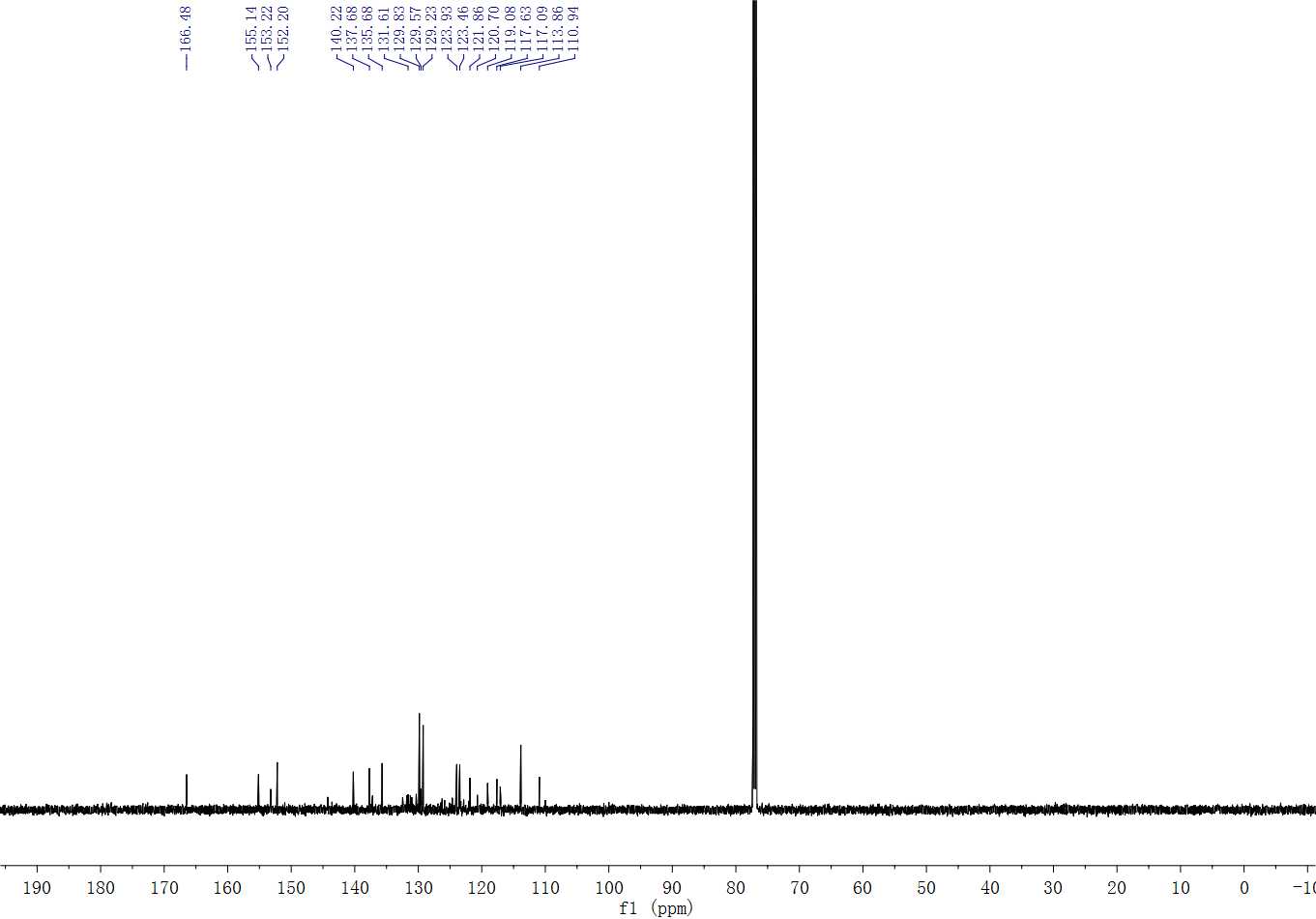
**

**Compd. C7**

**
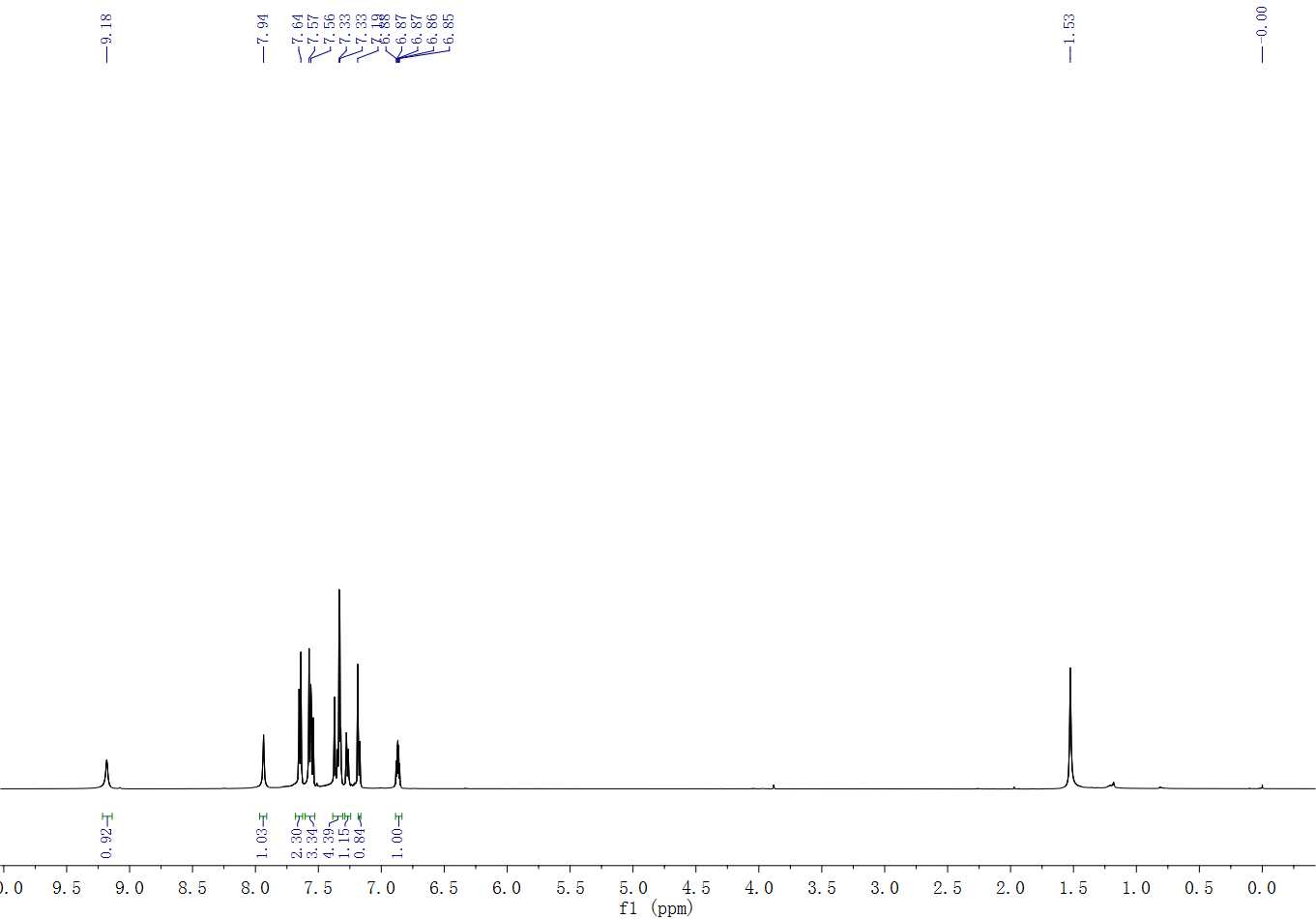

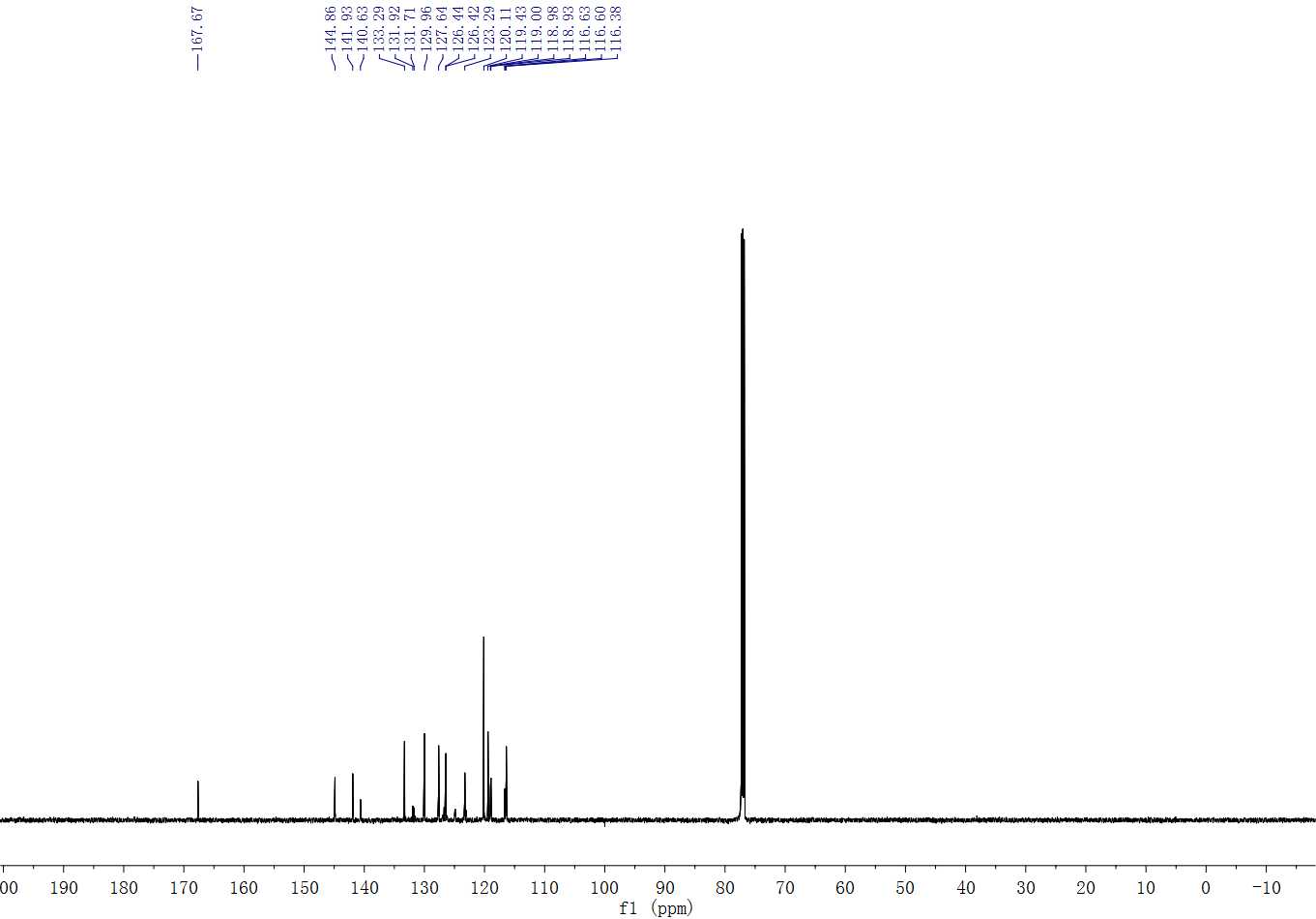
**

**Compd. C8**

**
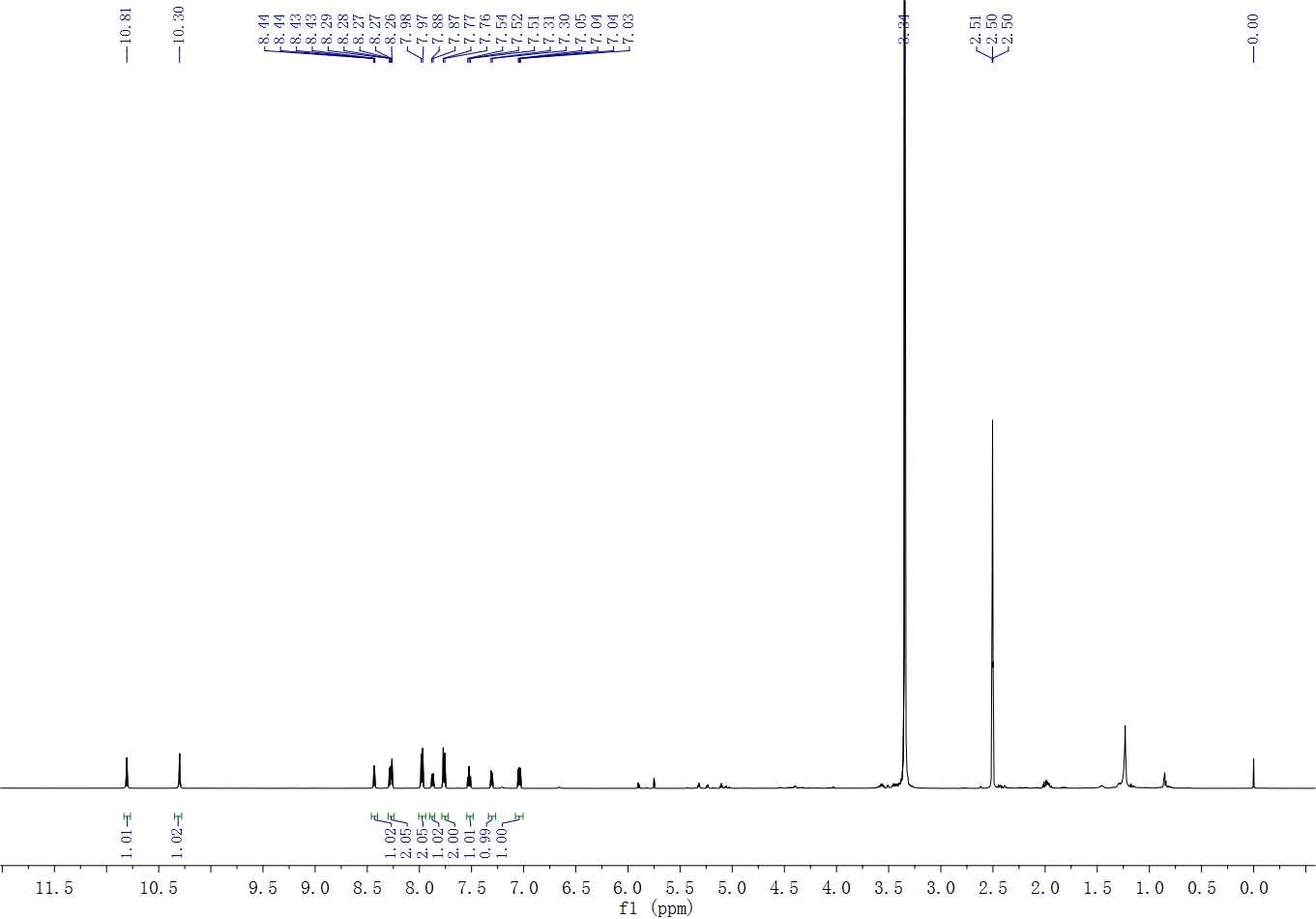
**

**
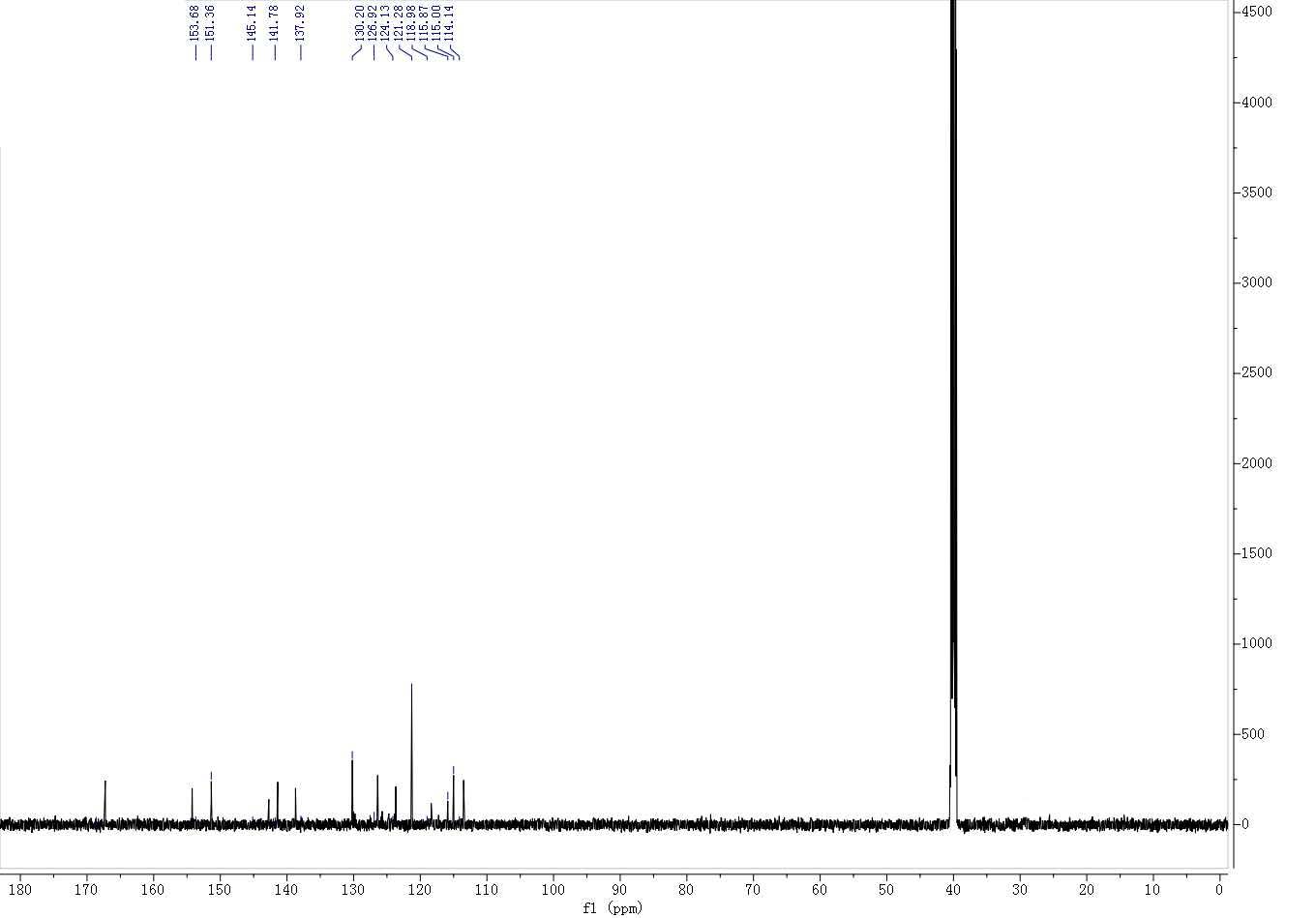
**

**Compd. C9**

**
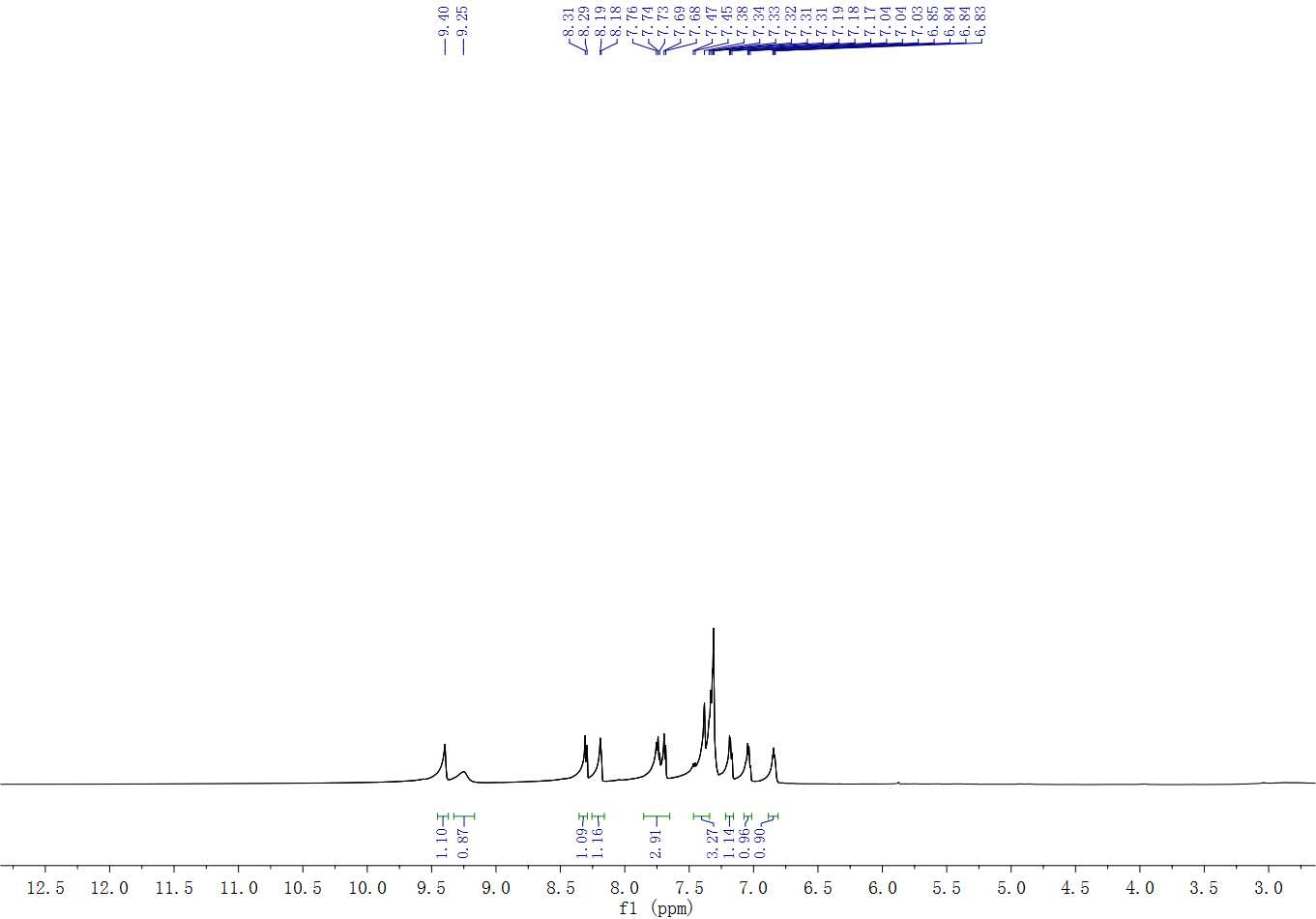

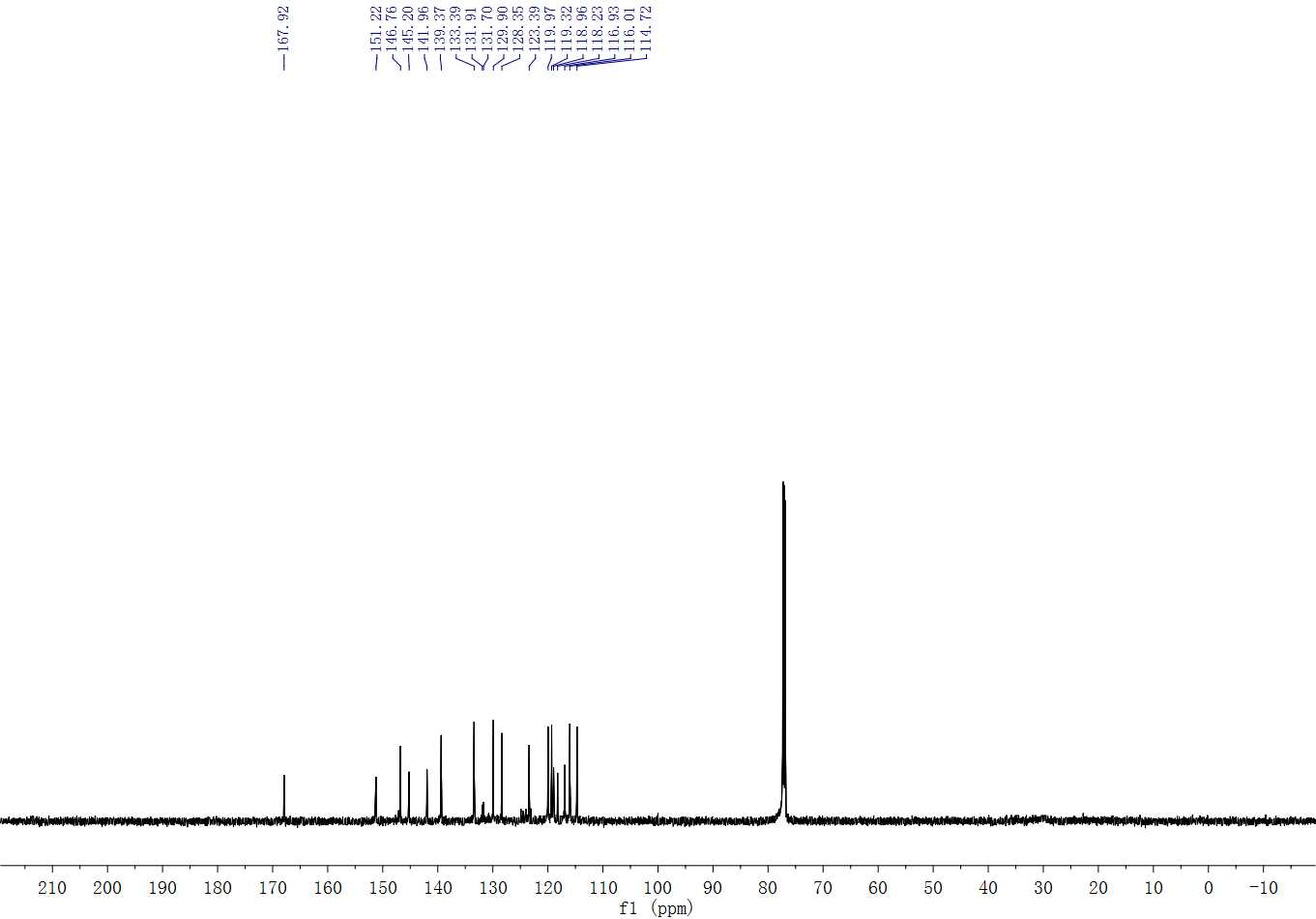
**

**Compd. C10**

**
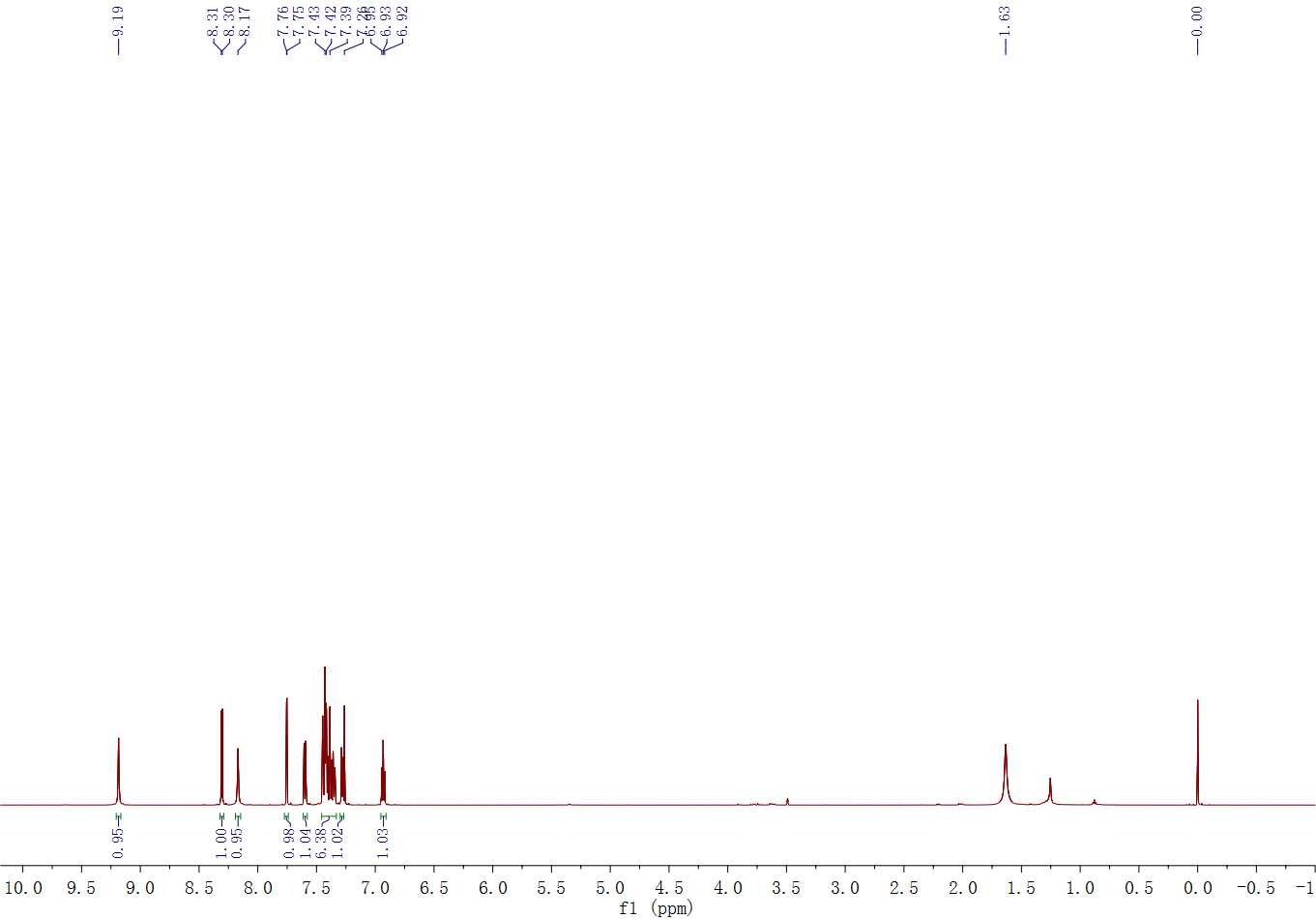

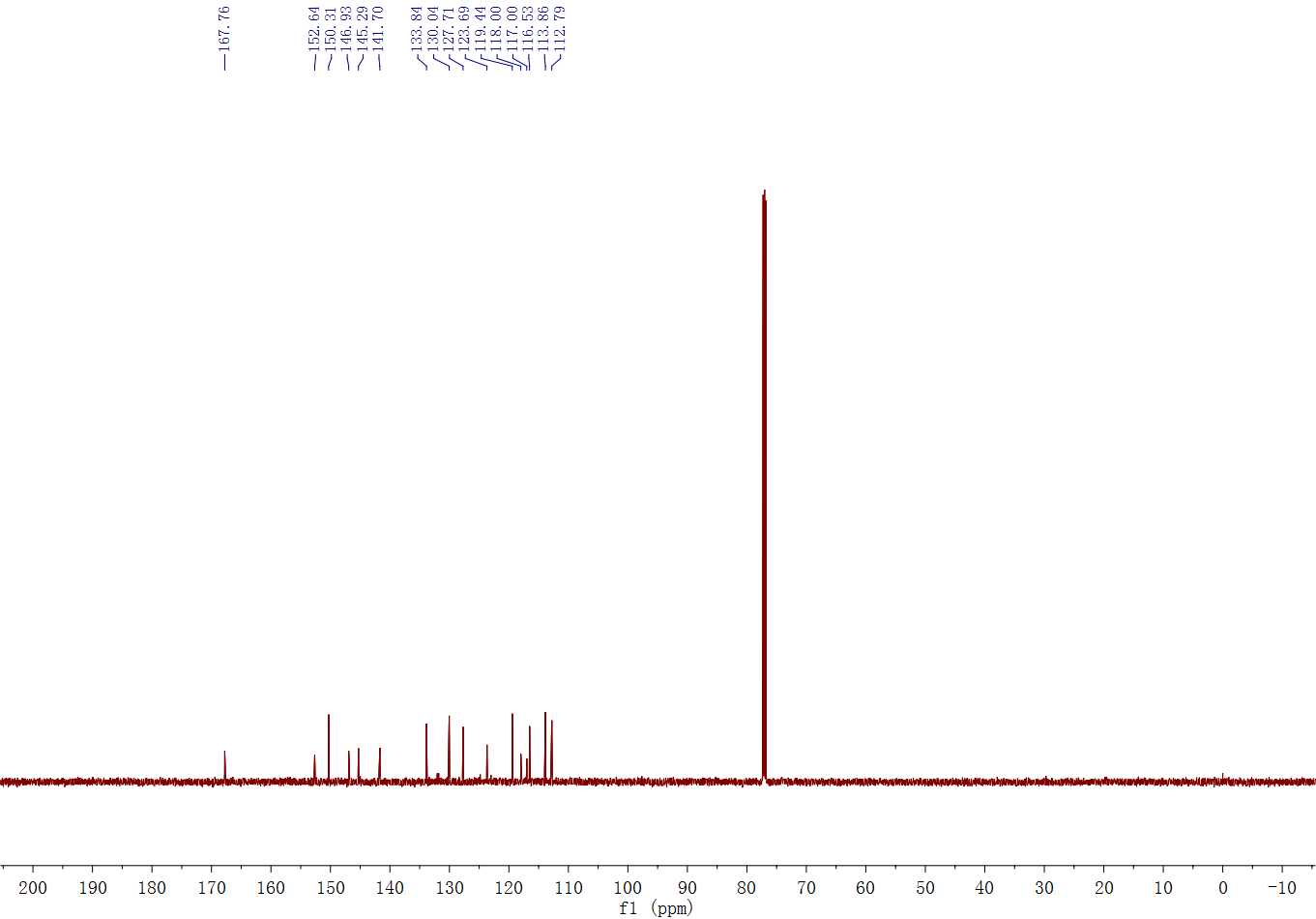
**

**Compd. C11**

**
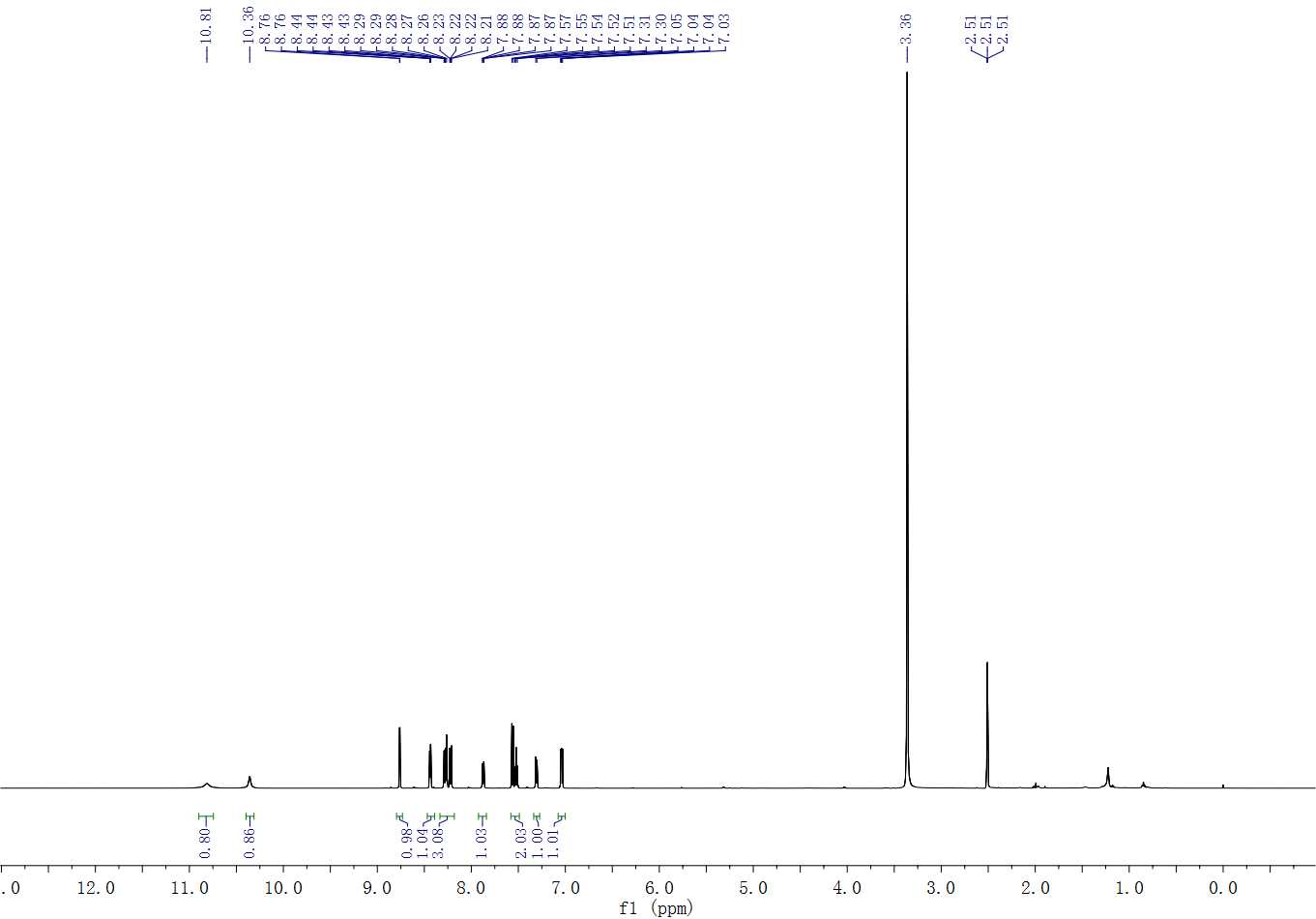

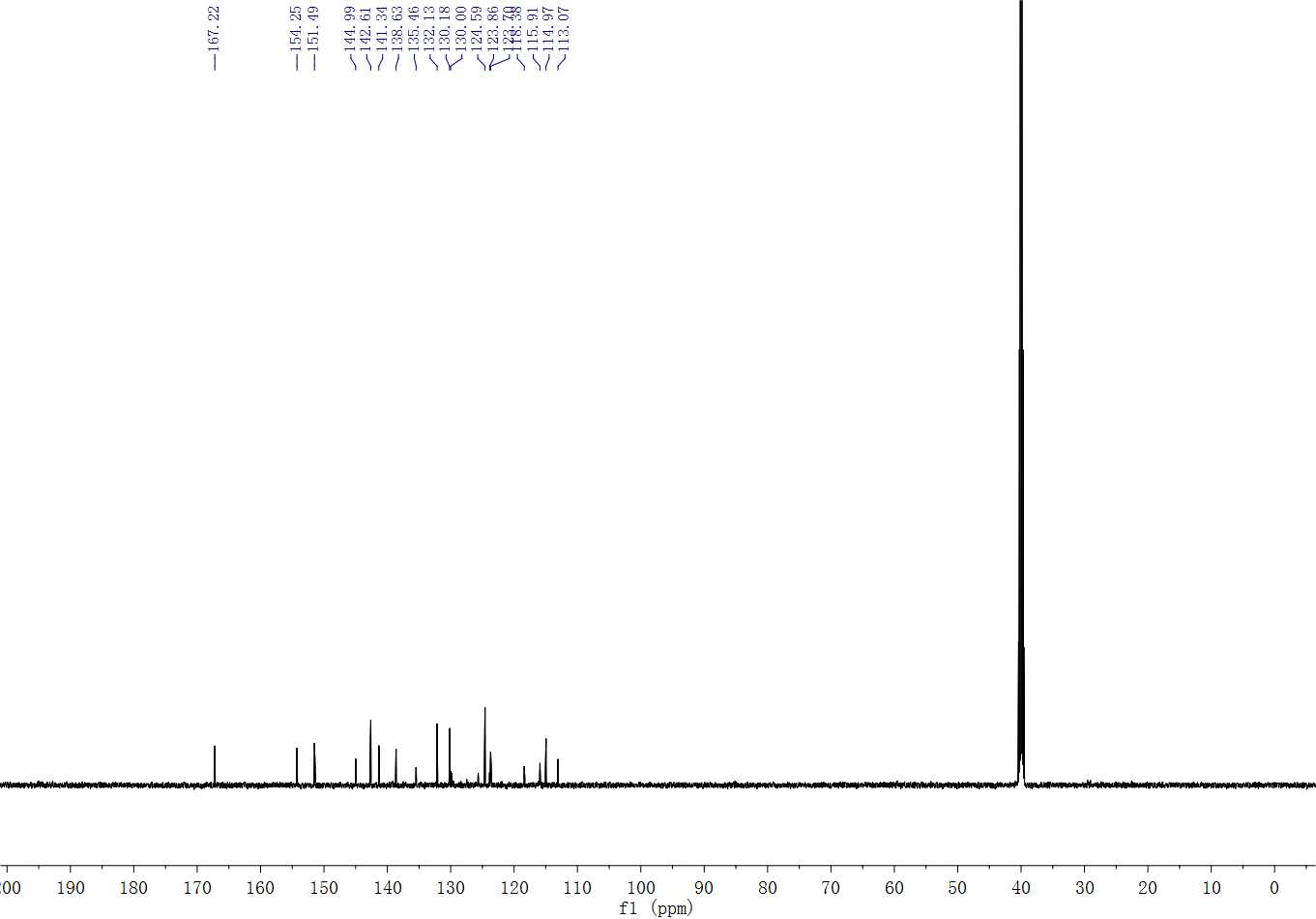
**
